# The care cascade of chronic obstructive pulmonary disease in China: a cross-sectional study of individual-level data at enrolment into the national ‘Happy Breathing’ Programme

**DOI:** 10.1016/j.eclinm.2024.102597

**Published:** 2024-07-16

**Authors:** Chen Wang, Weiran Qi, Ting Yang, Lirui Jiao, Qiushi Chen, Ke Huang, Fengyun Yu, Pascal Geldsetzer, Till Bärnighausen, Simiao Chen

**Affiliations:** a School of Population Medicine and Public Health, Chinese Academy of Medical Sciences and Peking Union Medical College, Beijing, China; b National Clinical Research Center for Respiratory Diseases, Beijing, China; c Department of Pulmonary and Critical Care Medicine, Center of Respiratory Medicine, China–Japan Friendship Hospital, Beijing, China; d Columbia Mailman School of Public Health, New York, USA; e The Harold and Inge Marcus Department of Industrial and Manufacturing Engineering, The Pennsylvania State University, University Park, PA, USA; f Heidelberg Institute of Global Health (HIGH), Faculty of Medicine and University Hospital, Heidelberg University, Heidelberg, Germany; g Chan Zuckerberg Biohub, San Francisco, CA, USA; h Division of Primary Care and Population Health, Department of Medicine, Stanford University School of Medicine, Stanford, CA, USA; i Harvard Center for Population and Development Studies, Harvard University, Cambridge, MA, USA

**Keywords:** Chronic obstructive pulmonary disease, Care cascade, Health system, Unmet need, Health management

## Abstract

**Background:**

Understanding the chronic obstructive pulmonary disease (COPD) care cascade is crucial for identifying where and when to intervene to improve COPD outcomes. We aimed to determine the proportion of patients with COPD seeking care in China's health system who are lost at each stage of the COPD care cascade and how the patterns of loss vary across geographical regions and population groups.

**Methods:**

From November 3, 2018, to April 22, 2021, we used individual-level patient data from the national Chinese ‘Happy Breathing’ Programme, which aims to identify patients with COPD and provide appropriate care. COPD was defined as a post-bronchodilator ratio of forced expiratory volume in 1 s to forced vital capacity (FEV1/FVC) <0.70. We calculated the proportions of individuals who, at enrolment into the ‘Happy Breathing’ Programme, (i) had ever undergone a pulmonary function test, (ii) had been diagnosed with COPD in the past, (iii) were currently on treatment for COPD, and (iv) had achieved control of their COPD. We examined the association between reaching each stage of the care cascade and individual patient characteristics as well as regional-level economic development and available resources in the health system using multilevel regression.

**Findings:**

Among the 29,201 patients with COPD in the ‘Happy Breathing’ Programme, 41.0% (95% confidence interval [CI]: 40.4–41.6%) had ever been tested for COPD, 17.6% (95% CI: 17.1–18.0%) had previously been diagnosed with COPD, 8.5% (95% CI: 8.2–8.8%) were currently on treatment for COPD, 4.6% (95% CI: 4.3–4.8%) of patients had mild or no exacerbations in the prior year, and 3.9% (95% CI: 3.7–4.2%) of patients had suffered no exacerbations in the prior year. On average, patients living in the cities of Beijing, Wuhan, and Yinchuan had progressed further along the COPD care cascade than patients living in Daqing and Luoyang. Using multilevel regression, we found that young age, rural residence, and low regional per-capita GDP were significantly associated with larger losses at each stage of the COPD care cascade.

**Interpretation:**

Substantial proportions of patients with COPD are lost at each stage of the COPD care cascade in the Chinese health system. The largest losses occur during the initial stages of the cascade, when diagnosis first occurs. New policies and interventions are required to boost COPD care, especially screening and diagnosis, in the Chinese health system to reduce this large disease burden.

**Funding:**

This work was supported by Major Programme of 10.13039/501100001809National Natural Science Foundation of China (82090011), CAMS Innovation Fund for Medical Sciences (CIFMS) (2021-I2M-1-049), and 10.13039/100018693Horizon Europe (HORIZON-MSCA-2021-SE-01; project number 101086139-PoPMeD-SuSDeV). TB was supported by the 10.13039/100005156Alexander von Humboldt Foundation through the Alexander von Humboldt professorship award.

**Research in context**

**Evidence before this study**

We searched PUBMED for work published between January 1, 1966, and July 25, 2022, with the search terms “chronic obstructive pulmonary disease” OR “COPD” and “care cascade” OR “unmet need,” with variations of the search terms “screened,” “aware,” “diagnosed,” “treated,” OR “controlled” in either the title or the abstract. Several of the returned studies mentioned stages of the chronic obstructive pulmonary disease (COPD) care cascade, such as awareness of disease status or receipt of treatment, but no single study had systematically explored how the health system performed with regards to the entire COPD care cascade. In addition, our search did not identify a single national study on the COPD care cascade in China.

**Added value of this study**

To our knowledge, this is the first study of individual-level data to comprehensively examine health system performance with respect to COPD testing, diagnosis, treatment, and control. We focus on China because it faces the highest number of deaths from COPD of any country globally. The study takes advantage of a large dataset of patients with COPD in China, collected as part of the national ‘Happy Breathing’ Programme.

Our study makes four key contributions to the existing scientific literature: First, we quantified the loss of individuals with COPD at each step of the COPD care cascade in the Chinese health system; second, we examined how COPD care cascade outcomes vary among different population groups, providing important information on possible target groups for relevant interventions; third, by benchmarking each region's performance against its per-capita gross domestic product, our analysis identified regions that performed better than expected based on their economic development and thus likely hold valuable policy lessons for other regions at a similar level of development; and finally, our study provides a benchmark of health system performance for managing COPD in China against which future progress can be measured.

**Implications of all the available evidence**

Although the proportions of patients with COPD who were tested, diagnosed, treated, and successfully treated in the Chinese health system remain low in general, performance with respect to the COPD care cascade varies widely across Chinese regions, indicating that boosting cascade coverage may be feasible in many regions. More effective and novel health services for COPD screening, testing, and diagnosis in the Chinese health system are particularly important because the largest COPD cascade losses in China occur prior to treatment initiation.

## Introduction

Chronic obstructive pulmonary disease (COPD) is a leading cause of mortality and morbidity.^1^ Worldwide, COPD is the third most common cause of death and disability-adjusted life years (DALYs). The disease caused 3.3 million deaths globally in 2019.^2^ The enormous health burden of COPD also imposes a considerable economic burden.^3^ China shoulders one of the largest COPD burdens in the world and had the largest number of COPD-related deaths in 2019.^4^ Due to severe air pollution and high rates of smoking as well as exposure to second-hand smoke, the prevalence of COPD in China continues to rise.^5^^,^^6^ Over 2012–2015, the COPD prevalence among residents aged 40 years and older was as high as 14%, which is about 1.7 times the prevalence observed over 2002–2004.^7^, ^8^, ^9^ At the same time, fewer than 3% of participants with spirometry-defined COPD in China were aware of their condition, according to a 2015 study.^8^ These numbers are especially astounding when compared to those for two other common non-communicable diseases: Recent studies showed that 45% of Chinese adults with hypertension and 37% of Chinese adults with diabetes were aware of their conditions.^10^^,^^11^

The grave consequences of COPD imply that effective interventions to reduce the COPD burden in China could substantially boost population health and well-being. Empirical data on COPD care cascade performance can help identify where patients are lost in the continuum of care (from screening to diagnosis to successful treatment) and serve as powerful evidence to guide future intervention designs.^12^^,^^13^ The term “care cascade” refers to a framework for measuring the progression of patients through discrete stages of care for a specified condition from need for care to successful treatment. The concept has a long history and has recently been applied to examine gaps in care for HIV, hypertension, tuberculosis, chronic hepatitis C, and diabetes.^14^, ^15^, ^16^, ^17^, ^18^, ^19^, ^20^, ^21^, ^22^, ^23^ Care cascades typically contain at least the following stages: screening, diagnosis, treatment, and control.^24^ Due to limited medical resources and insufficient attention from health policymakers to populations at risk for COPD, patients with COPD in China often do not receive early diagnosis and timely treatment, resulting in large unmet need for care. Lack of professional training among primary healthcare providers and lack of spirometry equipment further impede adequate and timely care delivery for patients with COPD in primary healthcare settings.^25^, ^26^, ^27^ Furthermore, unlike with hypertension and diabetes, COPD screening and management are not yet covered by basic public health services in China.^28^ Patients may therefore be excluded from early diagnosis and treatment because they have to pay out-of-pocket for those services. In addition, the knowledge level and awareness of COPD among the general public is relatively low,^29^ which could also contribute to delays in detection and treatment. Early treatment of patients with COPD who are asymptomatic or mildly symptomatic with pharmacotherapy interventions, such as tiotropium bromide, could significantly delay deterioration of their lung function and reduce the occurrence of acute exacerbations and mortality.^30^, ^31^, ^32^ Overall, early diagnosis and treatment can effectively improve quality of life of patients with COPD and help alleviate the diseases’ heavy population burden.^33^

To our knowledge, this is the first study to measure COPD care cascade performance in China. For this purpose, we used a nationwide dataset of patients with COPD—one of the largest such datasets on COPD care-seeking globally. The ‘Happy Breathing’ Programme from which this dataset was collected is the only national public-sector COPD programme in China. It aims to enroll patients with COPD among people seeking care in the Chinese health system. Our data thus represents the COPD cascade of care at enrolment into the ‘Happy Breathing’ Programme, i.e., before the enrolled patients have benefitted from the enhanced activities for COPD management in the ‘Happy Breathing’ Programme. For enrolment, the ‘Happy Breathing’ Programme employs comprehensive active and passive case finding, which is recommended by the 2023 Global Initiative for Chronic Obstructive Lung Disease (GOLD) guidelines, to identify COPD patients in the Chinese health system.^34^ We use patient data from the point of enrolment in the ‘Happy Breathing’ Programme to establish a baseline of care cascade outcomes among patients with COPD in the Chinese health system prior to programme intervention. Thus, our results serve two important purposes. First, they provide data on the need for improvements in the COPD care cascade in parts of China that are not yet benefitting from the national ‘Happy Breathing’ Programme. Second, they establish such a baseline for the ‘Happy Breathing’ Programme, against which programme performance can be evaluated in the future. It is important to note that this study took place in the general Chinese health system. Our data thus represent patients seeking care in the health system, rather than China's general population.

Our results are highly policy-relevant because they quantify the performance of the COPD care cascade in the Chinese health system using data from patients enrolling in the ‘Happy Breathing’ Programme. We aimed to determine where along the COPD care cascade patients are lost in the Chinese health system and how the patterns of loss vary across geographical regions and population groups. The findings of our study are likely to support improved COPD management in the Chinese health system and in the ‘Happy Breathing’ Programme itself. More broadly, our results may aid policymakers in designing and implementing effective, targeted interventions and health policies to alleviate the population health burden of COPD in China and globally.

## Methods

### Definition of the COPD cascade of care

COPD is characterized by persistent respiratory symptoms (including dyspnea, chronic cough, and sputum production) and airflow limitation. It is caused by airway or alveolar abnormalities and can be diagnosed through the presence of a post-bronchodilator FEV1/FVC <0.70.^34^, ^35^ Following a systematic search of titles and abstracts on PUBMED for work published between January 1, 1996, and July 25, 2022, with the search terms “chronic obstructive pulmonary disease” OR “COPD” and “care cascade” OR “unmet need” (with variations of the search terms “screened,” “aware,” “diagnosed,” “treated,” OR “controlled”), we found that researchers have generally focused on only one or two stages of the COPD care continuum, such as diagnosis and treatment, in any given study.^36^, ^37^, ^38^ Consequently, no published empirical research has yet systematically explored the performance of the healthcare system using the entire cascade of care for COPD. In addition, we did not find a single paper that describes the COPD care cascade across China. Ours is therefore the first empirical study of the COPD care cascade in China. We defined the five stages of the COPD care cascade in China as follows: the proportions of patients with COPD who have been (i) tested, (ii) diagnosed, (iii) treated, (iv) controlled with mild or no exacerbations in the past year, and (v) controlled with no exacerbations in the past year. We henceforth refer to stages (iv) and (v) as “Controlled 1” and “Controlled 2.” The COPD care cascade outcomes described in this paper reflect the statuses of patients with COPD at enrolment into the ‘Happy Breathing’ Programme.

Whether a participant had been tested was determined based on the following questions: “have you received pulmonary function test (PFT)?” and “have you ever received PFT in the past?” Whether a participant had been diagnosed was determined based on the question “have you ever been diagnosed with COPD?” Whether a patient had been treated was determined based on their record of previous medications. Patients were defined as treated if they had ever received a medication recommended by COPD treatment guidelines.^39^ In sensitivity analyses, we alternatively defined “treated” according to whether a patient had received either medication or a non-pharmaceutical intervention (e.g., influenza vaccination or pneumococcal vaccination, smoking cessation, long-term oxygen therapy, and domiciliary non-invasive ventilation), with the results of these analyses serving as comparisons for our main findings. We conducted this secondary analysis because both non-pharmaceutical interventions and medications are recommended by COPD treatment guidelines.^27^ Pulmonary rehabilitation is another comprehensive intervention to manage COPD,^40^ which includes exercise and education and can be successfully conduced in hospitals, communities, and homes.^41^ Unfortunately, information on whether patients have undergone pulmonary rehabilitation is not collected in the treatment history information in our data.

It is important to distinguish between severe exacerbations and mild exacerbations since severe exacerbations of COPD require emergency room visits or hospitalizations and may increase the risk of death for patients.^42^ Patients were defined as “Controlled 1” if they had experienced either mild or no exacerbations of COPD in the past 12 months and thus answered “no” to the question “have you been hospitalized in the past 12 months due to acute exacerbation?” Similarly, patients were defined as “Controlled 2” if they had experienced no exacerbations of COPD at all (including when not hospitalized) in the past 12 months and thus answered “no” to the question “have you experienced any acute exacerbations in the past 12 months?” Because the data on exacerbations was collected using patient self-report, we cannot independently confirm the accuracy of exacerbation severity, nor can we reliably identify the potential reasons for an exacerbation.

Since patients with an asthma-COPD overlap syndrome may be included in the sample, the initial “treated” proportion may be overestimated. To exclude patients with asthma, we applied the sequential conditional logic of the care cascade to our data. Specifically, (i) only patients tested for COPD were considered to have the chance to be diagnosed with COPD, (ii) only patients who had ever been diagnosed with COPD could receive effective treatment, and (iii) only patients who had ever been treated could be assessed for acute exacerbations. In summary, at each stage of the COPD care cascade, we only considered patients who had reached the previous stage.

### Data source

We collected individual-level data from all patients with COPD recorded in the national ‘Happy Breathing’ Programme in China, which was initiated on November 15, 2017. The ‘Happy Breathing’ Programme was designed by the National Center for Respiratory Diseases Clinic Research. The aims of this programme are to scale up early COPD detection, improve COPD treatment and management, and reduce readmission for patients with COPD. The programme established a new comprehensive management model for patients with COPD involving all healthcare institution levels (i.e., primary, secondary, and tertiary care), improved primary healthcare physicians' ability to care for patients with COPD, and boosted patients' adherence to therapy through multiple strategies (e.g., health education, self-management, and reminders).^35^ Baseline characteristics, prescriptions, follow-up visits, and referrals were recorded in the programme for each patient with COPD. As of April 22, 2021, the ‘Happy Breathing’ Programme had been launched in 29 Chinese regions, which were randomly selected for participation out of a potential pool of all Chinese regions (Appendix Figure S1). The regions of the ‘Happy Breathing’ Programme are China's administrative city-regions, including both urban and rural areas. The ‘Happy Breathing’ Programme was developed, designed and implemented with the support of domain experts in respiratory diseases as well as local health authorities. The implementation of this government-led, national programme for COPD management provided us with the opportunity to perform the first study of the performance of the COPD care cascade in the Chinese health system. Our data describe COPD care cascade outcomes for patients with COPD at the point of their enrolment into the national ‘Happy Breathing’ Programme and prior to the interventions that comprise the programme. These patients were identified for enrolment while accessing the Chinese health system for COPD or any other care. Our results are thus representative of people seeking care in the Chinese health system for any reason, and they can directly inform the design of novel interventions and policies to boost the performance of the COPD care cascade in the Chinese health system.

**Fig. 1:**
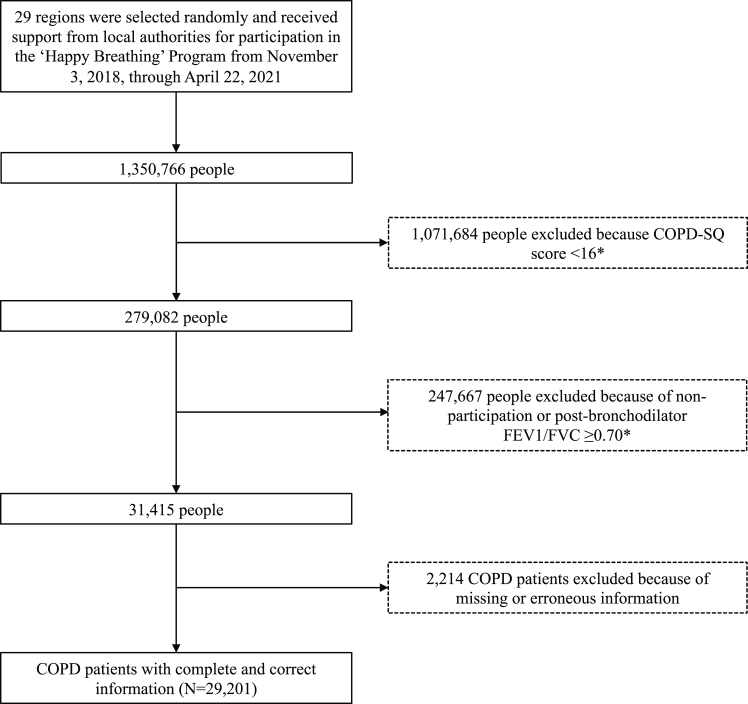
**Study population**. ∗This selection criterion was only applied to those people who did not have a clinically confirmed diagnosis of COPD prior to the start of the ‘Happy Breathing’ Programme.

The ‘Happy Breathing’ Programme uses comprehensive active and passive COPD case finding among people seeking care in the Chinese health system. For active case finding, the ‘Happy Breathing’ Programme asked all physicians in participating health care facilities to screen patients in their care for COPD using the COPD Screening Questionnaire (COPD-SQ).^43^ All patients in our study consented to participating in the ‘Happy Breathing’ Programme and to the use of their data for research and publication at the time of their enrollment. Patients whose COPD-SQ score was above 16, the recommended cut-off score for COPD***,*** and who consented to move on in the programme then received post-bronchodilator pulmonary function testing (Fig. 1). If patients scored below the cut-off of 0.7 for FEV_1_/FVC on the post-bronchodilator PFT, they were diagnosed as suffering from COPD, in accordance with the GOLD guidelines.^34^ For passive case finding, the physicians in the institutions participating in the ‘Happy Breathing’ Programme enrolled all consenting patients who had received a clinically confirmed diagnosis of COPD prior to the start of ‘Happy Breathing’ Programme.

Our results represent COPD care cascade outcomes of patients with COPD at the time they enrolled in the ‘Happy Breathing’ Programme. The programme intention was to reach everyone seeking healthcare in China for the initial COPD screening. Since our research utilizes programmatic data collected for clinical management rather than for scientific purposes, we lack quantitative information on the selection into the ‘Happy Breathing’ Programme. We thus do not know in how far the intention to reach everyone seeking care in the Chinese health system was not achieved by the programme, for instance, because patients did not receive the questionnaire or failed to fill it out. The starting point of our data are the 1,350,766 people in Fig. 1, i.e., the total number of people who either received the COPD-SQ as a screening process or who had a clinically confirmed diagnosis of COPD prior to the start of the ‘Happy Breathing’ Programme during the two-year enrollment phase of the ‘Happy Breathing’ Programme in all 29 regions where the programme was initiated.

Doctors and nurses participating in the ‘Happy Breathing’ Programme regularly receive online continuing medical education related to COPD diagnosis, treatment, and prevention of exacerbations (especially on how to measure lung function and follow up with patients). This online continuing medical education is led by well-known domestic experts in COPD. In addition, hospitals supervise the online learning progress of the medical staff, and the participating doctors are required to pass the COPD Knowledge Test. Moreover, since the doctors and nurses are educated and trained through an online platform, the implementation of the ‘Happy Breathing’ Programme in different regions is likely to be relatively similar across geographical contexts within China.

From November 3, 2018, to April 22, 2021, over one million people (1,350,766) were invited to participate in the ‘Happy Breathing’ Programme, and those who did not have a clinically confirmed diagnosis of COPD prior to the start of the ‘Happy Breathing’ Programme completed the COPD-SQ as part of the screening process. The COPD-SQ is a seven-item questionnaire to identify individuals at high risk for COPD, with respondents scoring between 0 and 11 on each item. Patients with a COPD-SQ score higher than 16 were defined as high risk. Using this cutoff, sensitivity, specificity, and correct classification rates were 60.6%, 85.2%, and 82.7%, respectively (Panel 1).^43^ In addition, depending on the particular patient and the clinical setting and care processes, post-bronchodilator PFT was often preceded by pre-bronchodilator PFT, and additional tests (such as chest X-ray and chest CT) were performed for differential diagnosis. The PFTs were performed by local healthcare workers who had received continued medical training through both online courses (taught by national Chinese COPD experts) and in-person field sessions (supervised by local pilot hospital COPD caretakers). Participants underwent PFTs three times during the same visit, and the highest measured ratio of forced expiratory volume in 1 s to forced vital capacity (FEV1/FVC) was recorded in the system. The measurement process and quality control for PFT results were both based on 2017 European Respiratory Society (ERS)/American Thoracic Society (ATS) standards.^44^ Specifically, we required participants to perform up to eight forced expiratory maneuvers until the forced vital capacity (FVC) and forced expiratory volume in 1 s (FEV1) were reproducible within 150 mL. All PFTs were performed with the participant in a seated position, wearing a nose clip, and using a disposable mouthpiece. Test results were stored in the spirometer and downloaded daily to a central computer system. An expert panel conducted regular quality controls of this measurement approach based on the ERS/ATS, and we excluded test results that were of poor quality; measurement was repeated in the case of poor-quality results. Three types of spirometers were used in the 'Happy Breathing' Programme, including ORANGER SP10BT, BreathHome A1, and e-LinkCare PF280, of which ORANGER SP10BT, a commonly used spirometer in the programme, was shown by a formal validation study to have sensitivity and specificity at 93.2% and 89.8%, respectively, when the Youden's index (sensitivity + specificity-1) was set at 0.830.^45^ Our final sample included 31,415 patients with COPD.

Panel 1 **Chronic obstructive pulmonary disease screening questionnaire.**

**Table:** 

Question number	Question	Answer questions
1	How old are you?	40–49, 50–59, 60–69, ≥70 years
2	Do you often cough?	Yes, No
3	Body mass index (kg/m^2^)	<18.5, 18.5–23.9, 24.0–27.9, ≥28.0 kg/m^2^
4	Smoking intensity (average number of packs of cigarettes smoked per day multiplied by smoking years)	Never smoked, 1–14.9, 15–29.9, ≥30 pack-year
5	Family history of respiratory disease	Yes, No
6	Exposure to biomass smoke from cooking fires	Yes, No
7	Which is the best description for your dyspnea?	(1) I don’t have a problem of breathlessness except during strenuous exercise. (2) I experienced shortness of breath when I was hurrying on flat ground or walking up a small slope. (3) I walk more slowly than people of the same age on flat ground due to breathlessness or have to stop for breath when I’m walking at my own pace on flat ground. (4) I have to stop for breath after walking on flat ground for about 100 meters or after a few minutes. (5) I am too breathless to leave the house or become breathless when I dress or undress.

Source: Zhou YM, Chen SY, Tian J, et al. Development and validation of a chronic obstructive pulmonary disease screening questionnaire in China. *International Journal of Tuberculosis and Lung Disease*. 2013.^43^

### Statistical analysis

We estimated the proportion of patients with COPD who reached each stage of the care cascade in every region. These proportion estimates were then plotted against the per-capita gross domestic product (GDP) from 2014 to 2019 in each region to explore the association between COPD care cascade outcomes and regional economic development level. We collected per-capita GDP data from the National Bureau of Statistics of China, and we calculated the mean annual per-capita GDP over the six-year period from 2014 to 2019 for each of the 29 regions (in constant 2017 international dollars, as estimated by the World Bank).^46^^,^^47^ We measured the association between care cascade outcomes and regional economic development level using beta regression on the proportion estimates against log-transformed six-year per-capita GDP weighted by sample size in each region. We used log-transformed per-capita GDP because of skewness in the untransformed distribution of this variable. In addition to examining the association with economic development, we also plotted COPD care cascade outcomes against the pollution level in each region. For our pollution indicator, we used the six-year mean of PM2.5 (defined as fine particles, 2.5 μm or less in size, in the ambient air), for the period 2014–2019 and for each region where the ‘Happy Breathing’ Programme was implemented.^48^ We examined the relationship between COPD and PM2.5 because prior literature has suggested that PM2.5 is a risk factor for COPD.^49^^,^^50^

We further regressed the binary outcomes of whether the patients had reached each cascade stage on sex, age group (54 years or younger, 55–64, 65 or older), education, region, body mass index (BMI) group (<18.5 kg/m^2^ as underweight, 18.5–24.0 kg/m^2^ as healthy weight, 24.0–28.0 kg/m^2^ as overweight, and above 28.0 kg/m^2^ as obese), and smoking status. We performed multilevel regression with regional population size applied as a weight for the GDP analysis; specifically, we applied multilevel modeling with a region-level random intercept and per-capita GDP as region-level independent variables, using population size as a weight. We further added two regional-level independent variables to broadly capture the availability of resources in the Chinese health system: (i) the population density of healthcare facilities (to capture the structural availability of healthcare) and (ii) the population density of physicians (to capture the availability of health services). We collected data on the total number of healthcare facilities and physicians in each region of China from the official websites of the province-level statistical bureaus for the years 2014–2019. This data was then divided by the permanent population of each region for each year to obtain the annual healthcare facility density per 10,000 people and the annual physician density per 10,000 people for each of the six years. To check the robustness of our results, we further (i) fitted univariable and multivariable modified Poisson regressions for binary outcomes on both the full sample and a sample stratified based on per-capita GDP.^51^ We additionally applied a stratified analysis based on modified Poisson models according to the results from the modified Medical Research Council (mMRC) dyspnea scale, which ranges from 0 to 4 (with higher scores corresponding to higher severity of dyspnea), to explore how our variables affect COPD care cascade outcomes in patients with different severity of respiratory symptoms. In the modified Poisson models, we estimated generalized linear regression models with a Poisson distribution, the log link function, and robust standard errors, i.e., modified Poisson regression for binary data.^51^ Standard errors were adjusted for clustering at the regional level. Finally, we performed interaction analyses to test the interaction effects between different independent variables in our regression models in multilevel regressions. All of these additional regression results are shown in the Appendix (Appendix Tables S1–S6). We tested for multicollinearity using the variance inflation factor (VIF) (Appendix Tables S7 and S8).^52^ Individuals with missing values for independent variables were removed from the regression analysis; we also had to remove individuals in regions with a sample size less than 100 because our regression models did not converge when these observations were included. Our analysis is a secondary data analysis and was not registered and pre-specified.

### Ethics and dissemination

Ethics approval was granted by China–Japan Friendship Hospital (approval number 2019-41-k29). This study was conducted in accordance with the ethical principles of the Declaration of Helsinki. All participants provided written informed consent.

### Role of the funding source

Funding sources had no role in study design, data collection/analyses/interpretation, manuscript preparation, or submission. All authors had full access to all of the study data and approved the final version of the manuscript.

## Results

### Descriptive statistics

A total of 29,201 patients with complete information were included in the analysis of the COPD care cascade. Females accounted for 35.2% of the final sample, and the age range was 20–100 years old, with a median age of 67 years (Table 1).

**Table 1:** Sample characteristics.

Region	First year of data collection	Sample size	Median age (IQR), years	Age range, years	Female, %	Per-capita GDP (2014–2019), international $	Average PM 2.5 (μg/m^3^)
Ankang	2018	514	61 (52–69)	26–97	21.4%	5246.75	29.64
Baiyin	2018	1822	64 (54–72)	24–92	40.2%	4026.98	37.52
Beijing	2018	288	67 (61–73)	31–91	30.9%	18952.88	50.90
Cangzhou	2018	373	67 (61–72)	21–90	51.2%	6988.2	68.74
Chifeng	2018	1492	66 (60–72)	24–89	42.9%	5041.08	23.62
Chongqing	2020	471	72 (65–78)	25–91	23.1%	9096.89	37.53
Daqing	2019	622	72 (67–77)	26–92	50.4%	16148.2	33.95
Datong	2019	2200	65 (58–71)	35–93	34.7%	4952.3	33.87
Deyang	2020	101	72 (66–78)	43–94	32.7%	8063.7	41.73
Ganzhou	2020	22	67.5 (60–77)	48–92	22.7%	4308.05	31.32
Guilin	2020	34	67 (62–75)	54–82	26.5%	5981.6	37.49
Hengyang	2020	8	62 (53–68)	44–85	0.0%	5914.2	44.25
Huaihua	2019	3727	69 (61–76)	21–99	29.7%	4249.12	36.33
Huangshan	2019	756	70 (64–76)	37–98	25.5%	6717.2	30.41
Hulunbeier	2019	819	60 (51–68)	26–91	40.1%	8274.23	17.85
Huzhou	2019	491	65 (56–72)	22–91	35.9%	12657.93	42.59
Jinan	2021	5	65 (51–65)	44–70	20.0%	14266.38	62.85
Jinzhou	2019	2	65.5 (51–80)	51–80	50.0%	5776.46	44.15
Kunming	2020	78	70.5 (65–79)	24–94	16.7%	10,555	24.79
Luoyang	2020	199	67 (63–72)	29–86	45.7%	9028.11	44.76
Tianjin	2019	1423	66 (60–72)	20–99	35.3%	16456.74	64.96
Tonghua	2019	74	65 (57–72)	40–90	37.8%	6094.83	35.95
Weifang	2018	973	67 (61–72)	21–92	29.9%	8769.43	53.96
Wuhan	2020	382	69 (65–73)	23–90	38.2%	17896.63	54.00
Xiangtan	2018	1618	67 (59–73)	20–93	24.0%	10175.23	49.05
Yangzhou	2020	54	69 (63–72)	54–80	24.1%	15845.28	51.13
Yinchuan	2019	2196	67 (59–76)	20–95	42.0%	11497.93	37.01
Zhoukou	2018	1050	66 (57–74)	20–96	29.5%	4193.13	60.52
Zunyi	2018	7407	66 (55–73)	20–100	37.2%	6292.95	34.46
Overall	2018	29,201	67 (57–73)	20–100	35.2%	NA	NA

### COPD care cascade outcomes

**Fig. 2:**
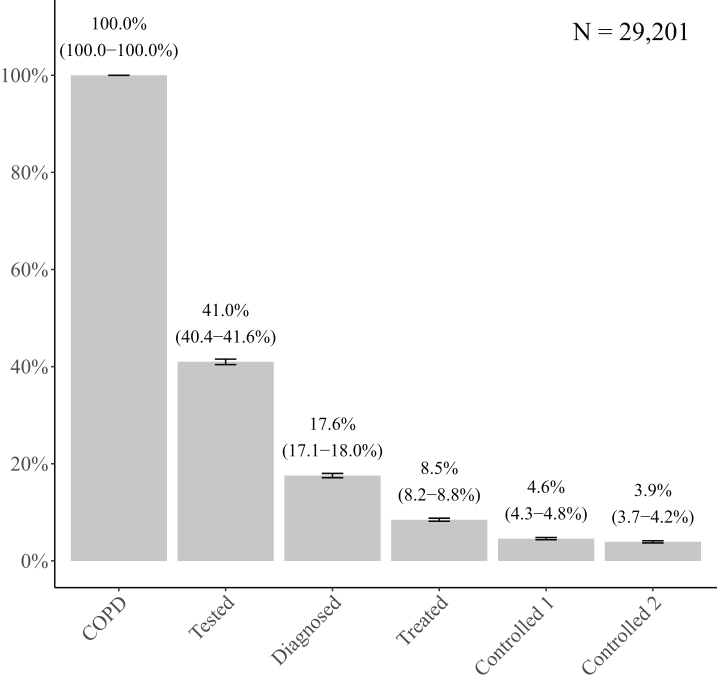
**COPD care cascade outcomes in China prior to intervention under the ‘Happy Breathing’ Programme**. Note: Data are percentages with 95% CIs. Controlled 1 = Controlled (mild or no exacerbations), Controlled 2 = Controlled (no exacerbations).

At enrolment into the ‘Happy Breathing’ Programme, of the total of 29,201 patients 41.0% (95% confidence interval [CI]: 40.4–41.6%) had reached the testing stage of the COPD care cascade, 17.6% (95% CI: 17.1–18.0%) had reached the diagnosis stage, and 8.5% (95% CI: 8.2–8.8%) had been treated for COPD. Less than five percent of patients had achieved control of their COPD within one year prior to enrolment. Specifically, 4.6% (95% CI: 4.3–4.8%) of patients had mild or no exacerbations in the prior year, and 3.9% (95% CI: 3.7–4.2%) of patients had suffered no exacerbations in the prior year (Fig. 2). When we defined “treated” as having received either medication or a non-pharmaceutical intervention according to COPD treatment guidelines, 12.7% (95% CI: 12.3–13.0%) had been treated for COPD (Appendix Figure S2). Patients who skipped at least one step in the cascade were excluded from our primary analysis. When we included these patients in our analytical sample group, the proportion of patients with COPD who ever tested was 39.3% (95% CI: 38.8–39.9%), the proportion of patients who ever received diagnosis was 20.6% (95% CI: 20.1–21.0%), and the proportion of patients who ever received treatment was 16.5% (95% CI: 16.1–16.9%).

**Fig. 3:**
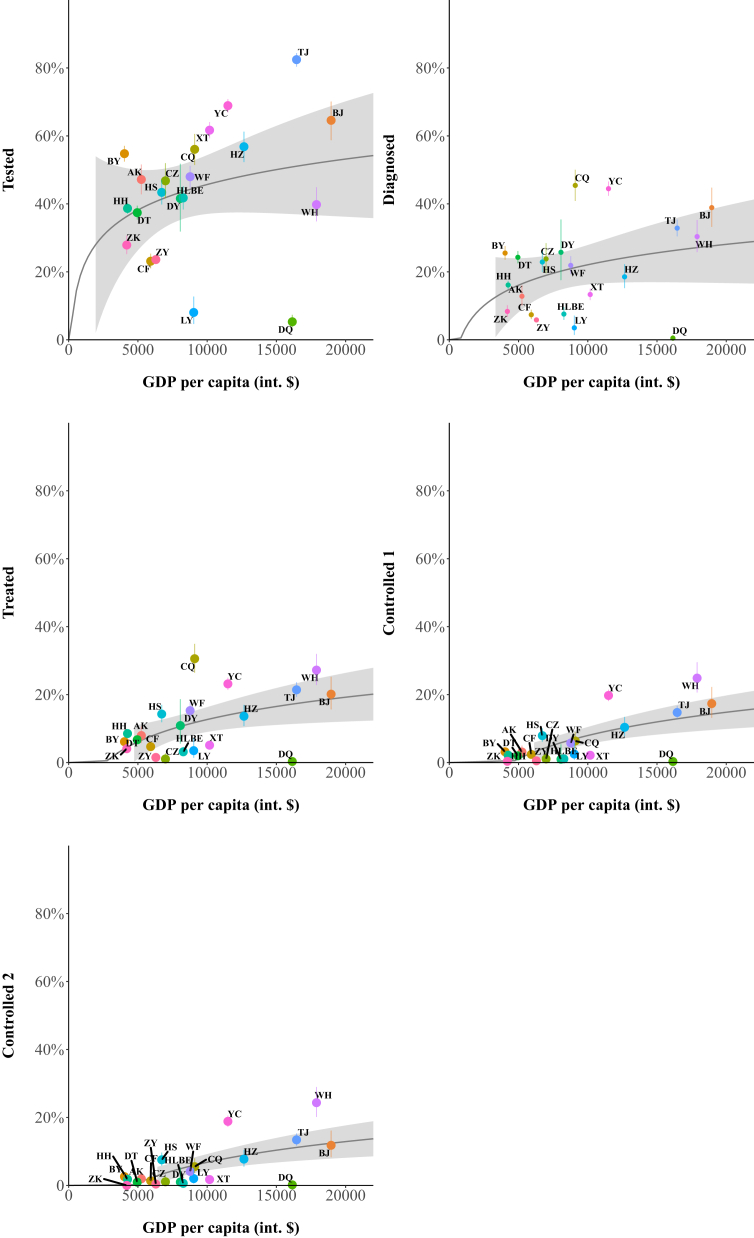
**COPD care cascade outcomes by per-capita GDP**. Note: “GDP per capita (int. $)” denotes the mean annual per-capita GDP over the six-year period from 2014 to 2019 for each of the 29 regions (in constant 2017 international dollars, as estimated by the World Bank). Regions with sample size under 100 were not included in this analysis. The grey boundary shows the point–wise 95% prediction interval, and the vertical bars are 95% CIs around point estimates. The p-values for the coefficients of the Beta regressions of all cascade stages onto per-capita GDP are <0.001. AK, Ankang; BJ, Beijing; BY, Baiyin; CF, Chifeng; CQ, Chongqing; CZ, Cangzhou; DQ, Daqing; DT, Datong; DY, Deyang; GDP, Gross Domestic Product; HH, Huaihua; HLBE, Hulunbeier; HS, Huangshan; HZ, Huzhou; LY, Luoyang; TJ, Tianjin; WF, Weifang; WH, Wuhan; XT, Xiangtan; YC, Yinchuan; ZK, Zhoukou; ZY, Zunyi; Controlled 1, Controlled (mild or no exacerbations); Controlled 2, Controlled (no exacerbations).

Regional per-capita GDP was positively associated with the proportion of patients reaching each cascade stage (Appendix Table S9). Among all regions, Yinchuan performed substantially better than predicted on all measured cascade outcomes based on its recent six-year per-capita GDP, while Daqing and Luoyang performed significantly worse than predicted (Fig. 3). These results were robust in simple linear regression plots of both the full sample and each age subgroup (Appendix Figures S3–S6). Yinchuan also performed substantially better than predicted on all measured cascade stages, when the predictions were based on six-year average PM2.5, while Zhoukou, Daqing, and Luoyang performed significantly worse than predicted (Appendix Figure S7).

### Disparities analysis

Patients who were older were more likely to reach each stage of the COPD care cascade and those who were male and underweight were more likely to reach the stage of tested (Table 2). Men were more likely to be tested when the degree of the mMRC Dyspnea scale was less than four (Appendix Table S5). Additionally, compared to people with healthy weight, being underweight was associated with a higher probability of reaching the stages of tested, diagnosed. Moreover, compared to patients in rural townships, patients living in urban areas were more likely to reach each stage of the COPD cascade except for diagnosed. Our multilevel regression results also showed that regions with higher per-capita GDP performed better at each stage of the COPD care cascade. In separate, nested multilevel regressions, we added two variables that capture health system resource availability—the regional availabilities of healthcare facilities and doctors—to test whether these explain the association of per-capita GDP, which captures general resources, with the outcomes along the COPD care cascade. The associations of the variables capturing health system resource availability were small and not statistically significant and their addition only slightly reduced the size of the association between per-capita GDP and the cascade outcomes (Table 2).

**Table 2:** Multilevel regressions of each cascade stage on individual-level and regional-level independent variables.

Variable	Tested				Diagnosed				Treated				Controlled 1				Controlled 2			
	RRR (95% CI)	p-value	RRR (95% CI)	p-value	RRR (95% CI)	p-value	RRR (95% CI)	p-value	RRR (95% CI)	p-value	RRR (95% CI)	p-value	RRR (95% CI)	p-value	RRR (95% CI)	p-value	RRR (95% CI)	p-value	RRR (95% CI)	p-value
**Sex**																				
Female	1 (ref)	–	1 (ref)	–	1 (ref)	–	1 (ref)	–	1 (ref)	–	1 (ref)	–	1 (ref)	–	1 (ref)	–	1 (ref)	–	1 (ref)	–
Male	1.19 (1.07, 1.32)	0.00	1.19 (1.07, 1.32)	0.00	1.18 (0.96, 1.46)	0.12	1.18 (0.95, 1.46)	0.13	1.38 (1.00, 1.91)	0.05	1.38 (1.00, 1.91)	0.05	1.26 (0.82, 1.93)	0.29	1.26 (0.82, 1.93)	0.30	1.25 (0.88, 1.78)	0.21	1.25 (0.88, 1.79)	0.21
**Age, years**																				
54 and below	1 (ref)	–	1 (ref)	–	1 (ref)	–	1 (ref)	–	1 (ref)	–	1 (ref)	–	1 (ref)	–	1 (ref)	–	1 (ref)	–	1 (ref)	–
55–64	1.20 (1.12, 1.28)	<0.001	1.20 (1.12, 1.28)	<0.001	2.22 (1.85, 2.67)	<0.001	2.22 (1.85, 2.68)	<0.001	2.20 (1.44, 3.37)	<0.001	2.20 (1.44, 3.38)	<0.001	1.95 (1.38, 2.75)	<0.001	1.95 (1.38, 2.75)	<0.001	1.80 (1.25, 2.59)	0.00	1.80 (1.25, 2.59)	0.00
65 and above	1.25 (1.12, 1.40)	<0.001	1.25 (1.12, 1.40)	<0.001	2.63 (2.07, 3.34)	<0.001	2.63 (2.07, 3.33)	<0.001	2.57 (1.65, 3.99)	<0.001	2.57 (1.65, 4.00)	<0.001	1.83 (1.29, 2.59)	0.00	1.83 (1.29, 2.59)	0.00	1.65 (1.22, 2.22)	0.00	1.65 (1.22, 2.23)	0.00
**Education**																				
Primary education and below	1 (ref)	–	1 (ref)	–	1 (ref)	–	1 (ref)	–	1 (ref)	–	1 (ref)	–	1 (ref)	–	1 (ref)	–	1 (ref)	–	1 (ref)	–
Secondary education	1.01 (0.90, 1.14)	0.84	1.01 (0.90, 1.14)	0.84	1.08 (0.92, 1.27)	0.37	1.08 (0.92, 1.27)	0.37	1.26 (1.12, 1.42)	<0.001	1.26 (1.12, 1.42)	<0.001	1.52 (1.34, 1.72)	<0.001	1.52 (1.34, 1.72)	<0.001	1.73 (1.44, 2.09)	<0.001	1.73 (1.43, 2.09)	<0.001
College education	1.06 (0.84, 1.34)	0.64	1.06 (0.84, 1.34)	0.64	1.07 (0.73, 1.58)	0.73	1.07 (0.73, 1.58)	0.73	1.32 (0.97, 1.78)	0.08	1.31 (0.97, 1.78)	0.08	1.22 (0.94, 1.59)	0.13	1.22 (0.94, 1.59)	0.14	1.16 (0.95, 1.42)	0.15	1.16 (0.95, 1.41)	0.15
**Body-mass index group**																				
Underweight	1.14 (1.04, 1.24)	0.00	1.14 (1.04, 1.24)	0.00	1.34 (1.17, 1.54)	<0.001	1.35 (1.17, 1.54)	<0.001	1.09 (0.94, 1.26)	0.25	1.09 (0.94, 1.26)	0.26	0.88 (0.62, 1.25)	0.49	0.88 (0.62, 1.25)	0.49	0.84 (0.62, 1.13)	0.25	0.84 (0.62, 1.13)	0.26
Healthy weight	1 (ref)	–	1 (ref)	–	1 (ref)	–	1 (ref)	–	1 (ref)	–	1 (ref)	–	1 (r4ef)	–	1 (r4ef)	–	1 (ref)	–	1 (ref)	–
Overweight	0.94 (0.88, 1.01)	0.10	0.94 (0.88, 1.01)	0.10	0.98 (0.87, 1.10)	0.72	0.98 (0.87, 1.10)	0.72	0.92 (0.77, 1.09)	0.33	0.92 (0.77, 1.09)	0.34	0.84 (0.67, 1.06)	0.14	0.84 (0.67, 1.05)	0.14	0.81 (0.65, 1.01)	0.07	0.81 (0.65, 1.01)	0.06
Obese	0.98 (0.89, 1.08)	0.70	0.98 (0.89, 1.08)	0.70	0.97 (0.82, 1.15)	0.75	0.97 (0.82, 1.15)	0.75	0.88 (0.67, 1.15)	0.35	0.88 (0.67, 1.16)	0.36	0.74 (0.58, 0.96)	0.02	0.74 (0.58, 0.95)	0.02	0.77 (0.62, 0.96)	0.02	0.77 (0.62, 0.96)	0.02
**Tobacco smoking**																				
Never smoked	1 (ref)	–	1 (ref)	–	1 (ref)	–	1 (ref)	–	1 (ref)	–	1 (ref)	–	1 (ref)	–	1 (ref)	–	1 (ref)	–	1 (ref)	–
Ever smoked	0.80 (0.58, 1.09)	0.15	0.80 (0.58, 1.09)	0.15	1.06 (0.82, 1.35)	0.67	1.06 (0.83, 1.36)	0.66	1.04 (0.78, 1.39)	0.77	1.05 (0.78, 1.41)	0.75	1.18 (0.76, 1.82)	0.45	1.18 (0.77, 1.83)	0.46	1.26 (0.81, 1.95)	0.30	1.26 (0.81, 1.96)	0.30
**Urbanicity**																				
Rural townships	1 (ref)	–	1 (ref)	–	1 (ref)	–	1 (ref)	–	1 (ref)	–	1 (ref)	–	1 (ref)	–	1 (ref)	–	1 (ref)	–	1 (ref)	–
Urban areas	1.31 (1.12, 1.53)	0.00	1.31 (1.12, 1.53)	0.00	1.25 (0.97, 1.63)	0.09	1.25 (0.97, 1.63)	0.09	1.43 (1.13, 1.80)	0.00	1.43 (1.14, 1.80)	0.00	1.60 (1.37, 1.87)	<0.001	1.60 (1.36, 1.89)	<0.001	1.60 (1.26, 2.04)	<0.001	1.60 (1.25, 2.06)	<0.001
**GDP per capita**	1.42 (1.06, 1.92)	0.02	1.39 (0.77, 2.53)	0.27	1.84 (1.16, 2.93)	0.01	1.39 (0.81, 2.39)	0.24	2.22 (1.39, 3.53)	0.00	2.66 (0.92, 2.99)	0.10	4.99 (3.02, 8.23)	<0.001	3.68 (1.89, 7.16)	<0.001	6.22 (2.72, 14.27)	<0.001	5.20 (1.93, 14.01)	<0.001
**Density of healthcare facilities**			1.00 (0.97, 1.02)	0.77			0.97 (0.93, 1.01)	0.17			0.97 (0.89, 1.05)	0.40			1.00 (0.95, 1.04)	0.77			0.99 (0.94, 11.05)	0.81
**Density of physicians**			1.00 (0.96, 1.04)	0.84			1.02 (0.99 1.06)	0.19			1.02 (0.97, 1.08)	0.45			1.03 (0.98, 1.09)	0.21			1.02 (0.95, 1.09)	0.64
**Log pseudo-likelihood**	−9969423.5		−9948263.7		−6381863.2		−6365505.2		−4188882.8		−4,178,007		−2305539.8		−2304394.3		−1962288.2		−1961670.6	

Note: COPD, chronic obstructive pulmonary disease; Controlled 1, Controlled (mild or no exacerbations); Controlled 2, Controlled (no exacerbations). Regions with sample size under 100 were not included in this analysis. N = 23,027.

In analyses that included only individual-level independent variables, the relationships between the independent variables and the COPD care cascade outcomes were similar to the results from the multilevel analysis presented above except for female sex and high education attainment, which demonstrated more significant associations with larger losses at each stage of the COPD care cascade (Appendix Table S1). Furthermore, after stratifying the participating regions into three groups based on per-capita GDP, we found that in low- and middle-GDP regions, patients who lived in urban areas had a higher probability of being tested than patients residing in rural areas (Appendix Tables S2–S4). We also found two important interaction effects. First, the positive associations between the individual-level education variable and being tested and diagnosed decreased with increasing regional per-capita GDP. Second, the positive association between the individual-level urbanicity variable and being tested and diagnosed decreased with increasing regional per-capita GDP (Appendix Table S6). We found that the VIFs for regression models that included individual-level independent variables were lower than the conventional cutoff of ten for high multicollinearity, with the exception of the indicator variable for the region of Zunyi (for which the VIF was 10.87) (Appendix Table S7). After excluding the observations from Zunyi from the analysis, the regression results remained essentially the same (Appendix Table S10), while all VIFs remained below ten (Appendix Table S8).

### Stratification analysis

**Fig. 4:**
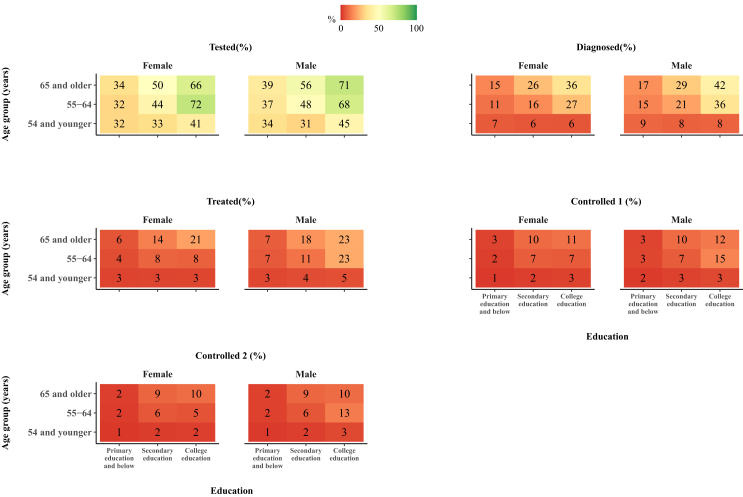
**Proportion of patients with COPD reaching each cascade stage, stratified by sex, age group, and education**. Note: Controlled 1, Controlled (mild or no exacerbations); Controlled 2, Controlled (no exacerbations). Primary education and below refers to having received some primary education or having completed primary school. Secondary education refers to having completed secondary school (including junior and senior high school or technical secondary school). College education refers to having received some type of tertiary education.

We evaluated the COPD care cascade outcomes in population groups stratified by sex, age group, and education (Fig. 4) and found that the proportion of patients achieving the treated stage was less than 25% in all age and education group combinations. In addition, men had a higher probability of reaching each cascade stage than women in almost all age and education group combinations.

**Fig. 5:**
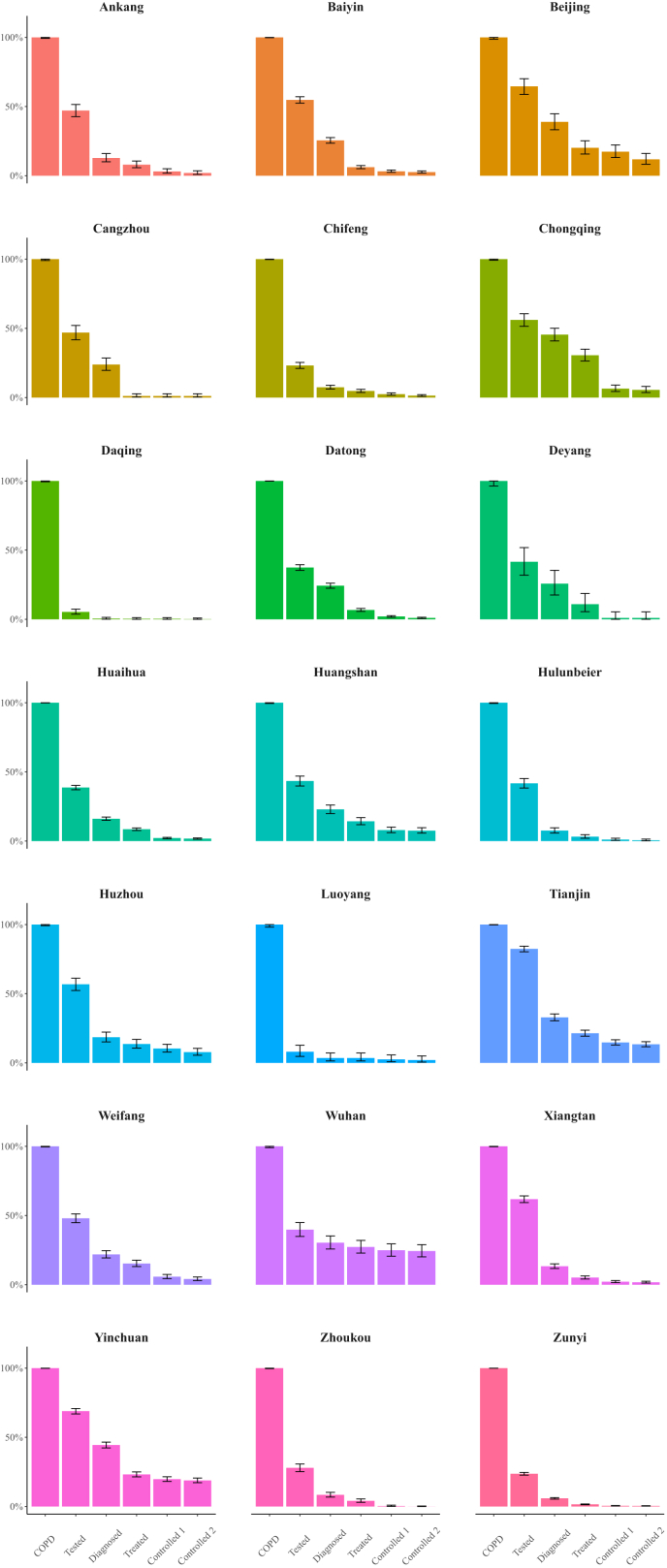
**COPD care cascade outcomes across the regions participating in the ‘Happy Breathing’ Programme in China**. Note: From left to right, the columns in each sub-figure show: COPD, Tested, Diagnosed, Treated, Controlled 1, and Controlled 2. Controlled 1, Controlled (mild or no exacerbations); Controlled 2, Controlled (no exacerbations). This figure only shows the COPD care cascade outcomes in regions with a sample size larger than 100.

COPD care cascade outcomes varied widely across regions (Fig. 5). Patients living in Beijing, Wuhan, and Yinchuan achieved comparatively high coverage across all stages of the COPD care cascade, while patients living in Daqing and Luoyang achieved comparatively low coverage. The care cascade outcomes disaggregated by age group and region are shown in the Appendix (Appendix Figures S8 and S9). The outcomes for each age group were similar to the outcomes for the overall sample, but patients in the older age groups were more likely to reach the first three stages of the cascade.

## Discussion

Our study used the data on the COPD care cascade in China that is routinely collected at enrolment into the ‘Happy Breathing’ Programme, a national programme that is intended to boost the effectiveness of COPD care in China. Our findings offer valuable insights for both patients with COPD who are enrolled in the ‘Happy Breathing’ Programme and patients with COPD who do not yet have access to the programme. With regards to patients in the program, this study provides a baseline against which to benchmark future COPD outcomes, following the specific health management interventions in the programme. Our findings also constitute a plausible approximation of care cascade outcomes for patients who seek care in the Chinese health system and who do not (yet) have access to the ‘Happy Breathing’ Programme, which is the majority of COPD patients in China.

We found that only four in ten patients with COPD in this large sample from 29 regions of China had ever been tested for the disease, and fewer than two in ten had ever received a formal clinical diagnosis. The absolute losses in later stages of the care cascade were small compared to the losses in these first two stages; however, the relative losses in later stages remained large. Overall, in our primary analysis, less than 5% of people living with COPD who sought care for any reason in the Chinese health system were successfully treated for their COPD. The proportion of patients reaching the treated stage was about twice as high when we included patients who had skipped at least one stage in the COPD care cascade. A reason for this finding may be that physicians did not see the need to follow the tested and diagnostic stages of the COPD care cascade among patients who were already in their care for COPD at the time when they newly enrolled in the ‘Happy Breathing’ Programme.

This study observed disparities in care cascade outcomes across geographic regions and population groups. Patients in Beijing, Wuhan, and Yinchuan were more likely to reach all stages of the COPD care cascade than patients living in Daqing and Luoyang. The observed high geographic variability of COPD care indicates that substantial improvements in national care cascade outcomes could be possible, if outcomes in the currently low-performing regions were brought closer to those in the currently high-performing regions. In addition, we found higher performance in each step of the COPD care cascade for regions with higher per-capita GDP. However, indicators of the availability of healthcare facilities and the availability of healthcare services were not significantly associated with COPD care cascade outcomes. Thus, it is unlikely that these health system availability indicators transmit the effect of per-capita GDP on COPD cascade outcomes. Instead, potential pathways through which per-capita GDP impacts COPD care cascade outcomes may be factors relating to healthcare quality rather than quantity: Wealthier regions will be able to build better healthcare facilities and attract better physicians and other health workers, which may boost COPD care cascade outcome. Future research should try to address the challenges of measuring healthcare quality in order to further explain the association between GDP and care cascade outcomes.

We further found that patients with COPD who were younger, and from rural townships were less likely to reach each stage of the COPD care cascade and those who were female were less likely to reach the step of tested in COPD care cascade. These findings are in line with previous research. According to one study, men tend to know more about COPD and its risk factors than women.^53^ Patients in older age groups have a higher probability of developing severe clinical symptoms compared to younger patients since lung function declines even during the long asymptomatic phase, explaining why young patients with COPD are less likely to be aware of their condition. Patients may only seek medical care when they are in an advanced stage of illness or when they have experienced an acute exacerbation.^54^ Low levels of education generally imply low health-specific knowledge and low ability to successfully negotiate access to healthcare.^55^ Similarly, rural-to-urban migrants in China tend to suffer disadvantages in knowledge and formal entitlements to access urban health care, explaining why those from rural townships in the participating regions were less likely to reach advanced stages in the COPD care cascade.^56^

With respect to risk factors, we found that those who were underweight had a higher probability of reaching the cascade stages of tested and diagnosed. Underweight is a highly salient clinical sign and may thus provide additional independent motivation for both patient and physician to ensure that major chronic conditions are treated. It may also simply reflect the fact that underweight increases with severity of COPD and patients with more severe diseases are more likely to receive treatment.^57^, ^58^, ^59^ Furthermore, given that studies have consistently indicated that smoking is a major risk factor for COPD, it is worrying to see that smokers did not have a higher probability of reaching each stage of the COPD care cascade than non-smokers.^8^^,^^60^, ^61^, ^62^, ^63^, ^64^ The univariable analysis showed a positive association between ever smoking and COPD control, but this association disappeared when we controlled for sex, age, education, BMI, and urbanicity in multivariable regression. The similarity in care cascade outcomes for smokers and non-smokers may indicate that the underlying causes of COPD other than smoking, such as ambient and indoor air pollution, second-hand smoke, and occupational exposures, may be as difficult to reduce as smoking. This finding suggests that both smokers and non-smokers can likely substantially benefit from prevention programmes to reduce harmful continued exposures to the underlying causes of COPD.

COPD care cascade outcomes in China fall short of those for other chronic conditions such as hypertension and diabetes. Among hypertensive patients, 45% had previously been diagnosed, 30% were taking antihypertensive medications, and 7% had achieved control, and among diabetic patients, 37%, 33%, and 17% had reached each of these stages, respectively.^11^^,^^65^ However, these chronic disease care cascades clearly also need to be substantially boosted, to ensure that patients benefit full from available treatment and management. Interventions to boost the COPD cascade of care should be policy priority in China, potentially alongside interventions to improve the care cascade for other chronic conditions. A reason for the failure to ensure high levels of care cascade attainment for COPD may be that COPD is not included in the essential public-sector health services in China (while hypertension and diabetes are included).^66^ The findings from our study strongly suggest that major efforts are needed to accelerate the inclusion of COPD in the essential public-sector health services and that policymakers and healthcare workers should find ways to facilitate early detection, diagnosis, and treatment to reduce the substantial burden of COPD.

Information on care cascades for other respiratory conditions and diseases would also be important to judge the performance and quality of routine care both in China and globally. Currently, care cascade outcomes are only available for tuberculosis (TB). For instance, in India, 68% of patients had accessed TB tests, 57% were diagnosed with TB, 50% were treated, 43% had treatment success, and 37% experienced recurrence after survival. South Africa performed better with respect to TB care cascade outcomes, with 95% of patients accessing TB tests, 82% receiving TB diagnosis, 70% being notified and treated, and 53% achieving treatment success.^24^ An important research gap in respiratory medicine is the lack of information on cascade of care outcomes for asthma. It is possible that the asthma care cascade is very different from the COPD care cascade, because people suffering from asthma tend to be identified and treated in substantially younger ages than people suffering from COPD. We hope that future studies will start to close this research gap.

By examining COPD care cascade outcomes relative to regional per-capita GDP, our analysis identified the regions that stood out with desirable performance, such as Yinchuan. This result can aid policymakers in identifying the types of health policies and health system infrastructure features that account for regional success. For instance, Yinchuan has used digital health services since late 2016 to remotely manage patients with non-communicable diseases.^67^ Although Yinchuan is relatively underdeveloped and residents generally have less access to medical resources than in other regions, the availability of online diagnosis and treatment has greatly expanded access to high-quality healthcare. Investing in digital health has greatly improved the efficiency with which health system resources are allocated, promoted equity, and had a significant positive impact on population health. Since the online diagnosis and treatment model was first implemented, the Yinchuan government has introduced a series of additional relevant policy measures, including supervision, management, and insurance policies supporting digital health.^68^

The results of this study have several additional important implications for health policy in China. First, implementing major population health interventions to boost screening, testing, and diagnosis of COPD in the Chinese health system will be critical to reduce the national burden of COPD. The vast majority of people living with COPD will not be able to receive proper treatment unless massive improvements are made with respect to COPD screening and care. We find major losses at the testing and diagnosis stages of the COPD care cascade in the Chinese health system. Major improvements in these stages could be achieved by boosting COPD screening programmes both in health facilities, where this study took place, and in community and household settings, where populations who are even less likely to be tested for COPD could be reached.

Second, our study suggests several risk factors for losses in the COPD care cascade. These risk factors could be useful for future intervention design to improve the COPD care cascade in the Chinese health system. First, socio-demographic and structural risk factors could be used for intervention targeting and tailoring to populations that are particularly likely to fail to progress along the care cascade. These factors include female sex, young age, low educational attainment, and rural residence. A simple intervention could, for instance, raise physicians’ awareness that women are more likely to be lost to follow-up in COPD care. Second, behavioural risk factors are of course directly amenable to change and should thus be addressed during COPD care. In particular, we find that tobacco smoking is positively associated with losses along the COPD care cascade. Smoking cessation programs would thus likely not only reduce further progression of COPD but also improve COPD care outcomes by ensuring that patients progress along the cascade of care. Previous evidence has also shown that patient awareness, health literacy, and access to healthcare positively affect the management of chronic diseases like COPD.^8^^,^^69^, ^70^, ^71^

Third, the government needs to improve access to healthcare for patients with COPD, especially in rural townships and underperforming regions, such as Daqing and Luoyang. Improved access will help reduce inequities in COPD medical resource allocation. In addition, providing financial incentives for COPD screening and care may improve cascade outcomes, especially for low-income patients. Financial incentives have been successfully used to boost screening and care for other chronic diseases in China, such as hypertension and diabetes.^72^, ^73^, ^74^ Policymakers also need to pay increased attention to medication availability. In China, the supply of COPD medication in primary hospitals is insufficient, which may affect patient adherence to standardized treatment. Increasing the medication supply at the primary care level may therefore improve COPD control, especially among patients in rural areas. At the same time, there is a significant regional disparity in the household economic burden associated with taking medication for COPD in China. In more-developed cities such as Beijing, patients are more likely to be reimbursed for COPD medication than in less-developed cities and regions of China (e.g., provinces in the northwest), where patients typically bear large proportions of treatment costs.

Fourth, most patients with a clinical diagnosis of COPD will fail to benefit from treatment unless the clinic- and hospital-based stages of the care cascade are improved. China could substantially improve the quality of COPD management in primary healthcare institutions. Specifically, China could provide professional training, finance the purchase of spirometers, and implement innovative interventions such as digital health, mobile health, and telemedicine.^75^, ^76^, ^77^ China could further enhance the continuity of the COPD care cascade by integrating health promotion, prevention, diagnosis, control, treatment, and recovery into COPD management. In addition, the country could establish a two-way referral path (from primary healthcare institutions to secondary and tertiary hospitals and vice versa) for strategic triage of COPD care to reduce the loss of patients at each care cascade stage, thereby comprehensively addressing unmet need.

Fifth, care continuity should be emphasized in updated versions of clinical guidelines for COPD care in China. The current “Guidelines for the Diagnosis and Treatment of Chronic Obstructive Pulmonary Disease (Revised 2021)”^39^ are widely adopted among healthcare providers in respiratory departments across China. If an updated version of the guidelines were to propose the above-mentioned interventions that effectively reduce patient dropout at various stages, the COPD care cascade in China could be substantially improved, assuming similar rates of adoption among providers.

Our study has several interrelated strengths and limitations. First, the 29 regions in our sample were selected randomly from all Chinese regions, so our sample is theoretically nationally representative. Although the initial approach was a random selection of regions, many of the regions that were approached refused to participate in the ‘Happy Breathing’ Programme, potentially limiting the representativeness of our results. Given the random exclusion of several important regions from our national sample, it may be argued that our sample is only representative of those regions that were randomly selected. In addition to comprehensive active case finding, the ‘Happy Breathing’ Programme used passive case finding to identify COPD patients who had already received a clinically confirmed diagnosis of COPD in the participating institutions prior to the ‘Happy Breathing’ Programme. Our sample therefore represents the COPD patients among people seeking care for any reason in the Chinese health system.

Using routine health system data from patients at the point of enrolment into the ‘Happy Breathing’ Programme, which is the first and only one of its kind in the country, we were able to perform the first study of COPD care cascade outcomes among people seeking care in China's health system. It should be noted that our data and results cannot be interpreted as representative of China's general population because it excludes those not seeking care in the health system. In a previous COPD prevalence survey in China, researchers adopted a mass screening approach and found a COPD prevalence of 8.6% in adults aged 20 and above.^8^ Compared to such a screening approach aimed at investigating prevalence, the comprehensive active and passive case finding method in this study, which is only targeted at people seeking care in the pilot areas of the programme, will inevitably fall short of identifying all patients with COPD. It is likely that COPD care cascade outcomes are even worse in the general population than those measured in this study since health awareness may be much lower in those who do not seek care and thus would not be enrolled into the ‘Happy Breathing’ Programme.

Second, the ‘Happy Breathing’ Programme used the COPD-SQ to systematically screen patients for COPD who sought care for COPD-unrelated reasons in the Chinese health system. The COPD-SQ has a relatively low sensitivity, which implies that a substantial proportion of people living with COPD will not be identified for enrolment in the program. The reason why this screening questionnaire is used in the ‘Happy Breathing’ Programme is resource constraints. The main alternative approach to COPD screening, which is spirometry, has higher sensitivity than the questionnaire. Screening patients for COPD who sought care for COPD-unrelated reasons in China using spirometry, however, would imply massively larger human resources than a self-administered questionnaire. These resources were not available for the ‘Happy Breathing’ Programme in China. Four implications follow from the comparatively low sensitivity of the initial screening stage of the ‘Happy Breathing’ Programme. First, it will be programmatically important for the ‘Happy Breathing’ Programme and similar initiatives to develop better screening tools for COPD, i.e., screening tools that are as feasible and inexpensive as a self-administered questionnaire but have a substantially higher sensitivity. Second, people who are seeking care in the Chinese health system for COPD-unrelated reasons should be encouraged to repeatedly respond to the questionnaire—it is likely that a large proportion of those who received an initial false-negative screening diagnosis will receive a correct diagnosis when filling out the questionnaire at another visit to the health system—examples of such patients would include those who accidentally miswrote an answer to one of the screening questions or forgot to respond at all to one or more of the questions. Third, in as far as misclassification is random, the misclassification will not bias our sample and our results will still represent the population seeking care in the Chinese health system. Fourth, misclassification does not affect the fact that our results represent a complete baseline for all patients enrolled in the national ‘Happy Breathing’ Programme, because filling out the questionnaire was required for enrolment into the programme. With regards to the COPD-SQ's specificity, it is unlikely that a large proportion of the people enrolled in the ‘Happy Breathing’ Programme was falsely diagnosed with COPD. First, the COPD-SQ already has a relatively high specificity, so that we would not expect many false-positive misdiagnoses at the screening stage. Second, to be enrolled in the ‘Happy Breathing’ Programme, people who screened positive in the COPD-SQ received confirmatory diagnoses with PFT and, as needed, clinical imaging tests. Both the PFT and the imaging tests have even higher specificity than the screening questionnaire, so that most of the false-positive misdiagnoses at the screening stage will have been eliminated at the stage of clinical confirmatory diagnoses.

Third, quality-control specialists were enlisted in each region to verify the logic of the collected data and PFT results in order to ensure data consistency and validity. However, recall bias may have affected study results based on data collected through self-report. To decrease the risk of recall bias, the quality-control staff checked the relationships among variable values based on logic, theory, and expectations from prior empirical finding. For example, quality control staff need to conduct detailed examinations of the acceptability and repeatability of participants' FVC curves in accordance with the guideline for pulmonary function testing in primary care (2018).^78^ If any of the reported information deviated from the expected relationships, the quality-control staff asked the health workers in the ‘Happy Breathing’ Programme to follow up with the patient in question to gather more detailed explanations of their reasons for reporting a particular value. These explanations were then used to re-code the patient's answers, either confirming or changing the previous quantitative reports.

Fourth, the pilot regions were selected in advance, and the surveys started in different years. Each region's performance in the study should thus be interpreted as its performance for a certain period rather than as its current performance. To reduce bias from secular trends, when comparing pilot regions against each other, we benchmarked performance against each region's six-year per-capita GDP from 2014 to 2019 rather than the up-to-date estimate of per-capita GDP.

Lastly, we were unable to divide COPD into emphysema and chronic bronchitis since clinicians in China do not usually differentiate between these conditions. This can be attributed in part to the fact that the 2019 (GOLD) report on COPD diagnosis, management, and prevention (as well as previous GOLD reports) does not differentiate between emphysema and chronic bronchitis in defining COPD.^79^

To our knowledge, this is the first study of COPD care cascade outcomes in the Chinese health system. By examining the COPD care status of all patients enrolled in the national Chinese ‘Happy Breathing’ Programme, we identified concerningly weak performance with respect to the COPD care cascade in the Chinese health system, particularly in the early stages of screening and linkage to care. Future studies should design and test novel interventions to boost COPD screening in the Chinese health system and to support patients in receiving a formal COPD diagnosis in clinics and hospitals. Our study results can also inform targeted strategies for screening at-risk sub-populations. Overall, the extent of loss in the COPD care cascade measured in this study suggests that strong political and financial support may be critical to ease the burden of COPD in China.

## Contributors

CW, SC, WQ and TB conceptualised and designed the study. CW, TY and KH acquired the data and information for this study. CW, WQ, SC and TB conceived and conducted the analyses, processed and visualised the data, and reviewed the literature. FY contributed to data visualisation. TY, QC, PG, LJ and KH contributed to literature review and the interpretation of the data. CW, WQ, SC, TB, LJ, QC, and PG wrote the article; TY and KH critically revised the article. All authors read and approved the final version.

## Data sharing statement

Data is available upon reasonable request to the corresponding authors.

## Editor note

The Lancet Group takes a neutral position with respect to territorial claims in published maps and institutional affiliations.

## Declaration of interests

All authors declare no competing interests.
