# Supplementary material for: The care cascade of chronic obstructive pulmonary disease in China: a cross-sectional study of individual-level data at enrolment into the national ‘Happy Breathing’ Programme
Source: eClinicalMedicine. 2024 Jul 16;74:102597. doi: 10.1016/j.eclinm.2024.102597 (PMC11305216; doi:10.1016/j.eclinm.2024.102597)

**Supplementary Appendix**

**Figure S1. Location of regions and first year of data collection in the ‘Happy Breathing’ Programme in China**

**
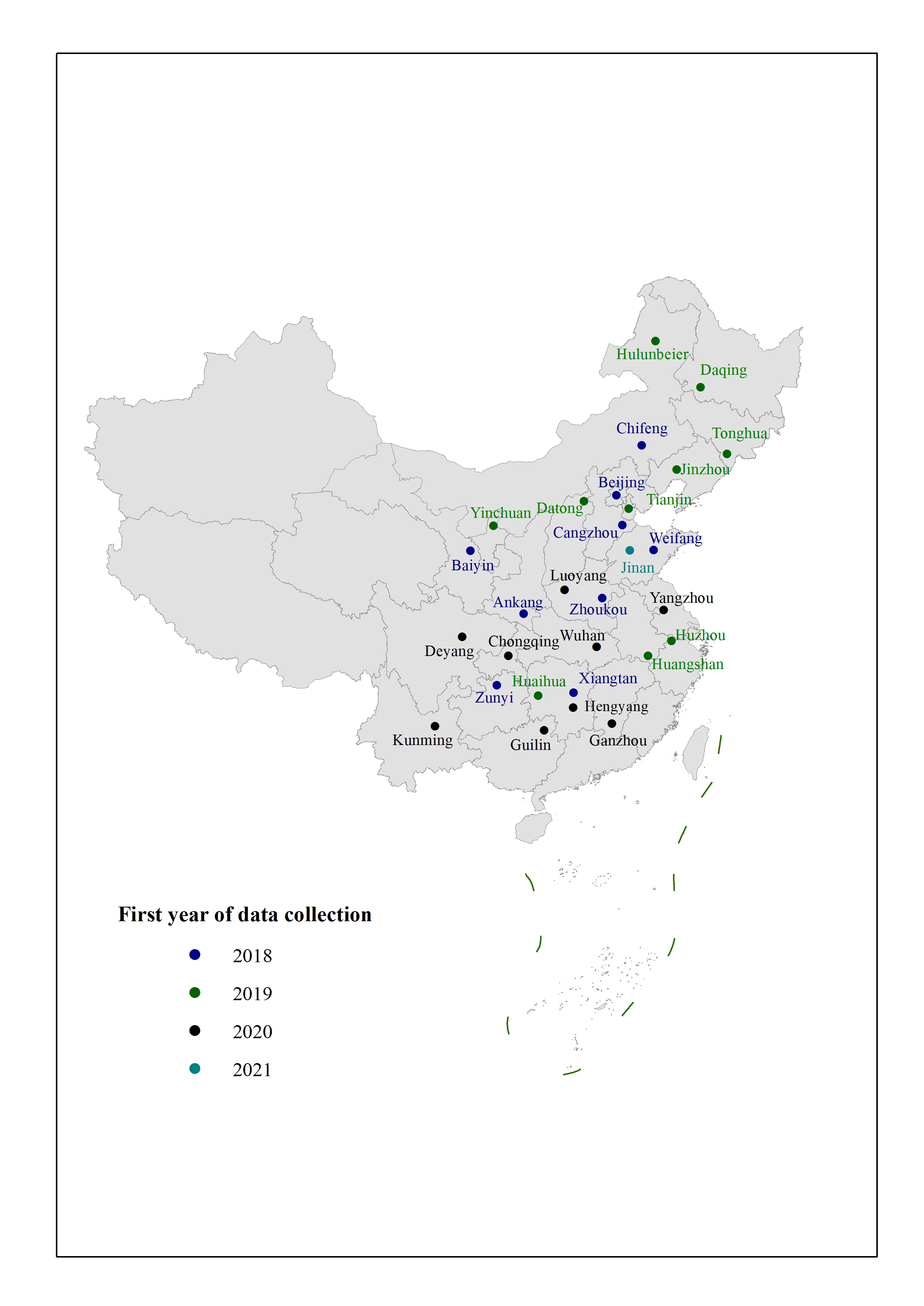
**

Table S1. Univariable and multivariable modified Poisson regressions of each cascade stage onto individual-level variabless. Note: RR=risk ratio; OR=Odds ratio; Controlled 1 = Controlled (mild or no exacerbations), Controlled 2 = Controlled (no exacerbations). Regions with sample size under 100 were not included in this analysis. RR results for sex, age group, body-mass index and tobacco smoking on Controlled 2 cannot be reached in univariable regression, so we substitute these results with the OR values generated through logit regression. Primary education and below refers to having received some primary education or having completed primary school. Secondary education refers to having completed secondary school (including junior and senior high school or technical secondary school). College education refers to having received some type of tertiary education. *Included only one of the variables shown in the table and a binary indicator for each region (region-level fixed effects). †Included sex, age group, education, and a binary indicator for each region (region-level fixed effects). ‡Included sex, age group, education, body-mass index, tobacco smoking, urbanicity, and a binary indicator for each region (region-level fixed effects).

| **Variable** | **Tested** | | **Diagnosed** | | **Treated** | | **Controlled 1** | | **Controlled 2** | |
| --- | --- | --- | --- | --- | --- | --- | --- | --- | --- | --- |
|  | RR (95% CI) | p-value | RR (95% CI) | p-value | RR (95% CI) | p-value | RR (95% CI) | p-value | RR (95% CI) | p-value |
| **Univariable regression*** | |  |  |  |  |  |  |  |  |  |
| **Sex** |  |  |  |  |  |  |  |  |  |  |
| Female | 1 (ref) | - | 1 (ref) | - | 1 (ref) | - | 1 (ref) | - | 1 (ref) | - |
| Male | 1.14 (1.06, 1.23) | <0·001 | 1.31 (1.15, 1.48) | <0·001 | 1.48 (1.27, 1.72) | <0·001 | 1.42 (1.17, 1.72) | <0·001 | 1.52 (1.23, 1.89) | <0·001 |
| **Age, years** |  |  |  |  |  |  |  |  |  |  |
| 54 or younger | 1 (ref) | - | 1 (ref) | - | 1 (ref) | - | 1 (ref) | - | 1 (ref) | - |
| 55-64 | 1.17 (1.10, 1.25) | <0·001 | 2.01 (1.82, 2.22) | <0·001 | 1.89 (1.60, 2.24) | <0·001 | 1.90 (1.59, 2.28) | <0·001 | 1.90 (1.55, 2.31) | <0·001 |
| 65 or older | 1.25 (1.15, 1.37) | <0·001 | 2.46 (2.22, 2.72) | <0·001 | 2.21 (1.87, 2.62) | <0·001 | 1.90 (1.49, 2.41) | <0·001 | 1.92 (1.42, 2.60) | <0·001 |
| **Education** |  |  |  |  |  |  |  |  |  |  |
| Primary education and below | 1 (ref) | - | 1 (ref) | - | 1 (ref) | - | 1 (ref) | - | 1 (ref) | - |
| Secondary education | 1.07 (0.97, 1.18) | 0.18 | 1.07 (0.96, 1.20) | 0.22 | 1.36 (1.20, 1.55) | <0·001 | 1.68 (1.44, 1.96) | <0·001 | 1.78 (1.47, 2.15) | <0·001 |
| College education | 1.17 (1.00, 1.36) | 0.05 | 1.02 (0.81,1.29) | 0.85 | 1.25 (1.05, 1.48) | 0.01 | 1.32 (1.13, 1.55) | <0·001 | 1.32 (1.09, 1.59) | 0.01 |
| **Body-mass index group** |  |  |  |  |  |  |  |  |  |  |
| Underweight | 1.12 (1.05, 1.19) | 0·00 | 1.44 (1.30, 1.60) | <0·001 | 1.23 (1.03, 1.46) | 0.02 | 1.06 (0.80, 1.40) | 0.68 | 1.08 (0.78, 1.49) | 0.66 |
| Healthy weight | 1 (ref) | - | 1 (ref) | - | 1 (ref) | - | 1 (ref) | - | 1 (ref) | - |
| Overweight | 0.92 (0.87, 0.97) | 0·00 | 0.92 (0.86, 0.99) | 0.02 | 0.95 (0.87, 1.03) | 0.22 | 1.00 (0.88, 1.15) | 0.96 | 1.04 (0.90, 1.20) | 0.62 |
| Obese | 0.91 (0.82, 1.00) | 0.05 | 0.84 (0.74, 0.95) | 0.01 | 0.82 (0.69, 0.96) | 0.01 | 0.82 (0.69, 0.97) | 0.02 | 0.81 (0.66, 0.99) | 0.04 |
| **Tobacco smoking** |  |  |  |  |  |  |  |  |  |  |
| Never smoked | 1 (ref) | - | 1 (ref) | - | 1 (ref) | - | 1 (ref) | - | 1 (ref) | - |
| Ever smoked | 0.92 (0.67, 1.25) | 0.57 | 1.16 (0.94, 1.43) | 0.17 | 1.27 (1.12, 1.44) | <0·001 | 1.27 (1.13, 1.43) | <0·001 | 1.33 (1.18, 1.49) | <0·001 |
| **Urbanicity** |  |  |  |  |  |  |  |  |  |  |
| Rural townships | 1 (ref) | - | 1 (ref) | - | 1 (ref) | - | 1 (ref) | - | 1 (ref) | - |
| Urban areas | 1.25 (1.06, 1.47) | 0.01 | 1.13 (0.91, 1.40) | 0.28 | 1.44 (1.17, 1.76) | <0·001 | 1.86 (1,41, 2.46) | <0·001 | 2.02 (1.45, 2.81) | <0·001 |
| **Multivariable regressions with age group, sex, and education†** | | | |  |  |  |  |  |  |  |
| **Sex** |  |  |  |  |  |  |  |  |  |  |
| Female | 1 (ref) | - | 1 (ref) | - | 1 (ref) | - | 1 (ref) | - | 1 (ref) | - |
| Male | 1.12 (1.05, 1.20) | 0·00 | 1.25 (1.11, 1.41) | <0·001 | 1.39 (1.19, 1.63) | <0·001 | 1.32 (1.07, 1.63) | <0·001 | 1.34 (1.09, 1.66) | 0.01 |
| **Age, years** |  |  |  |  |  |  |  |  |  |  |
| 54 and below | 1 (ref) | - | 1 (ref) | - | 1 (ref) | - | 1 (ref) | - | 1 (ref) | - |
| 55-64 | 1.19 (1.11, 1.28) | <0·001 | 2.05 (1.83, 2.30) | <0·001 | 1.96 (1.68, 2.28) | <0·001 | 1.97 (1.68, 2.32) | <0·001 | 1.83 (1.58, 2.12) | <0·001 |
| 65 and above | 1.30 (1.19, 1.43) | <0·001 | 2.59 (2.28, 2.94) | <0·001 | 2.41 (2.05, 2.83) | <0·001 | 2.11 (1.65, 2.70) | <0·001 | 1.99 (1.54, 2.57) | <0·001 |
| **Education** |  |  |  |  |  |  |  |  |  |  |
| Primary education and below | 1 (ref) | - | 1 (ref) | - | 1 (ref) | - | 1 (ref) | - | 1 (ref) | - |
| Secondary education | 1.10 (0.99, 1.22) | 0.07 | 1.17 (1.03, 1.33) | 0.02 | 1.45 (1.27, 1.66) | <0·001 | 1.75 (1.51, 2.03) | <0·001 | 1.83 (1.52, 2.21) | <0·001 |
| College education | 1.25 (1.07, 1.48) | 0.01 | 1.26 (1.00, 1.59) | 0.05 | 1.48 (1.261.74) | <0·001 | 1.52 (1.26, 1.83) | <0·001 | 1.49 (1.19, 1.87) | <0·001 |
|  | | | |  |  |  |  |  |  |  |
| **Multivariable regressions with all variables‡** | | | |  |  |  |  |  |  |  |
| **Sex** |  |  |  |  |  |  |  |  |  |  |
| Female | 1 (ref) | - | 1 (ref) | - | 1 (ref) | - | 1 (ref) | - | 1 (ref) | - |
| Male | 1.18 (1.10, 1.26) | <0·001 | 1.15 (1.04, 1.27) | 0.01 | 1.32 (1.12, 1.57) | 0·00 | 1.31 (1.10, 1.56) | 0·00 | 1.31 (1.21, 1.54) | 0·00 |
| **Age, years** |  |  |  |  |  |  |  |  |  |  |
| 54 and below | 1 (ref) | - | 1 (ref) | - | 1 (ref) | - | 1 (ref) | - | 1 (ref) | - |
| 55-64 | 1.22 (1.13, 1.31) | <0·001 | 2.11 (1.86, 2.39) | <0·001 | 2.07 (1.78, 2.41) | <0·001 | 2.06 (1.74, 2.44) | <0·001 | 1.89 (1.59, 2.25) | <0·001 |
| 65 and above | 1.31 (1.19, 1.44) | <0·001 | 2.56 (2.21, 2.96) | <0·001 | 2.46 (2.11, 2.87) | <0·001 | 2.10 (1.63, 2.69) | <0·001 | 1.96 (1.49, 2.59) | <0·001 |
| **Education** |  |  |  |  |  |  |  |  |  |  |
| Primary education and below | 1 (ref) | - | 1 (ref) | - | 1 (ref) | - | 1 (ref) | - | 1 (ref) | - |
| Secondary education | 1.06 (0.95, 1.17) | 0.30 | 1.15 (1.01, 1.32) | 0.04 | 1.38 (1.17, 1.63) | <0·001 | 1.55 (1.34, 1.80) | <0·001 | 1.59 (1.35, 1.87) | <0·001 |
| College education | 1.18 (1.00, 1.38) | 0.05 | 1.25 (0.98, 1.61) | 0.07 | 1.44 (1.20, 1.72) | <0·001 | 1.37 (1.12, 1.68) | 0·00 | 1.32 (1.03, 1.69) | 0.03 |
| **Body-mass index group** |  |  |  |  |  |  |  |  |  |  |
| Underweight | 1.13 (1.04, 1.24) | 0·00 | 1.39 (1.24, 1.55) | <0·001 | 1.18 (1.01, 1.37) | 0.04 | 1.02 (0.78, 1.32) | 0.90 | 1.00 (0.77, 1.31) | 0.98 |
| Healthy weight | 1 (ref) | - | 1 (ref) | - | 1 (ref) | - | 1 (ref) | - | 1 (ref) | - |
| Overweight | 0.93 (0.87, 0.98) | 0.01 | 0.95 (0.89, 1.01) | 0.09 | 0.91 (0.83, 1.00) | 0.06 | 0.93 (0.80, 1.08) | 0.32 | 0.94 (0.79, 1.12) | 0.50 |
| Obese | 0.91 (0.82, 1.02) | 0.10 | 0.85 (0.73, 0.99) | 0.03 | 0.81 (0.68, 0.96) | 0.02 | 0.76 (0.62, 0.93) | 0.01 | 0.78 (0.65, 0.93) | 0.01 |
| **Tobacco smoking** |  |  |  |  |  |  |  |  |  |  |
| Never smoked | 1 (ref) | - | 1 (ref) | - | 1 (ref) | - | 1 (ref) | - | 1 (ref) | - |
| Ever smoked | 0.82 (0.61, 1.12) | 0.21 | 1.03 (0.85, 1.25) | 0.77 | 1.04 (0.87, 1.25) | 0.66 | 1.04 (0.84, 1.28) | 0.74 | 1.07 (0.85, 1.34) | 0.57 |
| **Urbanicity** |  |  |  |  |  |  |  |  |  |  |
| Rural townships | 1 (ref) | - | 1 (ref) | - | 1 (ref) | - | 1 (ref) | - | 1 (ref) | - |
| Urban areas | 1.19 (1.03, 1.38) | 0.02 | 1.06 (0.87, 1.30) | 0.56 | 1.24 (1.01, 1.52) | 0.04 | 1.43 (1.21, 1.71) | <0·001 | 1.50 (1.21, 1.86) | <0.001 |

Table S2. Univariable and multivariable regressions of each cascade stage onto individual-level variables in low-GDP group. Note: “Low-GDP group” refers to the regions that ranked in the bottom third of average GDP per capita from 2014 to 2019. RRs for female, age group, body-mass index, and tobacco smoking on Controlled 2 cannot be reached in univariable regressions, so we substitute these results with ORs. COPD=chronic obstructive pulmonary disease, Controlled 1 = Controlled (mild or no exacerbations), Controlled 2 = Controlled (no exacerbations). Regions with sample size under 100 were not included in this analysis.

| **Variable** | **Tested** | | **Diagnosed** | | **Treated** | | **Controlled 1** | | **Controlled 2** | |
| --- | --- | --- | --- | --- | --- | --- | --- | --- | --- | --- |
|  | RR (95% CI) | p-value | RR (95% CI) | p-value | RR (95% CI) | p-value | RR (95% CI) | p-value | RR (95% CI) | p-value |
| **Univariable regression** | |  |  |  |  |  |  |  |  |  |
| **Sex** |  |  |  |  |  |  |  |  |  |  |
| Female | 1(ref) | - | 1(ref) | - | 1(ref) | - | 1(ref) | - | 1(ref) | - |
| Male | 1.2 (1.03, 1.39) | 0.02 | 1.31 (1.09, 1.59) | 0.01 | 1.48 (1.21, 1.81) | <0.001 | 1.38 (1.09, 1.74) | 0.01 | 1.42 (1, 2.01) | 0.05 |
| **Age, years** |  |  |  |  |  |  |  |  |  |  |
| 54 and below | 1(ref) | - | 1(ref) | - | 1(ref) | - | 1(ref) | - | 1(ref) | - |
| 55-64 | 1.22 (1.08, 1.38) | <0.001 | 1.96 (1.69, 2.28) | <0.001 | 1.82 (1.38, 2.38) | <0.001 | 1.57 (1.27, 1.95) | <0.001 | 1.61 (0.98, 2.63) | 0.06 |
| 65 and above | 1.33 (1.14, 1.54) | <0.001 | 2.39 (2.13, 2.67) | <0.001 | 2.18 (1.71, 2.77) | <0.001 | 1.42 (1.01, 2) | 0.04 | 1.25 (0.79, 1.99) | 0.34 |
| **Education** |  |  |  |  |  |  |  |  |  |  |
| Primary education and below | 1(ref) | - | 1(ref) | - | 1(ref) | - | 1(ref) | - | 1(ref) | - |
| Secondary education | 1.17 (1.03, 1.34) | 0.02 | 1.07 (0.88, 1.3) | 0.50 | 1.29 (1, 1.66) | 0.05 | 1.83 (1.29, 2.59) | <0.001 | 2.17 (1.27, 3.71) | <0.001 |
| College education | 1.39 (1, 1.93) | 0.05 | 1.3 (0.84, 2.01) | 0.24 | 1.58 (1.26, 1.97) | <0.001 | 2.21 (1.49, 3.26) | <0.001 | 2.31 (1.01, 5.3) | 0.05 |
| **Body-mass index group** | |  |  |  |  |  |  |  |  |  |
| Underweight | 1.09 (0.99, 1.21) | 0.08 | 1.55 (1.38, 1.74) | <0.001 | 1.21 (0.82, 1.77) | 0.34 | 0.83 (0.47, 1.49) | 0.54 | 0.85 (0.5, 1.44) | 0.54 |
| Healthy weight | 1(ref) | - | 1(ref) | - | 1(ref) | - | 1(ref) | - | 1(ref) | - |
| Overweight | 0.96 (0.88, 1.03) | 0.26 | 0.86 (0.77, 0.95) | 0.01 | 0.88 (0.74, 1.06) | 0.17 | 0.99 (0.8, 1.25) | 0.96 | 1.17 (0.82, 1.65) | 0.39 |
| Obese | 0.98 (0.78, 1.22) | 0.83 | 0.76 (0.6, 0.97) | 0.03 | 0.72 (0.52, 1) | 0.05 | 0.63 (0.36, 1.11) | 0.11 | 0.58 (0.27, 1.27) | 0.17 |
| **Tobacco smoking** | |  |  |  |  |  |  |  |  |  |
| Never smoked | 1(ref) | - | 1(ref) | - | 1(ref) | - | 1(ref) | - | 1(ref) | - |
| Ever smoked | 1.14 (1.02, 1.27) | 0.03 | 1.33 (1.17, 1.5) | <0.001 | 1.39 (0.99, 1.96) | 0.06 | 1.34 (1.13, 1.6) | <0.001 | 1.36 (1.13, 1.63) | <0.001 |
| **Urbanicity** |  |  |  |  |  |  |  |  |  |  |
| Rural townships | 1(ref) | - | 1(ref) | - | 1(ref) | - | 1(ref) | - | 1(ref) | - |
| Urban areas | 1.31 (1.16, 1.5) | <0.001 | 1.37 (1.05, 1.8) | 0.02 | 1.63 (1.11, 2.4) | 0.01 | 2.55 (1.28, 5.09) | 0.01 | 3.39 (1.52, 7.55) | <0.001 |
| **Multivariable regressions with age group, sex, and education** | | |  |  |  |  |  |  |  |  |
| **Sex** |  |  |  |  |  |  |  |  |  |  |
| Female | 1(ref) | - | 1(ref) | - | 1(ref) | - | 1(ref) | - | 1(ref) | - |
| Male | 1.16 (1, 1.33) | 0.05 | 1.24 (1.05, 1.46) | 0.01 | 1.4 (1.14, 1.73) | <0.001 | 1.27 (0.96, 1.68) | 0.10 | 1.27 (0.89, 1.81) | 0.18 |
| **Age, years** |  |  |  |  |  |  |  |  |  |  |
| 54 and below | 1(ref) | - | 1(ref) | - | 1(ref) | - | 1(ref) | - | 1(ref) | - |
| 55-64 | 1.27 (1.09, 1.49) | <0.001 | 2.08 (1.69, 2.55) | <0.001 | 1.95 (1.56, 2.45) | <0.001 | 1.88 (1.51, 2.34) | <0.001 | 2.09 (1.63, 2.68) | <0.001 |
| 65 and above | 1.44 (1.21, 1.72) | <0.001 | 2.66 (2.22, 3.19) | <0.001 | 2.49 (2.05, 3.01) | <0.001 | 1.91 (1.26, 2.89) | <0.001 | 1.81 (1.01, 3.22) | 0.05 |
| **Education** |  |  |  |  |  |  |  |  |  |  |
| Primary education and below | 1(ref) | - | 1(ref) | - | 1(ref) | - | 1(ref) |  | 1(ref) | - |
| Secondary education | 1.22 (1.07, 1.4) | <0.001 | 1.18 (0.97, 1.44) | 0.10 | 1.41 (1.07, 1.86) | 0.01 | 1.9 (1.31, 2.78) | <0.001 | 2.14 (1.21, 3.8) | 0.01 |
| College education | 1.57 (1.15, 2.14) | 0.01 | 1.7 (1.2, 2.4) | <0.001 | 2.02 (1.66, 2.47) | <0.001 | 2.67 (1.58, 4.51) | <0.001 | 3.06 (1.22, 7.71) | 0.02 |
| **Multivariable regressions with all variables** | | | | |  |  |  |  |  |  |
| **Sex** |  |  |  |  |  |  |  |  |  |  |
| Female | 1(ref) | - | 1(ref) | - | 1(ref) | - | 1(ref) | - | 1(ref) | - |
| Male | 1.18 (1.01, 1.37) | 0.03 | 1.16 (1, 1.34) | 0.05 | 1.24 (0.92, 1.67) | 0.15 | 1.44 (0.88, 2.36) | 0.14 | 1.7 (0.95, 3.03) | 0.07 |
| **Age, years** |  |  |  |  |  |  |  |  |  |  |
| 54 and below | 1(ref) | - | 1(ref) | - | 1(ref) | - | 1(ref) | - | 1(ref) | - |
| 55-64 | 1.33 (1.14, 1.54) | <0.001 | 2.20 (1.78, 2.72) | <0.001 | 2.3 (1.75, 3.01) | <0.001 | 2.24 (1.55, 3.23) | <0.001 | 2.71 (1.87, 3.94) | <0.001 |
| 65 and above | 1.45 (1.2, 1.76) | <0.001 | 2.65 (2.04, 3.45) | <0.001 | 2.73 (2.21, 3.37) | <0.001 | 1.93 (1.31, 2.85) | <0.001 | 1.94 (1.11, 3.4) | 0.02 |
| **Education** |  |  |  |  |  |  |  |  |  |  |
| Primary education and below | 1(ref) | - | 1(ref) | - | 1(ref) | - | 1(ref) | - | 1(ref) | - |
| Secondary education | 1.16 (1.01, 1.34) | 0.04 | 1.12 (0.85, 1.48) | 0.43 | 1.29 (0.89, 1.89) | 0.18 | 1.46 (0.95, 2.25) | 0.08 | 1.34 (0.74, 2.42) | 0.33 |
| College education | 1.46 (1.02, 2.1) | 0.04 | 1.59 (1.01, 2.52) | 0.05 | 1.78 (1.4, 2.28) | <0.001 | 1.85 (1.1, 3.12) | 0.02 | 1.59 (0.61, 4.17) | 0.34 |
| **Body-mass index group** | |  |  |  |  |  |  |  |  |  |
| Underweight | 1.10 (0.98, 1.24) | 0.12 | 1.52 (1.38, 1.67) | <0.001t | 1.2 (0.87, 1.67) | 0.27 | 0.92 (0.57, 1.51) | 0.75 | 0.89 (0.57, 1.39) | 0.61 |
| Healthy weight | 1(ref) | - | 1(ref) | - | 1(ref) | - | 1(ref) | - | 1(ref) | - |
| Overweight | 0.98 (0.89, 1.08) | 0.71 | 0.92 (0.81, 1.04) | 0.18 | 0.92 (0.74, 1.14) | 0.43 | 1.03 (0.73, 1.45) | 0.87 | 1.22 (0.94, 1.59) | 0.13 |
| Obese | 0.98 (0.76j, 1.27) | 0.87 | 0.72 (0.6, 0.86) | <0.001 | 0.73 (0.53, 0.99) | 0.04 | 0.66 (0.36, 1.2) | 0.17 | 0.74 (0.39, 1.4) | 0.36 |
| **Tobacco smoking** | |  |  |  |  |  |  |  |  |  |
| Never smoked | 1(ref) | - | 1(ref) | - | 1(ref) | - | 1(ref) | - | 1(ref) | - |
| Ever smoked | 0.99 (0.89, 1.11) | 0.89 | 1.15 (1.03, 1.28) | 0.01 | 1.17 (0.77, 1.77) | 0.47 | 1.04 (0.85, 1.28) | 0.71 | 1.01 (0.86, 1.2) | 0.87 |
| **Urbanicity** |  |  |  |  |  |  |  |  |  |  |
| Rural townships | 1(ref) | - | 1(ref) | - | 1(ref) | - | 1(ref) | - | 1(ref) | - |
| Urban areas | 1.18 (1.03, 1.35) | 0.02 | 1.24 (0.95, 1.62) | 0.11 | 1.34 (0.86, 2.1) | 0.19 | 1.58 (1.02, 2.46) | 0.04 | 2.03 (1.23, 3.36) | 0.01 |

Table S3. Univariable and multivariable regressions of each cascade stage onto individual-level variables in middle-GDP group. Note: “Middle-GDP group” refers to the regions that ranked in the middle third of average GDP per capita from 2014 to 2019. RRs for female, age group, body-mass index, and tobacco smoking on Controlled 2 cannot be reached in univariable regressions, so we substitute these results with ORs. COPD=chronic obstructive pulmonary disease, Controlled 1 = Controlled (mild or no exacerbations), Controlled 2 = Controlled (no exacerbations). Regions with sample size under 100 were not included in this analysis.

| **Variable** | **Tested** | | **Diagnosed** | | **Treated** | | **Controlled 1** | | **Controlled 2** | |
| --- | --- | --- | --- | --- | --- | --- | --- | --- | --- | --- |
|  | RR (95% CI) | p-value | RR (95% CI) | p-value | RR (95% CI) | p-value | RR (95% CI) | p-value | RR (95% CI) | p-value |
| **Univariable regression** | |  |  |  |  |  |  |  |  |  |
| **Sex** |  |  |  |  |  |  |  |  |  |  |
| Female | 1(ref) | - | 1(ref) | - | 1(ref) | - | 1(ref) | - | 1(ref) | - |
| Male | 1.10 (0.99, 1.22) | 0.07 | 1.34 (1.05, 1.7) | 0.02 | 1.58 (1.25, 2.01) | <0.001 | 1.66 (1.19, 2.32) | <0.001 | 1.51 (1.07, 2.15) | 0.02 |
| **Age, years** |  |  |  |  |  |  |  |  |  |  |
| 54 and below | 1(ref) | - | 1(ref) | - | 1(ref) | - | 1(ref) | - | 1(ref) | - |
| 55-64 | 1.12 (1.05, 1.19) | <0.001 | 1.99 (1.65, 2.41) | <0.001 | 1.85 (1.32, 2.61) | <0.001 | 1.92 (1.05, 3.5) | 0.03 | 1.39 (0.82, 2.38) | 0.22 |
| 65 and above | 1.20 (1.08, 1.35) | <0.001 | 2.45 (1.85, 3.26) | <0.001 | 1.95 (1.41, 2.71) | <0.001 | 1.48 (1.01, 2.16) | 0.04 | 1.23 (0.76, 2.01) | 0.40 |
| **Education** |  |  |  |  |  |  |  |  |  |  |
| Primary education and below | 1(ref) | - | 1(ref) | - | 1(ref) | - | 1(ref) | - | 1(ref) | - |
| Secondary education | 1.07 (0.76, 1.52) | 0.69 | 0.8 (0.55, 1.16) | 0.23 | 1.02 (0.73, 1.44) | 0.90 | 1.54 (0.88, 2.7) | 0.13 | 1.62 (0.77, 3.4) | 0.20 |
| College education | 0.99 (0.83, 1.18) | 0.92 | 1.1 (0.91, 1.32) | 0.33 | 1.39 (1.09, 1.78) | 0.01 | 1.43 (1.11, 1.84) | 0.01 | 1.43 (1, 2.05) | 0.05 |
| **Body-mass index group** | |  |  |  |  |  |  |  |  |  |
| Underweight | 1.16 (1.04, 1.29) | 0.01 | 1.44 (1.22, 1.7) | <0.001 | 1.39 (1.18, 1.64) | <0.001 | 1.49 (1.07, 2.08) | 0.02 | 1.57 (1.06, 2.35) | 0.03 |
| Healthy weight | 1(ref) | - | 1(ref) | - | 1(ref) | - | 1(ref) | - | 1(ref) | - |
| Overweight | 0.88 (0.79, 0.98) | 0.02 | 0.94 (0.84, 1.06) | 0.31 | 1.03 (0.92, 1.15) | 0.65 | 0.91 (0.76, 1.09) | 0.32 | 0.96 (0.67, 1.39) | 0.85 |
| Obese | 0.89 (0.77, 1.02) | 0.08 | 0.93 (0.66, 1.33) | 0.71 | 0.94 (0.57, 1.56) | 0.82 | 0.84 (0.36, 1.98) | 0.69 | 1.01 (0.56, 1.83) | 0.97 |
| **Tobacco smoking** | |  |  |  |  |  |  |  |  |  |
| Never smoked | 1(ref) | - | 1(ref) | - | 1(ref) | - | 1(ref) | - | 1(ref) | - |
| Ever smoked | 0.69 (0.35, 1.36) | 0.29 | 0.93 (0.47, 1.82) | 0.83 | 1.27 (1.03, 1.56) | 0.03 | 1.53 (1.16, 2.01) | <0.001 | 1.51 (1.1, 2.08) | 0.01 |
| **Urbanicity** |  |  |  |  |  |  |  |  |  |  |
| Rural townships | 1(ref) | - | 1(ref) | - | 1(ref) | - | 1(ref) | - | 1(ref) | - |
| Urban areas | 1.38 (1.09, 1.74) | 0.01 | 1.15 (0.84, 1.57) | 0.38 | 1.39 (1.01, 1.92) | 0.05 | 1.63 (1.14, 2.33) | 0.01 | 1.63 (1.14, 2.33) | 0.01 |
| **Multivariable regressions with age group, sex, and education** | | |  |  |  |  |  |  |  |  |
| **Sex** |  |  |  |  |  |  |  |  |  |  |
| Female | 1(ref) | - | 1(ref) | - | 1(ref) | - | 1(ref) | - | 1(ref) | - |
| Male | 1.1 (0.98, 1.23) | 0.10 | 1.3 (1.03, 1.65) | 0.03 | 1.51 (1.21, 1.88) | <0.001 | 1.51 (1.12, 2.02) | 0.01 | 1.41 (1.05, 1.9) | 0.02 |
| **Age, years** |  |  |  |  |  |  |  |  |  |  |
| 54 and below | 1(ref) | - | 1(ref) | - | 1(ref) | - | 1(ref) | - | 1(ref) | - |
| 55-64 | 1.13 (1.03, 1.24) | 0.01 | 2 (1.58, 2.53) | <0.001 | 1.83 (1.29, 2.61) | <0.001 | 1.91 (1.02, 3.58) | 0.04 | 1.48 (0.76, 2.87) | 0.25 |
| 65 and above | 1.22 (1.13, 1.31) | <0.001 | 2.55 (1.75, 3.74) | <0.001 | 2.09 (1.48, 2.94) | <0.001 | 1.59 (1.04, 2.41) | 0.03 | 1.38 (0.87, 2.18) | 0.17 |
| **Education** |  |  |  |  |  |  |  |  |  |  |
| Primary education and below | 1(ref) | - | 1(ref) | - | 1(ref) | - | 1(ref) | - | 1(ref) | - |
| Secondary education | 1.02 (0.85, 1.23) | 0.82 | 1.23 (0.92, 1.64) | 0.16 | 1.46 (1.11, 1.91) | 0.01 | 1.4 (1.11, 1.77) | 0.01 | 1.41 (0.99, 2.01) | 0.05 |
| College education | 1.13 (0.78, 1.63) | 0.52 | 0.96 (0.61, 1.51) | 0.85 | 1.08 (0.86, 1.35) | 0.53 | 1.49 (0.82, 2.72) | 0.19 | 1.7 (0.78, 3.7) | 0.18 |
| **Multivariable regressions with all variables** | | | | |  |  |  |  |  |  |
| **Sex** |  |  |  |  |  |  |  |  |  |  |
| Female | 1(ref) | - | 1(ref) | - | 1(ref) | - | 1(ref) | - | 1(ref) | - |
| Male | 1.2 (1.05, 1.38) | 0.01 | 1.23 (0.91, 1.66) | 0.18 | 1.46 (0.98, 2.16) | 0.06 | 1.28 (0.86, 1.9) | 0.22 | 1.12 (0.71, 1.77) | 0.62 |
| **Age, years** |  |  |  |  |  |  |  |  |  |  |
| 54 and below | 1(ref) | - | 1(ref) | - | 1(ref) | - | 1(ref) | - | 1(ref) | - |
| 55-64 | 1.16 (1.09, 1.23) | <0.001 | 2.18 (1.75, 2.71) | <0.001 | 2.08 (1.55, 2.81) | <0.001 | 2.37 (1.46, 3.84) | <0.001 | 1.79 (1.04, 3.06) | 0.03 |
| 65 and above | 1.23 (1.15, 1.33) | <0.001 | 2.69 (1.93, 3.75) | <0.001 | 2.4 (1.73, 3.34) | <0.001 | 1.91 (1.16, 3.15) | 0.01 | 1.56 (0.88, 2.76) | 0.13 |
| **Education** |  |  |  |  |  |  |  |  |  |  |
| Primary education and below | 1(ref) | - | 1(ref) | - | 1(ref) | - | 1(ref) | - | 1(ref) | - |
| Secondary education | 0.97 (0.81, 1.17) | 0.78 | 1.22 (0.93, 1.6) | 0.15 | 1.44 (1.08, 1.93) | 0.01 | 1.41 (0.96, 2.07) | 0.08 | 1.42 (0.92, 2.17) | 0.11 |
| College education | 0.95 (0.71, 1.25) | 0.70 | 0.9 (0.56, 1.43) | 0.65 | 0.95 (0.78, 1.15) | 0.60 | 1.21 (0.62, 2.38) | 0.58 | 1.35 (0.6, 3.08) | 0.47 |
| **Body-mass index group** | |  |  |  |  |  |  |  |  |  |
| Underweight | 1.18 (1.06, 1.31) | <0.001 | 1.42 (1.23, 1.63) | <0.001 | 1.33 (1.08, 1.63) | 0.01 | 1.43 (0.92, 2.21) | 0.11 | 1.46 (0.93, 2.3) | 0.10 |
| Healthy weight | 1(ref) | - | 1(ref) | - | 1(ref) | - | 1(ref) | - | 1(ref) | - |
| Overweight | 0.89 (0.81, 1) | 0.04 | 0.98 (0.89, 1.09) | 0.75 | 0.99 (0.85, 1.15) | 0.87 | 0.86 (0.7, 1.05) | 0.14 | 0.86 (0.68, 1.1) | 0.23 |
| Obese | 0.9 (0.83, 0.97) | 0.01 | 1.02 (0.7, 1.49) | 0.91 | 0.96 (0.54, 1.7) | 0.88 | 0.73 (0.25, 2.16) | 0.57 | 0.84 (0.31, 2.3) | 0.74 |
| **Tobacco smoking** | |  |  |  |  |  |  |  |  |  |
| Never smoked | 1(ref) | - | 1(ref) | - | 1(ref) | - | 1(ref) | - | 1(ref) | - |
| Ever smoked | 0.64 (0.35, 1.16) | 0.14 | 0.8 (0.49, 1.32) | 0.39 | 1 (0.8, 1.25) | 1.00 | 1.23 (0.86, 1.76) | 0.27 | 1.39 (0.88, 2.21) | 0.16 |
| **Urbanicity** |  |  |  |  |  |  |  |  |  |  |
| Rural townships | 1(ref) | - | 1(ref) | - | 1(ref) | - | 1(ref) | - | 1(ref) | - |
| Urban areas | 1.32 (1.19, 1.47) | <0.001 | 1.09 (0.86, 1.4) | 0.47 | 1.21 (0.9, 1.64) | 0.21 | 1.41 (0.92, 2.16) | 0.12 | 1.47 (0.88, 2.46) | 0.14 |

Table S4. Univariable and multivariable regressions of each cascade stage onto individual-level variables in high-GDP group. Note: “High-GDP group” refers to the regions that ranked in the top third of average GDP per capita from 2014 to 2019. RRs for female, age group, body-mass index, and tobacco smoking on Controlled 2 cannot be reached in univariable regressions, so we substitute these results with ORs. COPD=chronic obstructive pulmonary disease, Controlled 1 = Controlled (mild or no exacerbations), Controlled 2 = Controlled (no exacerbations). Regions with sample size under 100 were not included in this analysis.

| **Variable** | **Tested** | | **Diagnosed** | | **Treated** | | **Controlled 1** | | **Controlled 2** | |
| --- | --- | --- | --- | --- | --- | --- | --- | --- | --- | --- |
|  | RR (95% CI) | p-value | RR (95% CI) | p-value | RR (95% CI) | p-value | RR (95% CI) | p-value | RR (95% CI) | p-value |
| **Univariable regression** | |  |  |  |  |  |  |  |  |  |
| **Sex** |  |  |  |  |  |  |  |  |  |  |
| Female | 1(ref) | - | 1(ref) | - | 1(ref) | - | 1(ref) | - | 1(ref) | - |
| Male | 1.13 (1.04, 1.24) | 0.01 | 1.27 (1.02, 1.59) | 0.03 | 1.43 (1.11, 1.83) | 0.01 | 1.4 (1.07, 1.83) | 0.02 | 1.55 (1.31, 1.83) | <0.001 |
| **Age, years** |  |  |  |  |  |  |  |  |  |  |
| 54 and below | 1(ref) | - | 1(ref) | - | 1(ref) | - | 1(ref) | - | 1(ref) | - |
| 55-64 | 1.18 (1.08, 1.29) | <0.001 | 2.11 (1.66, 2.69) | <0.001 | 2.03 (1.5, 2.75) | <0.001 | 2.17 (1.67, 2.83) | <0.001 | 2.24 (1.63, 3.07) | <0.001 |
| 65 and above | 1.25 (1.12, 1.39) | <0.001 | 2.58 (2.17, 3.07) | <0.001 | 2.44 (1.91, 3.12) | <0.001 | 2.36 (1.92, 2.89) | <0.001 | 2.51 (1.87, 3.36) | <0.001 |
| **Education** |  |  |  |  |  |  |  |  |  |  |
| Primary education and below | 1(ref) | - | 1(ref) | - | 1(ref) | - | 1(ref) | - | 1(ref) | - |
| Secondary education | 1.09 (1.02, 1.16) | 0.01 | 0.96 (0.79, 1.16) | 0.66 | 1.23 (0.97, 1.54) | 0.08 | 1.24 (1.02, 1.51) | 0.03 | 1.22 (1, 1.49) | 0.05 |
| College education | 1.01 (0.92, 1.12) | 0.79 | 1.02 (0.88, 1.17) | 0.82 | 1.35 (1.1, 1.67) | <0.001 | 1.69 (1.38, 2.08) | <0.001 | 1.78 (1.4, 2.28) | <0.001 |
| **Body-mass index group** | |  |  |  |  |  |  |  |  |  |
| Underweight | 1.09 (1, 1.2) | 0.06 | 1.26 (1.1, 1.44) | <0.001 | 1.12 (0.92, 1.36) | 0.27 | 0.98 (0.73, 1.34) | 0.92 | 0.94 (0.67, 1.32) | 0.72 |
| Healthy weight | 1(ref) | - | 1(ref) | - | 1(ref) | - | 1(ref) | - | 1(ref) | - |
| Overweight | 0.92 (0.87, 0.98) | 0.01 | 0.95 (0.87, 1.05) | 0.35 | 0.93 (0.82, 1.05) | 0.23 | 1.01 (0.84, 1.22) | 0.88 | 1.02 (0.86, 1.21) | 0.81 |
| Obese | 0.88 (0.76, 1.01) | 0.07 | 0.85 (0.76, 0.97) | 0.01 | 0.83 (0.73, 0.95) | 0.01 | 0.85 (0.74, 0.98) | 0.02 | 0.8 (0.62, 1.05) | 0.11 |
| **Tobacco smoking** | |  |  |  |  |  |  |  |  |  |
| Never smoked | 1(ref) | - | 1(ref) | - | 1(ref) | - | 1(ref) | - | 1(ref) | - |
| Ever smoked | 0.99 (0.96, 1.02) | 0.42 | 1.18 (1.07, 1.31) | <0.001 | 1.17 (1.07, 1.27) | <0.001 | 1.18 (1.06, 1.32) | <0.001 | 1.22 (1.09, 1.36) | <0.001 |
| **Urbanicity** |  |  |  |  |  |  |  |  |  |  |
| Rural townships | 1(ref) | - | 1(ref) | - | 1(ref) | - | 1(ref) | - | 1(ref) | - |
| Urban areas | 0.92 (0.82, 1.03) | 0.14 | 0.82 (0.75, 0.89) | <0.001 | 1.25 (1.0ssh, 1.56) | 0.05 | 1.64 (1.23, 2.17) | <0.001 | 1.65 (1.29, 2.13) | <0.001 |
| **Multivariable regressions with age group, sex, and education** | | |  |  |  |  |  |  |  |  |
| **Sex** |  |  |  |  |  |  |  |  |  |  |
| Female | 1(ref) | - | 1(ref) | - | 1(ref) | - | 1(ref) | - | 1(ref) | - |
| Male | 1.11 (1.01, 1.22) | 0.04 | 1.22 (0.97, 1.54) | 0.08 | 1.33 (1, 1.75) | 0.05 | 1.29 (0.96, 1.75) | 0.1 | 1.33 (0.99, 1.8) | 0.06 |
| **Age, years** |  |  |  |  |  |  |  |  |  |  |
| 54 and below | 1(ref) | - | 1(ref) | - | 1(ref) | - | 1(ref) | - | 1(ref) | - |
| 55-64 | 1.19 (1.11, 1.28) | <0.001 | 2.12 (1.72, 2.61) | <0.001 | 2.08 (1.57, 2.75) | <0.001 | 2.15 (1.71, 2.7) | <0.001 | 1.97 (1.67, 2.33) | <0.001 |
| 65 and above | 1.26 (1.11, 1.43) | <0.001 | 2.61 (2.24, 3.05) | <0.001 | 2.6 (1.99, 3.4) | <0.001 | 2.47 (1.89, 3.22) | <0.001 | 2.31 (1.88, 2.85) | <0.001 |
| **Education** |  |  |  |  |  |  |  |  |  |  |
| Primary education and below | 1(ref) | - | 1(ref) | - | 1(ref) | - | 1(ref) | - | 1(ref) | - |
| Secondary education | 1.03 (0.94, 1.13) | 0.49 | 1.09 (0.96, 1.24) | 0.17 | 1.46 (1.22, 1.75) | <0.001 | 1.78 (1.49, 2.13) | <0.001 | 1.86 (1.5, 2.31) | <0.001 |
| College education | 1.14 (1.09, 1.2) | <0.001 | 1.15 (0.96, 1.38) | 0.13 | 1.48 (1.21, 1.82) | <0.001 | 1.47 (1.18, 1.83) | <0.001 | 1.42 (1.12, 1.79) | <0.001 |
| **Multivariable regressions with all variables** | | | | |  |  |  |  |  |  |
| **Sex** |  |  |  |  |  |  |  |  |  |  |
| Female | 1(ref) | - | 1(ref) | - | 1(ref) | - | 1(ref) | - | 1(ref) | - |
| Male | 1.11 (1.02, 1.21) | 0.02 | 1.06 (0.94, 1.2) | 0.32 | 1.29 (1, 1.68) | 0.05 | 1.29 (1.03, 1.61) | 0.03 | 1.3 (1.09, 1.56) | <0.001 |
| **Age, years** |  |  |  |  |  |  |  |  |  |  |
| 54 and below | 1(ref) | - | 1(ref) | - | 1(ref) | - | 1(ref) | - | 1(ref) | - |
| 55-64 | 1.16 (1.1, 1.23) | <0.001 | 1.99 (1.82, 2.17) | <0.001 | 1.91 (1.5, 2.43) | <0.001 | 1.94 (1.6, 2.35) | <0.001 | 1.77 (1.46, 2.16) | <0.001 |
| 65 and above | 1.25 (1.07, 1.46) | 0.01 | 2.36 (2.11, 2.64) | <0.001 | 2.29 (1.66, 3.17) | <0.001 | 2.21 (1.58, 3.09) | <0.001 | 2.07 (1.48, 2.89) | <0.001 |
| **Education** |  |  |  |  |  |  |  |  |  |  |
| Primary education and below | 1(ref) | - | 1(ref) | - | 1(ref) | - | 1(ref) | - | 1(ref) | - |
| Secondary education | 1.04 (0.99, 1.11) | 0.15 | 1.14 (1.07, 1.22) | <0.001 | 1.42 (1.19, 1.69) | <0.001 | 1.64 (1.51, 1.79) | <0.001 | 1.72 (1.57, 1.9) | <0.001 |
| College education | 1.20 (1.13, 1.26) | <0.001 | 1.22 (1.05, 1.41) | 0.01 | 1.51 (1.29, 1.76) | <0.001 | 1.39 (1.11, 1.74) | <0.001 | 1.35 (1.08, 1.7) | 0.01 |
| **Body-mass index group** | |  |  |  |  |  |  |  |  |  |
| Underweight | 1.09 (1.02, 1.15) | 0.01 | 1.14 (1.03, 1.28) | 0.02 | 1.02 (0.85, 1.22) | 0.84 | 0.87 (0.69, 1.1) | 0.25 | 0.86 (0.7, 1.05) | 0.14 |
| Healthy weight | 1(ref) | - | 1(ref) | - | 1(ref) | - | 1(ref) | - | 1(ref) | - |
| Overweight | 0.92 (0.86, 0.98) | 0.01 | 0.95 (0.85, 1.07) | 0.41 | 0.87 (0.74, 1.02) | 0.09 | 0.92 (0.74, 1.14) | 0.44 | 0.91 (0.71, 1.18) | 0.49 |
| Obese | 0.88 (0.73, 1.06) | 0.17 | 0.85 (0.7, 1.02) | 0.08 | 0.79 (0.68, 0.91) | <0.001 | 0.78 (0.65, 0.94) | 0.01 | 0.77 (0.67, 0.9) | <0.001 |
| **Tobacco smoking** | |  |  |  |  |  |  |  |  |  |
| Never smoked | 1(ref) | - | 1(ref) | - | 1(ref) | - | 1(ref) | - | 1(ref) | - |
| Ever smoked | 0.93 (0.87, 0.99) | 0.02 | 1.12 (0.93, 1.35) | 0.23 | 0.99 (0.73, 1.34) | 0.95 | 0.98 (0.73, 1.33) | 0.91 | 1.01 (0.76, 1.34) | 0.96 |
| **Urbanicity** |  |  |  |  |  |  |  |  |  |  |
| Rural townships | 1(ref) | - | 1(ref) | - | 1(ref) | - | 1(ref) | - | 1(ref) | - |
| Urban areas | 0.89 (0.80, 1.00) | 0.05 | 0.79 (0.73, 0.87) | <0.001 | 1.14 (1, 1.31) | 0.06 | 1.39 (1.19, 1.63) | <0.001 | 1.36 (1.2, 1.53) | <0.001 |

Table S5. Univariable and multivariable regressions of each cascade stage onto individual-level variables by mMRC. Note: COPD= chronic obstructive pulmonary disease, Controlled 1 = Controlled (mild or no exacerbations), Controlled 2 = Controlled (no exacerbations). Regions with sample size under 100 were not included in this analysis.

| **Variable** | **Tested** | | **Diagnosed** | | **Treated** | | **Controlled 1** | | **Controlled 2** | |
| --- | --- | --- | --- | --- | --- | --- | --- | --- | --- | --- |
|  | RR (95% CI) | p-value | RR (95% CI) | p-value | RR (95% CI) | p-value | RR (95% CI) | p-value | RR (95% CI) | p-value |
| **mMRC = 0** | |  |  |  |  |  |  |  |  |  |
| **Univariable regression** | |  |  |  |  |  |  |  |  |  |
| **Sex** |  |  |  |  |  |  |  |  |  |  |
| Female | 1(ref) | - | 1(ref) | - | 1(ref) | - | 1(ref) | - | 1(ref) | - |
| Male | 1.06(0.89,1.25) | 0.53 | 1.60(1.00,2.54) | 0.05 | 1.68(0.93,3.04) | 0.08 | 1.48(0.98,2.23) | 0.06 | 1.33(1.01,1.74) | 0.04 |
| **Age, years** |  |  |  |  |  |  |  |  |  |  |
| 54 and below | 1(ref) | - | 1(ref) | - | 1(ref) | - | 1(ref) | - | 1(ref) | - |
| 55-64 | 1.09(0.95,1.26) | 0.21 | 1.98(1.50,2.60) | <0.001 | 3.14(1.72,5.72) | <0.001 | 3.19(1.95,5.22) | <0.001 | 2.76(1.93,3.95) | <0.001 |
| 65 and above | 1.19(1.01,1.39) | 0.03 | 2.11(1.53,2.91) | <0.001 | 3.35(2.08,5.41) | <0.001 | 3.52(2.24,5.52) | <0.001 | 2.63(1.85,3.75) | <0.001 |
| **Education** |  |  |  |  |  |  |  |  |  |  |
| Primary education and below | 1(ref) | - | 1(ref) | - | 1(ref) | - | 1(ref) | - | 1(ref) | - |
| Secondary education | 1.27(1.08,1.49) | 0.00 | 1.28(0.95,1.73) | 0.11 | 1.28(0.76,2.16) | 0.35 | 1.18(0.87,1.61) | 0.28 | 1.27(0.83,1.96) | 0.27 |
| College education | 1.04(0.88,1.23) | 0.62 | 1.47(1.01,2.13) | 0.04 | 1.52(0.96,2.41) | 0.07 | 1.71(1.02,2.87) | 0.04 | 2.26(1.42,3.59) | 0.00 |
| **Body-mass index group** | |  |  |  |  |  |  |  |  |  |
| Underweight | 1.16(0.93,1.45) | 0.20 | 0.97(0.56,1.68) | 0.92 | 0.62(0.37,1.07) | 0.08 | 0.93(0.57,1.49) | 0.75 | 1.05(0.57,1.91) | 0.88 |
| Healthy weight | 1(ref) | - | 1(ref) | - | 1(ref) | - | 1(ref) | - | 1(ref) | - |
| Overweight | 0.97(0.84,1.12) | 0.65 | 0.99(0.83,1.19) | 0.94 | 0.75(0.57,0.98) | 0.04 | 0.84(0.66,1.07) | 0.15 | 1.07(0.85,1.36) | 0.56 |
| Obese | 1.02(0.81,1.28) | 0.88 | 0.76(0.47,1.22) | 0.26 | 0.65(0.33,1.31) | 0.23 | 0.58(0.28,1.23) | 0.16 | 0.67(0.35,1.28) | 0.22 |
| **Tobacco smoking** | |  |  |  |  |  |  |  |  |  |
| Never smoked | 1(ref) | - | 1(ref) | - | 1(ref) | - | 1(ref) | - | 1(ref) | - |
| Ever smoked | 0.74(0.47,1.17) | 0.20 | 1.19(0.95,1.49) | 0.14 | 1.23(0.93,1.63) | 0.14 | 1.19(0.84,1.70) | 0.33 | 1.21(0.79,1.83) | 0.38 |
| **Urbanicity** |  |  |  |  |  |  |  |  |  |  |
| Rural townships | 1(ref) | - | 1(ref) | - | 1(ref) | - | 1(ref) | - | 1(ref) | - |
| Urban areas | 1.45(1.10,1.90) | 0.01 | 1.46(0.94,2.26) | 0.09 | 1.27(0.86,1.86) | 0.23 | 1.20(0.84,1.72) | 0.31 | 1.31(0.72,2.37) | 0.37 |
| **Multivariable regressions with age group, sex, and education** | | | | |  |  |  |  |  |  |
| **Sex** |  |  |  |  |  |  |  |  |  |  |
| Female | 1(ref) | - | 1(ref) | - | 1(ref) | - | 1(ref) | - | 1(ref) | - |
| Male | 1.04(0.88,1.23) | 0.63 | 1.46(0.88,2.44) | 0.15 | 1.44(0.81,2.56) | 0.21 | 1.26(0.81,1.98) | 0.30 | 1.15(0.85,1.56) | 0.36 |
| **Age, years** |  |  |  |  |  |  |  |  |  |  |
| 54 and below | 1(ref) | - | 1(ref) | - | 1(ref) | - | 1(ref) | - | 1(ref) | - |
| 55-64 | 1.14(0.98,1.32) | 0.09 | 2.12(1.53,2.96) | <0.001 | 3.35(1.66,6.77) | 0.00 | 3.39(1.95,5.91) | <0.001 | 2.83(1.99,4.04) | <0.001 |
| 65 and above | 1.28(1.04,1.58) | 0.02 | 2.48(1.56,3.95) | <0.001 | 4.01(2.26,7.13) | <0.001 | 4.25(2.69,6.74) | <0.001 | 3.12(2.26,4.32) | <0.001 |
| **Education** |  |  |  |  |  |  |  |  |  |  |
| Primary education and below | 1(ref) | - | 1(ref) | - | 1(ref) | - | 1(ref) | - | 1(ref) | - |
| College education | 1.41(1.14,1.75) | 0.00 | 1.72(1.08,2.74) | 0.02 | 1.95(1.04,3.65) | 0.04 | 1.85(1.33,2.57) | <0.001 | 1.83(1.16,2.88) | 0.01 |
| Secondary education | 1.11(0.89,1.37) | 0.36 | 1.62(1.04,2.52) | 0.03 | 1.72(1.06,2.78) | 0.03 | 1.97(1.22,3.19) | 0.01 | 2.50(1.61,3.88) | <0.001 |
| **Multivariable regressions with all variables** | | | | |  |  |  |  |  |  |
| **Sex** |  |  |  |  |  |  |  |  |  |  |
| Female | 1(ref) | - | 1(ref) | - | 1(ref) | - | 1(ref) | - | 1(ref) | - |
| Male | 1.29(1.11,1.50) | 0.00 | 1.42(0.81,2.46) | 0.22 | 1.36(0.74,2.48) | 0.32 | 1.15(0.62,2.13) | 0.65 | 1.04(0.58,1.86) | 0.89 |
| **Age, years** |  |  |  |  |  |  |  |  |  |  |
| 54 and below | 1(ref) | - | 1(ref) | - | 1(ref) | - | 1(ref) | - | 1(ref) | - |
| 55-64 | 1.17(0.99,1.39) | 0.07 | 2.22(1.50,3.27) | <0.001 | 3.88(1.60,9.40) | 0.00 | 3.75(1.97,7.14) | <0.001 | 3.14(1.98,4.98) | <0.001 |
| 65 and above | 1.26(1.00,1.59) | 0.04 | 2.45(1.50,4.02) | <0.001 | 4.43(2.29,8.55) | <0.001 | 4.49(2.93,6.87) | <0.001 | 3.36(2.48,4.55) | <0.001 |
| **Education** |  |  |  |  |  |  |  |  |  |  |
| Primary education and below | 1(ref) | - | 1(ref) | - | 1(ref) | - | 1(ref) | - | 1(ref) | - |
| College education | 1.18(1.00,1.38) | 0.05 | 1.49(0.80,2.76) | 0.21 | 2.01(1.23,3.29) | 0.01 | 1.98(1.12,3.51) | 0.02 | 2.12(0.96,4.68) | 0.06 |
| Secondary education | 1.04(0.85,1.26) | 0.73 | 1.52(0.84,2.75) | 0.17 | 1.65(0.92,2.97) | 0.09 | 1.95(0.94,4.06) | 0.07 | 2.51(1.15,5.47) | 0.02 |
| **Body-mass index group** | |  |  |  |  |  |  |  |  |  |
| Underweight | 1.14(0.85,1.53) | 0.40 | 0.88(0.51,1.52) | 0.64 | 0.59(0.33,1.06) | 0.08 | 0.92(0.49,1.73) | 0.79 | 1.09(0.51,2.34) | 0.82 |
| Healthy weight | 1(ref) | - | 1(ref) | - | 1(ref) | - | 1(ref) | - | 1(ref) | - |
| Overweight | 0.99(0.86,1.14) | 0.89 | 0.94(0.79,1.12) | 0.48 | 0.71(0.56,0.90) | 0.01 | 0.77(0.63,0.95) | 0.01 | 0.97(0.78,1.21) | 0.81 |
| Obese | 1.06(0.81,1.37) | 0.68 | 0.82(0.55,1.21) | 0.32 | 0.68(0.39,1.19) | 0.18 | 0.59(0.34,1.03) | 0.07 | 0.66(0.42,1.05) | 0.08 |
| **Tobacco smoking** | |  |  |  |  |  |  |  |  |  |
| Never smoked | 1(ref) | - | 1(ref) | - | 1(ref) | - | 1(ref) | - | 1(ref) | - |
| Ever smoked | 0.67(0.44,1.02) | 0.06 | 0.93(0.73,1.18) | 0.56 | 0.97(0.71,1.32) | 0.83 | 1.02(0.65,1.61) | 0.91 | 1.07(0.65,1.79) | 0.78 |
| **Urbanicity** |  |  |  |  |  |  |  |  |  |  |
| Rural townships | 1(ref) | - | 1(ref) | - | 1(ref) | - | 1(ref) | - | 1(ref) | - |
| Urban areas | 1.38(1.04,1.82) | 0.03 | 1.32(0.69,2.52) | 0.40 | 1.11(0.71,1.75) | 0.65 | 1.00 (0.57,1.75) | 0.99 | 1.08(0.45,2.56) | 0.87 |
| **mMRC = 1** | |  |  |  |  |  |  |  |  |  |
| **Univariable regression** | |  |  |  |  |  |  |  |  |  |
| **Sex** |  |  |  |  |  |  |  |  |  |  |
| Female | 1(ref) | - | 1(ref) | - | 1(ref) | - | 1(ref) | - | 1(ref) | - |
| Male | 1.16(1.08,1.26) | <0.001 | 1.30(1.09,1.55) | 0.00 | 1.36(1.10,1.68) | 0.01 | 1.30(1.00,1.70) | 0.05 | 1.32(1.02,1.71) | 0.03 |
| **Age, years** |  |  |  |  |  |  |  |  |  |  |
| 54 and below | 1(ref) | - | 1(ref) | - | 1(ref) | - | 1(ref) | - | 1(ref) | - |
| 55-64 | 1.11(0.99,1.24) | 0.08 | 1.71(1.46,1.99) | <0.001 | 1.56(1.25,1.94) | <0.001 | 1.35(1.04,1.76) | 0.02 | 1.29(1.02,1.62) | 0.04 |
| 65 and above | 1.09(0.89,1.33) | 0.41 | 1.71(1.44,2.03) | <0.001 | 1.55(1.12,2.14) | 0.01 | 1.17(0.72,1.89) | 0.54 | 1.16(0.68,1.97) | 0.59 |
| **Education** |  |  |  |  |  |  |  |  |  |  |
| Primary education and below | 1(ref) | - | 1(ref) | - | 1(ref) | - | 1(ref) | - | 1(ref) | - |
| Secondary education | 1.32(1.11,1.56) | 0.00 | 1.16(0.99,1.35) | 0.07 | 1.60(1.34,1.90) | <0.001 | 1.89(1.51,2.35) | <0.001 | 1.84(1.50,2.27) | <0.001 |
| College education | 1.12(1.00,1.25) | 0.04 | 1.14(0.97,1.33) | 0.11 | 1.55(1.34,1.79) | <0.001 | 1.96(1.56,2.47) | <0.001 | 1.95(1.48,2.55) | <0.001 |
| **Body-mass index group** | |  |  |  |  |  |  |  |  |  |
| Underweight | 1.07(0.93,1.23) | 0.34 | 1.24(0.98,1.56) | 0.07 | 1.16(0.84,1.59) | 0.37 | 0.96(0.65,1.40) | 0.83 | 0.97(0.65,1.45) | 0.89 |
| Healthy weight | 1(ref) | - | 1(ref) | - | 1(ref) | - | 1(ref) | - | 1(ref) | - |
| Overweight | 0.91(0.87,0.96) | <0.001 | 0.95(0.85,1.06) | 0.38 | 1.00(0.87,1.15) | 0.99 | 1.07(0.93,1.23) | 0.36 | 1.03(0.94,1.13) | 0.47 |
| Obese | 0.87(0.75,1.00) | 0.06 | 0.87(0.68,1.10) | 0.23 | 0.80(0.63,1.03) | 0.08 | 0.83(0.60,1.15) | 0.27 | 0.78(0.53,1.16) | 0.22 |
| **Tobacco smoking** | |  |  |  |  |  |  |  |  |  |
| Never smoked | 1(ref) | - | 1(ref) | - | 1(ref) | - | 1(ref) | - | 1(ref) | - |
| Ever smoked | 0.90(0.64,1.26) | 0.53 | 1.22(1.00,1.50) | 0.05 | 1.22(0.95,1.56) | 0.12 | 1.26(0.92,1.74) | 0.14 | 1.27(0.93,1.73) | 0.14 |
| **Urbanicity** |  |  |  |  |  |  |  |  |  |  |
| Rural townships | 1(ref) | - | 1(ref) | - | 1(ref) | - | 1(ref) | - | 1(ref) | - |
| Urban areas | 1.27(1.03,1.56) | 0.03 | 1.08(0.85,1.38) | 0.53 | 1.35(1.01,1.81) | 0.04 | 1.68(1.28,2.21) | <0.001 | 1.71(1.28,2.30) | <0.001 |
| **Multivariable regressions with age group, sex, and education** | | | | |  |  |  |  |  |  |
| **Sex** |  |  |  |  |  |  |  |  |  |  |
| Female | 1(ref) | - | 1(ref) | - | 1(ref) | - | 1(ref) | - | 1(ref) | - |
| Male | 1.14(1.05,1.24) | 0.00 | 1.25(1.06,1.48) | 0.01 | 1.27(1.02,1.58) | 0.03 | 1.19(0.92,1.55) | 0.19 | 1.21(0.94,1.57) | 0.15 |
| **Age, years** |  |  |  |  |  |  |  |  |  |  |
| 54 and below | 1(ref) | - | 1(ref) | - | 1(ref) | - | 1(ref) | - | 1(ref) | - |
| 55-64 | 1.13(1.01,1.26) | 0.03 | 1.73(1.48,2.04) | <0.001 | 1.61(1.30,2.00) | <0.001 | 1.42(1.10,1.84) | 0.01 | 1.34(1.06,1.69) | 0.01 |
| 65 and above | 1.13(0.94,1.36) | 0.21 | 1.76(1.47,2.10) | <0.001 | 1.69(1.24,2.30) | 0.00 | 1.31(0.82,2.10) | 0.27 | 1.28(0.76,2.18) | 0.35 |
| **Education** |  |  |  |  |  |  |  |  |  |  |
| Primary education and below | 1(ref) | - | 1(ref) | - | 1(ref) | - | 1(ref) | - | 1(ref) | - |
| College education | 1.33(1.16,1.54) | <0.001 | 1.25(1.07,1.47) | 0.01 | 1.70(1.46,1.99) | <0.001 | 1.92(1.57,2.34) | <0.001 | 1.86(1.54,2.25) | <0.001 |
| Secondary education | 1.12(1.02,1.23) | 0.02 | 1.16(0.99,1.36) | 0.07 | 1.58(1.37,1.81) | <0.001 | 1.94(1.57,2.40) | <0.001 | 1.93(1.49,2.49) | <0.001 |
| **Multivariable regressions with all variables** | | | | |  |  |  |  |  |  |
| **Sex** |  |  |  |  |  |  |  |  |  |  |
| Female | 1(ref) | - | 1(ref) | - | 1(ref) | - | 1(ref) | - | 1(ref) | - |
| Male | 1.20zhi(1.09,1.32) | <0.001 | 1.19(1.06,1.34) | 0.00 | 1.36(1.07,1.73) | 0.01 | 1.30(0.97,1.73) | 0.08 | 1.33(1.05,1.70) | 0.02 |
| **Age, years** |  |  |  |  |  |  |  |  |  |  |
| 54 and below | 1(ref) | - | 1(ref) | - | 1(ref) | - | 1(ref) | - | 1(ref) | - |
| 55-64 | 1.17(1.05,1.30) | 0.00 | 1.84(1.48,2.30) | <0.001 | 1.71(1.35,2.16) | <0.001 | 1.46(1.08,1.98) | 0.01 | 1.42(1.06,1.89) | 0.02 |
| 65 and above | 1.15(0.94,1.40) | 0.18 | 1.82(1.48,2.24) | <0.001 | 1.78(1.33,2.37) | <0.001 | 1.35(0.81,2.23) | 0.24 | 1.35(0.77,2.36) | 0.30 |
| **Education** |  |  |  |  |  |  |  |  |  |  |
| Primary education and below | 1(ref) | - | 1(ref) | - | 1(ref) | - | 1(ref) | - | 1(ref) | - |
| College education | 1.26(1.11,1.44) | 0.00 | 1.27(1.09,1.47) | 0.00 | 1.67(1.45,1.93) | <0.001 | 1.76(1.38,2.25) | <0.001 | 1.70(1.36,2.12) | <0.001 |
| Secondary education | 1.08(0.99,1.18) | 0.10 | 1.14(1.00,1.30) | 0.05 | 1.50(1.30,1.75) | <0.001 | 1.74(1.44,2.09) | <0.001 | 1.68(1.39,2.05) | <0.001 |
| **Body-mass index group** | |  |  |  |  |  |  |  |  |  |
| Underweight | 1.09(0.93,1.27) | 0.30 | 1.21(0.94,1.56) | 0.14 | 1.12(0.80,1.56) | 0.52 | 0.97(0.67,1.41) | 0.88 | 0.96(0.65,1.43) | 0.86 |
| Healthy weight | 1(ref) | - | 1(ref) | - | 1(ref) | - | 1(ref) | - | 1(ref) | - |
| Overweight | 0.90(0.84,0.96) | 0.00 | 0.96(0.88,1.05) | 0.36 | 0.95(0.82,1.09) | 0.46 | 0.99(0.86,1.14) | 0.89 | 0.95(0.83,1.10) | 0.50 |
| Obese | 0.87(0.75,1.00) | 0.05 | 0.85(0.66,1.10) | 0.22 | 0.79(0.61,1.03) | 0.08 | 0.80(0.56,1.15) | 0.23 | 0.77(0.50,1.19) | 0.24 |
| **Tobacco smoking** | |  |  |  |  |  |  |  |  |  |
| Never smoked | 1(ref) | - | 1(ref) | - | 1(ref) | - | 1(ref) | - | 1(ref) | - |
| Ever smoked | 0.80(0.57,1.12) | 0.19 | 1.05(0.88,1.27) | 0.58 | 0.96(0.69,1.33) | 0.80 | 1.00(0.65,1.55) | 0.99 | 0.99(0.66,1.49) | 0.97 |
| **Urbanicity** |  |  |  |  |  |  |  |  |  |  |
| Rural townships | 1(ref) | - | 1(ref) | - | 1(ref) | - | 1(ref) | - | 1(ref) | - |
| Urban areas | 1.21(0.99,1.48) | 0.06 | 1.05(0.82,1.34) | 0.71 | 1.11(0.82,1.49) | 0.50 | 1.21(0.96,1.54) | 0.11 | 1.20(0.95,1.52) | 0.13 |
| **mMRC = 2** | |  |  |  |  |  |  |  |  |  |
| **Univariable regression** | |  |  |  |  |  |  |  |  |  |
| **Sex** |  |  |  |  |  |  |  |  |  |  |
| Female | 1(ref) | - | 1(ref) | - | 1(ref) | - | 1(ref) | - | 1(ref) | - |
| Male | 1.13(1.05,1.21) | 0.00 | 1.33(1.14,1.56) | <0.001 | 1.72(1.45,2.05) | <0.001 | 1.53(1.34,1.75) | <0.001 | 1.54(1.35,1.75) | <0.001 |
| **Age, years** |  |  |  |  |  |  |  |  |  |  |
| 54 and below | 1(ref) | - | 1(ref) | - | 1(ref) | - | 1(ref) | - | 1(ref) | - |
| 55-64 | 0.96(0.91,1.02) | 0.23 | 1.31(1.10,1.56) | 0.00 | 1.11(0.84,1.47) | 0.46 | 1.28(0.92,1.78) | 0.14 | 1.25(0.89,1.75) | 0.20 |
| 65 and above | 1.01(0.93,1.08) | 0.88 | 1.47(1.19,1.82) | <0.001 | 1.19(0.85,1.66) | 0.31 | 1.14(0.75,1.71) | 0.54 | 1.12(0.73,1.73) | 0.60 |
| **Education** |  |  |  |  |  |  |  |  |  |  |
| Primary education and below | 1(ref) | - | 1(ref) | - | 1(ref) | - | 1(ref) | - | 1(ref) | - |
| Secondary education | 1.17(1.07,1.29) | 0.00 | 1.37(1.06,1.77) | 0.02 | 1.72(1.26,2.36) | 0.00 | 1.86(1.44,2.40) | <0.001 | 1.76(1.40,2.23) | <0.001 |
| College education | 1.11(1.00,1.22) | 0.04 | 1.19(1.02,1.38) | 0.02 | 1.39(1.06,1.81) | 0.01 | 1.80(1.45,2.24) | <0.001 | 1.86(1.48,2.33) | <0.001 |
| **Body-mass index group** | |  |  |  |  |  |  |  |  |  |
| Underweight | 1.04(0.94,1.14) | 0.47 | 1.22(1.05,1.42) | 0.01 | 1.02(0.83,1.25) | 0.85 | 0.94(0.70,1.27) | 0.70 | 1.04(0.76,1.41) | 0.82 |
| Healthy weight | 1(ref) | - | 1(ref) | - | 1(ref) | - | 1(ref) | - | 1(ref) | - |
| Overweight | 0.97(0.91,1.04) | 0.41 | 1.00(0.89,1.11) | 0.94 | 1.03(0.87,1.22) | 0.73 | 1.06(0.87,1.29) | 0.56 | 1.04(0.86,1.26) | 0.67 |
| Obese | 0.92(0.84,1.01) | 0.09 | 0.90(0.72,1.11) | 0.31 | 0.93(0.68,1.27) | 0.64 | 0.87(0.60,1.26) | 0.46 | 0.83(0.59,1.18) | 0.30 |
| **Tobacco smoking** | |  |  |  |  |  |  |  |  |  |
| Never smoked | 1(ref) | - | 1(ref) | - | 1(ref) | - | 1(ref) | - | 1(ref) | - |
| Ever smoked | 0.95(0.75,1.20) | 0.65 | 1.06(0.73,1.55) | 0.76 | 1.40(1.09,1.81) | 0.01 | 1.24(1.03,1.49) | 0.03 | 1.27(1.08,1.50) | 0.00 |
| **Urbanicity** |  |  |  |  |  |  |  |  |  |  |
| Rural townships | 1(ref) | - | 1(ref) | - | 1(ref) | - | 1(ref) | - | 1(ref) | - |
| Urban areas | 1.18(1.08,1.28) | <0.001 | 1.09(0.95,1.25) | 0.22 | 1.55(1.25,1.92) | <0.001 | 1.93(1.39,2.66) | <0.001 | 2.10(1.44,3.05) | <0.001 |
| **Multivariable regressions with age group, sex, and education** | | | | |  |  |  |  |  |  |
| **Sex** |  |  |  |  |  |  |  |  |  |  |
| Female | 1(ref) | - | 1(ref) | - | 1(ref) | - | 1(ref) | - | 1(ref) | - |
| Male | 1.11(1.05,1.18) | 0.00 | 1.28(1.10,1.50) | 0.00 | 1.68(1.43,1.97) | <0.001 | 1.47(1.30,1.67) | <0.001 | 1.47(1.32,1.64) | <0.001 |
| **Age, years** |  |  |  |  |  |  |  |  |  |  |
| 54 and below | 1(ref) | - | 1(ref) | - | 1(ref) | - | 1(ref) | - | 1(ref) | - |
| 55-64 | 0.96(0.90,1.03) | 0.28 | 1.29(1.05,1.59) | 0.02 | 1.12(0.83,1.50) | 0.46 | 1.31(0.93,1.85) | 0.12 | 1.26(0.91,1.74) | 0.16 |
| 65 and above | 1.02(0.94,1.11) | 0.55 | 1.54(1.21,1.97) | <0.001 | 1.26(0.90,1.76) | 0.19 | 1.26(0.84,1.88) | 0.26 | 1.24(0.84,1.84) | 0.28 |
| **Education** |  |  |  |  |  |  |  |  |  |  |
| Primary education and below | 1(ref) | - | 1(ref) | - | 1(ref) | - | 1(ref) | - | 1(ref) | - |
| College education | 1.17(1.06,1.28) | 0.00 | 1.45(1.10,1.90) | 0.01 | 1.70(1.21,2.37) | 0.00 | 1.83(1.37,2.44) | <0.001 | 1.73(1.32,2.26) | <0.001 |
| Secondary education | 1.10(1.00,1.20) | 0.04 | 1.20(1.04,1.39) | 0.01 | 1.33(1.06,1.68) | 0.01 | 1.73(1.39,2.16) | <0.001 | 1.78(1.40,2.27) | <0.001 |
| **Multivariable regressions with all variables** | | | | |  |  |  |  |  |  |
| **Sex** |  |  |  |  |  |  |  |  |  |  |
| Female | 1(ref) | - | 1(ref) | - | 1(ref) | - | 1(ref) | - | 1(ref) | - |
| Male | 1.13(1.05,1.20) | <0.001 | 1.23(1.07,1.41) | 0.00 | 1.67(1.33,2.10) | <0.001 | 1.47(1.12,1.92) | 0.01 | 1.45(1.05,2.00) | 0.03 |
| **Age, years** |  |  |  |  |  |  |  |  |  |  |
| 54 and below | 1(ref) | - | 1(ref) | - | 1(ref) | - | 1(ref) | - | 1(ref) | - |
| 55-64 | 0.98(0.89,1.07) | 0.62 | 1.36(1.07,1.73) | 0.01 | 1.21(0.93,1.57) | 0.17 | 1.39(1.08,1.79) | 0.01 | 1.23(0.91,1.67) | 0.18 |
| 65 and above | 1.04(0.92,1.18) | 0.51 | 1.59(1.21,2.09) | 0.00 | 1.32(0.98,1.76) | 0.07 | 1.33(1.00,1.76) | 0.05 | 1.22(0.87,1.71) | 0.25 |
| **Education** |  |  |  |  |  |  |  |  |  |  |
| Primary education and below | 1(ref) | - | 1(ref) | - | 1(ref) | - | 1(ref) | - | 1(ref) | - |
| College education | 1.17(1.06,1.30) | 0.00 | 1.45(1.12,1.88) | 0.01 | 1.62(1.14,2.29) | 0.01 | 1.67(1.20,2.31) | 0.00 | 1.46(1.07,2.00) | 0.02 |
| Secondary education | 1.06(0.96,1.16) | 0.23 | 1.22(1.04,1.43) | 0.02 | 1.27(0.96,1.68) | 0.09 | 1.62(1.19,2.20) | 0.00 | 1.58(1.11,2.24) | 0.01 |
| **Body-mass index group** | |  |  |  |  |  |  |  |  |  |
| Underweight | 1.07(0.99,1.17) | 0.10 | 1.23(1.05,1.43) | 0.01 | 1.01(0.79,1.28) | 0.95 | 0.88(0.66,1dshai.16) | 0.36 | 0.95(0.71,1.27) | 0.74 |
| Healthy weight | 1(ref) | - | 1(ref) | - | 1(ref) | - | 1(ref) | - | 1(ref) | - |
| Overweight | 1.00(0.94,1.06) | 0.96 | 1.02(0.91,1.15) | 0.68 | 1.02(0.86,1.21) | 0.86 | 1.01(0.81,1.28) | 0.90 | 0.95(0.74,1.23) | 0.70 |
| Obese | 0.90(0.80,1.02) | 0.09 | 0.86(0.70,1.06) | 0.16 | 0.89(0.67,1.18) | 0.42 | 0.78(0.48,1.25) | 0.29 | 0.77(0.46,1.30) | 0.33 |
| **Tobacco smoking** | |  |  |  |  |  |  |  |  |  |
| Never smoked | 1(ref) | - | 1(ref) | - | 1(ref) | - | 1(ref) | - | 1(ref) | - |
| Ever smoked | 0.87(0.68,1.11) | 0.26 | 0.91(0.63,1.32) | 0.62 | 1.05(0.83,1.32) | 0.70 | 1.01(0.81,1.27) | 0.93 | 1.06(0.79,1.43) | 0.69 |
| **Urbanicity** |  |  |  |  |  |  |  |  |  |  |
| Rural townships | 1(ref) | - | 1(ref) | - | 1(ref) | - | 1(ref) | - | 1(ref) | - |
| Urban areas | 1.11(1.04,1.19) | 0.00 | 1.01(0.88,1.16) | 0.89 | 1.42(1.14,1.75) | 0.00 | 1.48(1.07,2.06) | 0.02 | 1.57(1.07,2.31) | 0.02 |
| **mMRC = 3** | |  |  |  |  |  |  |  |  |  |
| **Univariable regression** | |  |  |  |  |  |  |  |  |  |
| **Sex** |  |  |  |  |  |  |  |  |  |  |
| Female | 1(ref) | - | 1(ref) | - | 1(ref) | - | 1(ref) | - | 1(ref) | - |
| Male | 1.10(1.03,1.17) | 0.00 | 1.20(1.11,1.30) | <0.001 | 1.30(1.17,1.44) | <0.001 | 1.42(1.14,1.79) | 0.00 | 1.47(1.17,1.84) | 0.00 |
| **Age, years** |  |  |  |  |  |  |  |  |  |  |
| 54 and below | 1(ref) | - | 1(ref) | - | 1(ref) | - | 1(ref) | - | 1(ref) | - |
| 55-64 | 1.13(1.03,1.25) | 0.01 | 1.40(1.10,1.78) | 0.01 | 1.19(0.78,1.83) | 0.42 | 1.32(0.60,2.91) | 0.49 | 1.06(0.51,2.19) | 0.88 |
| 65 and above | 1.01(0.92,1.11) | 0.81 | 1.32(1.02,1.71) | 0.03 | 1.16(0.78,1.74) | 0.46 | 1.34(0.51,3.55) | 0.55 | 1.19(0.43,3.27) | 0.74 |
| **Education** |  |  |  |  |  |  |  |  |  |  |
| Primary education and below | 1(ref) | - | 1(ref) | - | 1(ref) | - | 1(ref) | - | 1(ref) | - |
| Secondary education | 1.38(1.07,1.77) | 0.01 | 1.35(0.89,2.05) | 0.15 | 1.33(0.92,1.93) | 0.13 | 0.73(0.32,1.70) | 0.47 | 0.81(0.37,1.81) | 0.62 |
| College education | 1.25(1.10,1.42) | 0.00 | 1.27(1.03,1.55) | 0.02 | 1.72(1.37,2.16) | <0.001 | 1.84(1.37,2.48) | <0.001 | 2.08(1.49,2.90) | <0.001 |
| **Body-mass index group** | |  |  |  |  |  |  |  |  |  |
| Underweight | 1.08(0.98,1.19) | 0.10 | 1.27(1.05,1.54) | 0.01 | 1.07(0.90,1.28) | 0.45 | 0.97(0.58,1.62) | 0.89 | 0.80(0.45,1.41) | 0.43 |
| Healthy weight | 1(ref) | - | 1(ref) | - | 1(ref) | - | 1(ref) | - | 1(ref) | - |
| Overweight | 0.97(0.92,1.01) | 0.17 | 0.96(0.87,1.07) | 0.49 | 1.10(0.95,1.29) | 0.20 | 1.16(0.90,1.50) | 0.25 | 1.23(0.97,1.58) | 0.09 |
| Obese | 0.98(0.87,1.11) | 0.76 | 0.90(0.73,1.10) | 0.29 | 0.89(0.65,1.22) | 0.48 | 0.86(0.57,1.29) | 0.47 | 1.01(0.71,1.44) | 0.94 |
| **Tobacco smoking** | |  |  |  |  |  |  |  |  |  |
| Never smoked | 1(ref) | - | 1(ref) | - | 1(ref) | - | 1(ref) | - | 1(ref) | - |
| Ever smoked | 1.05(0.93,1.18) | 0.44 | 1.20(1.06,1.36) | 0.01 | 1.30(1.05,1.60) | 0.02 | 1.36(0.98,1.88) | 0.06 | 1.41(0.91,2.17) | 0.12 |
| **Urbanicity** |  |  |  |  |  |  |  |  |  |  |
| Rural townships | 1(ref) | - | 1(ref) | - | 1(ref) | - | 1(ref) | - | 1(ref) | - |
| Urban areas | 1.18(1.07,1.30) | 0.00 | 1.19(1.06,1.32) | 0.00 | 1.56(1.15,2.11) | 0.01 | 2.55(1.79,3.65) | <0.001 | 3.26(2.27,4.69) | <0.001 |
| **Multivariable regressions with age group, sex, and education** | | | | |  |  |  |  |  |  |
| **Sex** |  |  |  |  |  |  |  |  |  |  |
| Female | 1(ref) | - | 1(ref) | - | 1(ref) | - | 1(ref) | - | 1(ref) | - |
| Male | 1.06(1.00,1.12) | 0.04 | 1.15(1.06,1.26) | 0.00 | 1.20(1.09,1.33) | <0.001 | 1.33(1.05,1.69) | 0.02 | 1.36(1.07,1.72) | 0.01 |
| **Age, years** |  |  |  |  |  |  |  |  |  |  |
| 54 and below | 1(ref) | - | 1(ref) | - | 1(ref) | - | 1(ref) | - | 1(ref) | - |
| 55-64 | 1.18(1.05,1.33) | 0.01 | 1.45(1.12,1.88) | 0.01 | 1.27(0.81,1.99) | 0.29 | 1.42(0.68,2.96) | 0.36 | 1.12(0.59,2.15) | 0.72 |
| 65 and above | 1.09(0.99,1.21) | 0.07 | 1.43(1.11,1.84) | 0.01 | 1.30(0.91,1.87) | 0.14 | 1.50(0.63,3.56) | 0.36 | 1.32(0.55,3.19) | 0.53 |
| **Education** |  |  |  |  |  |  |  |  |  |  |
| Primary education and below | 1(ref) | - | 1(ref) | - | 1(ref) | - | 1(ref | - | 1(ref) | - |
| College education | 1.37(1.06,1.77) | 0.02 | 1.35(0.90,2.04) | 0.15 | 1.31(0.91,1.89) | 0.14 | 0.72(0.32,1.61) | 0.42 | 0.80(0.38,1.69) | 0.56 |
| Secondary education | 1.24(1.09,1.41) | 0.00 | 1.25(1.02,1.54) | 0.03 | 1.68(1.35,2.10) | <0.001 | 1.78(1.33,2.38) | <0.001 | 2.01(1.46,2.76) | <0.001 |
| **Multivariable regressions with all variables** | | | | |  |  |  |  |  |  |
| **Sex** |  |  |  |  |  |  |  |  |  |  |
| Female | 1(ref) | - | 1(ref) | - | 1(ref) | - | 1(ref) | - | 1(ref) | - |
| Male | 1.05(0.92,1.20) | 0.43 | 1.05(0.91,1.21) | 0.48 | 1.05(0.82,1.35) | 0.69 | 1.51(1.14,2.00) | 0.00 | 1.56(1.15,2.12) | 0.01 |
| **Age, years** |  |  |  |  |  |  |  |  |  |  |
| 54 and below | 1(ref) | - | 1(ref) | - | 1(ref) | - | 1(ref) | - | 1(ref) | - |
| 55-64 | 1.17(1.03,1.33) | 0.02 | 1.48(1.15,1.91) | 0.00 | 1.42(0.85,2.37) | 0.18 | 1.84(0.56,6.07) | 0.32 | 1.35(0.47,3.84) | 0.57 |
| 65 and above | 1.06(0.94,1.21) | 0.33 | 1.41(1.11,1.77) | 0.00 | 1.38(0.91,2.11) | 0.13 | 1.62(0.46,5.72) | 0.45 | 1.38(0.42,4.50) | 0.59 |
| **Education** |  |  |  |  |  |  |  |  |  |  |
| Primary education and below | 1(ref) | - | 1(ref) | - | 1(ref) | - | 1(ref) | - | 1(ref) | - |
| College education | 1.36(1.01,1.83) | 0.04 | 1.36(0.84,2.21) | 0.21 | 1.27(0.88,1.84) | 0.21 | 0.50(0.21,1.19) | 0.12 | 0.50(0.22,1.15) | 0.10 |
| Secondary education | 1.24(1.05,1.45) | 0.01 | 1.26(0.98,1.61) | 0.07 | 1.59(1.22,2.07) | 0.00 | 1.37(1.02,1.84) | 0.03 | 1.44(1.00,2.06) | 0.05 |
| **Body-mass index group** | |  |  |  |  |  |  |  |  |  |
| Underweight | 1.10(1.01,1.20) | 0.04 | 1.28(1.09,1.50) | 0.00 | 1.08(0.92,1.27) | 0.32 | 0.96(0.59,1.57) | 0.87 | 0.79(0.46,1.33) | 0.37 |
| Healthy weight | 1(ref) | - | 1(ref) | - | 1(ref) | - | 1(ref) | - | 1(ref) | - |
| Overweight | 0.94(0.89,1.00) | 0.05 | 0.97(0.87,1.07) | 0.53 | 1.05(0.93,1.20) | 0.42 | 0.99(0.79,1.23) | 0.91 | 1.08(0.90,1.29) | 0.42 |
| Obese | 1.00(0.89,1.13) | 0.99 | 0.99(0.82,1.20) | 0.93 | 0.97(0.62,1.50) | 0.88 | 0.73(0.46,1.19) | 0.21 | 0.94(0.56,1.56) | 0.80 |
| **Tobacco smoking** | |  |  |  |  |  |  |  |  |  |
| Never smoked | 1(ref) | - | 1(ref) | - | 1(ref) | - | 1(ref) | - | 1(ref) | - |
| Ever smoked | 0.99(0.86,1.13) | 0.86 | 1.11(0.96,1.29) | 0.15 | 1.21(0.93,1.57) | 0.15 | 1.07(0.71,1.62) | 0.73 | 1.11(0.67,1.86) | 0.69 |
| **Urbanicity** |  |  |  |  |  |  |  |  |  |  |
| Rural townships | 1(ref) | - | 1(ref) | - | 1(ref) | - | 1(ref) | - | 1(ref) | - |
| Urban areas | 1.06(0.93,1.21) | 0.42 | 1.04(0.86,1.27) | 0.67 | 1.19(0.85,1.66) | 0.31 | 1.93(1.35,2.78) | <0.001 | 2.45(1.62,3.70) | <0.001 |
| **mMRC = 4** | |  |  |  |  |  |  |  |  |  |
| **Univariable regression** | |  |  |  |  |  |  |  |  |  |
| **Sex** |  |  |  |  |  |  |  |  |  |  |
| Female | 1(ref) | - | 1(ref) | - | 1(ref) | - | 1(ref) | - | 1(ref) | - |
| Male | 1.22(1.03,1.45) | 0.02 | 1.20(0.96,1.50) | 0.11 | 1.14(0.77,1.70) | 0.51 | 0.46(0.23,0.93) | 0.03 | 0.45(0.24,0.85) | 0.01 |
| **Age, years** |  |  |  |  |  |  |  |  |  |  |
| 54 and below | 1(ref) | - | 1(ref) | - | 1(ref) | - | 1(ref) | - | 1(ref) | - |
| 55-64 | 1.26(0.84,1.89) | 0.26 | 1.28(0.68,2.39) | 0.44 | 1.25(0.61,2.56) | 0.55 | 2065534.40(522944.20,8158485.10) | <0.001 | 18951521.00(7291139.50,49259808.00) | <0.001 |
| 65 and above | 1.10(0.78,1.53) | 0.59 | 1.17(0.67,2.03) | 0.59 | 1.14(0.59,2.18) | 0.70 | 2514125.50(1033092.40,6118355.80) | <0.001 | 22803687.00(12132978.00,42859069.00) | <0.001 |
| **Education** |  |  |  |  |  |  |  |  |  |  |
| Primary education and below | 1(ref) | - | 1(ref) | - | 1(ref) | - | 1(ref) | - | 1(ref) | - |
| Secondary education | 1.41(1.04,1.90) | 0.03 | 1.51(1.00,2.28) | 0.05 | 1.85(0.86,3.99) | 0.11 | 1.79(0.13,24.22) | 0.66 | 2.13(0.14,31.47) | 0.58 |
| College education | 1.30(1.10,1.53) | 0.00 | 1.47(1.17,1.85) | 0.00 | 2.07(1.36,3.14) | 0.00 | 3.21(1.06,9.68) | 0.04 | 3.06(1.00,9.38) | 0.05 |
| **Body-mass index group** | |  |  |  |  |  |  |  |  |  |
| Underweight | 1.00(0.82,1.21) | 0.97 | 1.08(0.76,1.53) | 0.66 | 0.96(0.60,1.53) | 0.85 | 0.72(0.28,1.86) | 0.50 | 0.75(0.21,2.68) | 0.66 |
| Healthy weight | 1(ref) | - | 1(ref) | - | 1(ref) | - | 1(ref) | - | 1(ref) | - |
| Overweight | 1.08(0.95,1.23) | 0.22 | 1.19(1.01,1.41) | 0.04 | 1.04(0.70,1.54) | 0.85 | 0.94(0.41,2.12) | 0.88 | 1.51(0.83,2.76) | 0.18 |
| Obese | 0.84(0.61,1.14) | 0.26 | 0.70(0.38,1.29) | 0.26 | 0.75(0.34,1.65) | 0.47 | 1.03(0.20,5.15) | 0.97 | 1.75(0.37,8.22) | 0.48 |
| **Tobacco smoking** | |  |  |  |  |  |  |  |  |  |
| Never smoked | 1(ref) | - | 1(ref) | - | 1(ref) | - | 1(ref) | - | 1(ref) | - |
| Ever smoked | 1.09(0.88,1.34) | 0.43 | 1.19(0.85,1.66) | 0.32 | 0.89(0.69,1.16) | 0.40 | 0.49(0.18,1.36) | 0.17 | 0.43(0.19,0.97) | 0.04 |
| **Urbanicity** |  |  |  |  |  |  |  |  |  |  |
| Rural townships | 1(ref) | - | 1(ref) | - | 1(ref) | - | 1(ref) | - | 1(ref) | - |
| Urban areas | 1.12(0.88,1.43) | 0.34 | 1.32(0.93,1.88) | 0.12 | 1.43(0.70,2.92) | 0.33 | 4.49(0.89,22.80) | 0.07 | 3.66(0.67,19.98) | 0.13 |
| **Multivariable regressions with age group, sex, and education** | | | | |  |  |  |  |  |  |
| **Sex** |  |  |  |  |  |  |  |  |  |  |
| Female | 1(ref) | - | 1(ref) | - | 1(ref) | - | 1(ref) | - | 1(ref) | - |
| Male | 1.19(1.02,1.39) | 0.03 | 1.14(0.91,1.42) | 0.26 | 1.01(0.66,1.56) | 0.94 | 0.36(0.20,0.66) | 0.00 | 0.34(0.19,0.61) | <0.001 |
| **Age, years** |  |  |  |  |  |  |  |  |  |  |
| 54 and below | 1(ref) | - | 1(ref) | - | 1(ref) | - | 1(ref) | - | 1(ref) | - |
| 55-64 | 1.22(0.88,1.68) | 0.23 | 1.28(0.65,2.51) | 0.47 | 1.39(0.63,3.05) | 0.41 | 3690022.80(1080947.00,12596610.00) | <0.001 | 2174184.60(500210.41,9450180.60) | <0.001 |
| 65 and above | 1.09(0.86,1.39) | 0.47 | 1.23(0.71,2.12) | 0.46 | 1.36(0.69,2.68) | 0.38 | 5411846.70(2210181.70,13251437.00) | <0.001 | 3049341.70(1077716.30,8627952.60) | <0.001 |
| **Education** |  |  |  |  |  |  |  |  |  |  |
| Primary education and below | 1(ref) | - | 1(ref) | - | 1(ref) | - | 1(ref) | - | 1(ref) | - |
| College education | 1.39(1.05,1.84) | 0.02 | 1.54(1.05,2.27) | 0.03 | 1.93(0.90,4.12) | 0.09 | 1.85(0.10,33.37) | 0.68 | 2.38(0.11,51.13) | 0.58 |
| Secondary education | 1.24(1.09,1.42) | 0.00 | 1.44(1.16,1.78) | 0.00 | 2.09(1.30,3.36) | 0.00 | 4.08(1.50,11.15) | 0.01 | 4.11(1.49,11.31) | 0.01 |
| **Multivariable regressions with all variables** | | | | |  |  |  |  |  |  |
| **Sex** |  |  |  |  |  |  |  |  |  |  |
| Female | 1(ref) | - | 1(ref) | - | 1(ref) | - | 1(ref) | - | 1(ref) | - |
| Male | 1.19(0.95,1.49) | 0.12 | 0.90(0.68,1.20) | 0.48 | 0.88(0.55,1.41) | 0.59 | 0.27(0.10,0.76) | 0.01 | 0.28(0.10,0.81) | 0.02 |
| **Age, years** |  |  |  |  |  |  |  |  |  |  |
| 54 and below | 1(ref) | - | 1(ref) | - | 1(ref) | - | 1(ref) | - | 1(ref) | - |
| 55-64 | 1.15(0.82,1.60) | 0.42 | 1.10(0.57,2.14) | 0.77 | 1.33(0.52,3.44) | 0.55 | 6636609.80(1422435.90,30964201.00) | <0.001 | 2024779.80(215723.77,19004551.00) | <0.001 |
| 65 and above | 1.01(0.81,1.26) | 0.95 | 1.01(0.59,1.74) | 0.97 | 1.18(0.57,2.44) | 0.66 | 7138544.60(2492360.70,20446005.00) | <0.001 | 2526028.90(530487.79,12028216.00) | <0.001 |
| **Education** |  |  |  |  |  |  |  |  |  |  |
| Primary education and below | 1(ref) | - | 1(ref) | - | 1(ref) | - | 1(ref) | - | 1(ref) | - |
| College education | 1.36(1.02,1.83) | 0.04 | 1.41(0.99,2.01) | 0.05 | 1.99(0.90,4.37) | 0.09 | 1.80(0.14,23.06) | 0.65 | 2.74(0.31,24.49) | 0.37 |
| Secondary education | 1.24(1.09,1.41) | 0.00 | 1.32(1.05,1.68) | 0.02 | 2.05(1.21,3.46) | 0.01 | 2.74(0.66,11.44) | 0.17 | 3.11(0.60,15.96) | 0.17 |
| **Body-mass index group** | |  |  |  |  |  |  |  |  |  |
| Underweight | 1.03(0.85,1.25) | 0.77 | 1.04(0.70,1.53) | 0.86 | 0.94(0.57,1.53) | 0.79 | 0.73(0.27,1.97) | 0.54 | 0.67(0.12,3.65) | 0.65 |
| Healthy weight | 1(ref) | - | 1(ref) | - | 1(ref) | - | 1(ref) | - | 1(ref) | - |
| Overweight | 1.13(0.93,1.37) | 0.21 | 1.21(0.91,1.62) | 0.20 | 1.08(0.68,1.73) | 0.74 | 1.43(0.62,3.32) | 0.41 | 1.86(0.96,3.60) | 0.07 |
| Obese | 0.98(0.64,1.50) | 0.92 | 0.71(0.34,1.49) | 0.37 | 0.84(0.33,2.19) | 0.73 | 0.84(0.08,8.45) | 0.89 | 1.23(0.12,13.00) | 0.86 |
| **Tobacco smoking** | |  |  |  |  |  |  |  |  |  |
| Never smoked | 1(ref) | - | 1(ref) | - | 1(ref) | - | 1(ref) | - | 1(ref) | - |
| Ever smoked | 0.98(0.75,1.29) | 0.91 | 1.22(0.80,1.85) | 0.37 | 0.92(0.67,1.27) | 0.62 | 1.17(0.54,2.54) | 0.68 | 0.89(0.57,1.37) | 0.60 |
| **Urbanicity** |  |  |  |  |  |  |  |  |  |  |
| Rural townships | 1(ref) | - | 1(ref) | - | 1(ref) | - | 1(ref) | - | 1(ref) | - |
| Urban areas | 1.03(0.82,1.30) | 0.81 | 1.20(0.83,1.75) | 0.34 | 1.17(0.61,2.25) | 0.63 | 3.25(0.85,12.34) | 0.08 | 2.55(0.57,11.47) | 0.22 |

Table S6. Interaction analysis of each cascade stage. Note: “GDP” refers to the log-transformed per-capita GDP from 2014 to 2019. RRs for female, age group, body-mass index, and tobacco smoking on Controlled 2 cannot be reached in univariable regressions, so we substitute these results with ORs. COPD=chronic obstructive pulmonary disease, Controlled 1 = Controlled (mild or no exacerbations), Controlled 2 = Controlled (no exacerbations). Regions with sample size under 100 were not included in this analysis.

| **Variable** | **Tested-Model 1** | | **Tested-Model 2** | | **Tested-Model 3** | | **Tested-Model 4** | | **Tested-Model 5** | | **Tested-Model 6** | |
| --- | --- | --- | --- | --- | --- | --- | --- | --- | --- | --- | --- | --- |
|  | **RR (95% CI)** | **p-value** | **RR (95% CI)** | **p-value** | **RR (95% CI)** | **p-value** | **RR (95% CI)** | **p-value** | **RR (95% CI)** | **p-value** | **RR (95% CI)** | **p-value** |
| **Sex*GDP** | |  |  |  |  |  |  |  |  |  |  |  |
| Female*GDP | 1 (Ref) |  |  |  |  |  |  |  |  |  |  |  |
| Male*GDP | 0.98 (0.89, 0.98) | 0.66 |  |  |  |  |  |  |  |  |  |  |
| **Age, years*GDP** | |  |  |  |  |  |  |  |  |  |  |  |
| 54 and below*GDP |  |  | 1 (Ref) |  |  |  |  |  |  |  |  |  |
| 55-64*GDP |  |  | 0.90 (0.78, 1.05) | 0.18 |  |  |  |  |  |  |  |  |
| 65 and above*GDP |  |  | 0.90 (0.79, 1.03) | 0.12 |  |  |  |  |  |  |  |  |
| **Education*GDP** |  |  |  |  |  |  |  |  |  |  |  |  |
| Primary education and below*GDP |  |  |  |  | 1 (Ref) |  |  |  |  |  |  |  |
| College education*GDP |  |  |  |  | 0.79 (0.67, 0.93) | 0.00 |  |  |  |  |  |  |
| Secondary education*GDP |  |  |  |  | 0.83 (0.75, 0.92) | <0.001 |  |  |  |  |  |  |
| **Body-mass index group*GDP** | |  |  |  |  |  |  |  |  |  |  |  |
| Underweight*GDP | |  |  |  |  |  | 0.96 (0.83, 1.11) | 0.58 |  |  |  |  |
| Healthy weight*GDP |  |  |  |  |  |  | 1 (Ref) | . |  |  |  |  |
| Overweight*GDP |  |  |  |  |  |  | 0.96 (0.87, 1.06) | 0.41 |  |  |  |  |
| Obese*GDP |  |  |  |  |  |  | 0.99 (0.85, 1.17) | 0.95 |  |  |  |  |
| **Tobacco smoking*GDP** | |  |  |  |  |  |  |  |  |  |  |  |
| Never smoked*GDP | |  |  |  |  |  |  |  | 1 (Ref) |  |  |  |
| Ever smoked*GDP |  |  |  |  |  |  |  |  | 1.05 (0.95, 1.15) | 0.34 |  |  |
| **Urbanicity*GDP** | |  |  |  |  |  |  |  |  |  |  |  |
| Rural townships*GDP |  |  |  |  |  |  |  |  |  |  | 1 (Ref) |  |
| Urban areas*GDP |  |  |  |  |  |  |  |  |  |  | 0.76 (0.68, 0.85) | 0.00 |
| **Sex** |  |  |  |  |  |  |  |  |  |  |  |  |
| Female | 1 (Ref) |  | 1 (Ref) |  | 1 (Ref) |  | 1 (Ref) |  | 1 (Ref) |  | 1 (Ref) |  |
| Male | 1.50 (0.51, 4.402) | 0.46 | 1.18 (1.11, 1.25) | 0.00 | 1.18 (1.11, 1.25) | <0.001 | 1.18 (1.11, 1.25) | <0.001 | 1.18 (1.11, 1.25) | 0.00 | 1.18 (1.11, 1.25) | 0.00 |
| **Age, years** |  |  |  |  |  |  |  |  |  |  |  |  |
| 54 and below | 1 (Ref) |  | 1 (Ref) |  | 1 (Ref) |  | 1 (Ref) |  | 1 (Ref) |  | 1 (Ref) |  |
| 55-64 | 1.22 (1.14, 1.30) | 0.00 | 3.72 (0.73, 18.88) | 0.11 | 1.23 (1.15, 1.31) | <0.001 | 1.22 (1.14, 1.30) | <0.001 | 1.22 (1.14, 1.30) | 0.00 | 1.22 (1.14, 1.30) | 0.00 |
| 65 and above | 1.31 (1.23, 1.39) | 0.00 | 4.14 (0.96, 17.80) | 0.06 | 1.32 (1.24, 1.40) | <0.001 | 1.31 (1.23, 1.39) | <0.001 | 1.31 (1.23, 1.39) | 0.00 | 1.31 (1.23, 1.39) | 0.00 |
| **Education** |  |  |  |  |  |  |  |  |  |  |  |  |
| Primary education and below | 1 (Ref) |  | 1 (Ref) |  | 1 (Ref) |  | 1 (Ref) |  | 1 (Ref) |  | 1 (Ref) |  |
| College education | 1.18 (1.09, 1.28) | 0.00 | 1.18 (1.09, 1.28) | 0.00 | 15.95 (2.60, 97.88) | 0.00 | 1.18 (1.09, 1.28) | <0.001 | 1.18 (1.09, 1.28) | 0.00 | 1.18 (1.09, 1.28) | 0.00 |
| Secondary education | 1.06 (1.01 1.11) | 0.02 | 1.06 (1.01, 1.11) | 0.02 | 7.8 (2.66, 22.83) | <0.001 | 1.06 (1.01, 1.11) | 0.02 | 1.06 (1.01, 1.11) | 0.02 | 1.06 (1.01, 1.11) | 0.02 |
| **Body-mass index group** | |  |  |  |  |  |  |  |  |  |  |  |
| Underweight | 1.14 (1.07, 1.21) | 0.00 | 1.14 (1.07, 1.21) | 0.00 | 1.14 (1.07, 1.21) | <0.001 | 1.77 (0.37, 8.51) | 0.48 | 1.14 (1.07, 1.21) | 0.00 | 1.14 (1.07, 1.21) | 0.00 |
| Healthy weight | 1 (Ref) |  | 1 (Ref) |  | 1 (Ref) |  | 1 (Ref) |  | 1 (Ref) |  | 1 (Ref) |  |
| Overweight | 0.93 (0.88, 0.97) | 0.00 | 0.93 (0.88, 0.97) | 0.00 | 0.93 (0.88, 0.97) | 0.00 | 1.47 (0.49, 4.40) | 0.50 | 0.93 (0.88, 0.97) | 0.00 | 0.93 (0.88, 0.97) | 0.00 |
| Obese | 0.91 (0.85, 0.99) | 0.02 | 0.91 (0.85, 0.99) | 0.02 | 0.91 (0.84, 0.99) | 0.02 | 0.97 (0.17, 5.46) | 0.97 | 0.91 (0.85, 0.99) | 0.02 | 0.91 (0.85, 0.99) | 0.02 |
| **Tobacco smoking** | |  |  |  |  |  |  |  |  |  |  |  |
| Never smoked | 1 (Ref) |  | 1 (Ref) |  | 1 (Ref) |  | 1 (Ref) |  | 1 (Ref) |  | 1 (Ref) |  |
| Ever smoked | 0.82 (0.78, 0.87) | 0.00 | 0.82 (0.78, 0.87) | 0.00 | 0.82 (0.78, 0.87) | <0.001 | 0.82 (0.78, 0.87) | <0.001 | 0.5 (0.18, 1.39) | 0.18 | 0.82 (0.78, 0.87) | 0.00 |
| **Urbanicity** |  |  |  |  |  |  |  |  |  |  |  |  |
| Rural townships | 1 (Ref) |  | 1 (Ref) |  | 1 (Ref) |  | 1 (Ref) |  | 1 (Ref) |  | 1 (Ref) |  |
| Urban areas | 1.19 (1.13, 1.25) | 0.00 | 1.19 (1.13, 1.25) | 0.00 | 1.19 (1.13, 1.25) | <0.001 | 1.19 (1.13,1.2) | <0.001 | 1.19 (1.13, 1.25) | 0.00 | 24.06 (7.07, 81.82) | 0.00 |
| **GDP** | 1.04 (0.62, 1.75) | 0.87 | 1.13 (0.67, 1.91) | 0.65 | 1.15 (0.68, 1.95) | 0.61 | 1.05 (0.63, 1.74) | 0.87 | 1 (0.60, 1.66) | 0.99 | 1.24 (0.74, 2.07) | 0.42 |
| **Variable** | **Diagnosed-Model 1** | | **Diagnosed -Model 2** | | **Diagnosed -Model 3** | | **Diagnosed -Model 4** | | **Diagnosed -Model 5** | | **Diagnosed -Model 6** | |
|  | **RR (95% CI)** | **p-value** | **RR (95% CI)** | **p-value** | **RR (95% CI)** | **p-value** | **RR (95% CI)** | **p-value** | **RR (95% CI)** | **p-value** | **RR (95% CI)** | **p-value** |
| **Sex*GDP** | |  |  |  |  |  |  |  |  |  |  |  |
| Female*GDP | 1 (Ref) |  |  |  |  |  |  |  |  |  |  |  |
| Male*GDP | 1 (0.86, 1.16) | 0.98 |  |  |  |  |  |  |  |  |  |  |
| **Age, years*GDP** | |  |  |  |  |  |  |  |  |  |  |  |
| 54 and below*GDP |  |  | 1 (Ref) |  |  |  |  |  |  |  |  |  |
| 55-64*GDP |  |  | 0.94 (0.72, 1.24) | 0.68 |  |  |  |  |  |  |  |  |
| 65 and above*GDP |  |  | 0.89 (0.69, 1.15) | 0.37 |  |  |  |  |  |  |  |  |
| **Education*GDP** |  |  |  |  |  |  |  |  |  |  |  |  |
| Primary education and below*GDP |  |  |  |  | 1 (Ref) |  |  |  |  |  |  |  |
| College education*GDP |  |  |  |  | 0.75 (0.58, 0.96) | 0.02 |  |  |  |  |  |  |
| Secondary education*GDP |  |  |  |  | 0.95 (0.82, 1.10) | 0.52 |  |  |  |  |  |  |
| **Body-mass index group*GDP** | |  |  |  |  |  |  |  |  |  |  |  |
| Underweight*GDP | |  |  |  |  |  | 0.78 (0.63, 0.95) | 0.02 |  |  |  |  |
| Healthy weight*GDP |  |  |  |  |  |  | 1 (Ref) | . |  |  |  |  |
| Overweight*GDP |  |  |  |  |  |  | 1.01 (0.87, 1.18) | 0.88 |  |  |  |  |
| Obese*GDP |  |  |  |  |  |  | 1.19 (0.93, 1.53) | 0.16 |  |  |  |  |
| **Tobacco smoking*GDP** | |  |  |  |  |  |  |  |  |  |  |  |
| Never smoked*GDP | |  |  |  |  |  |  |  | 1 (Ref) |  |  |  |
| Ever smoked*GDP |  |  |  |  |  |  |  |  | 1.07 (0.93, 1.23) | 0.38 |  |  |
| **Urbanicity*GDP** | |  |  |  |  |  |  |  |  |  |  |  |
| Rural townships*GDP |  |  |  |  |  |  |  |  |  |  | 1 (Ref) |  |
| Urban areas*GDP |  |  |  |  |  |  |  |  |  |  | 0.66 (0.56, 0.77) | 0.00 |
| **Sex** |  |  |  |  |  |  |  |  |  |  |  |  |
| Female | 1 (Ref) |  | 1 (Ref) |  | 1 (Ref) |  | 1 (Ref) |  | 1 (Ref) |  | 1 (Ref) |  |
| Male | 1.18 (0.23, 6.14) | 0.85 | 1.15 (1.05, 1.26) | 0.00 | 1.15 (1.05, 1.26) | 0.00 | 1.15 (1.05, 1.25) | 0.00 | 1.15 (1.05, 1.26) | 0.00 | 1.15 (1.05, 1.26) | <0.001 |
| **Age, years** |  |  |  |  |  |  |  |  |  |  |  |  |
| 54 and below | 1 (Ref) |  | 1 (Ref) |  | 1 (Ref) |  | 1 (Ref) |  | 1 (Ref) |  | 1 (Ref) |  |
| 55-64 | 2.11 (1.86, 2.40) | <0.001 | 3.93 (0.20, 75.84) | 0.37 | 2.13 (1.88, 2.42) | <0.001 | 2.11 (1.86, 2.40) | <0.001 | 2.12 (1.86, 2.40) | <0.001 | 2.12 (1.87, 2.41) | 0.00 |
| 65 and above | 2.56 (2.28, 2.89) | <0.001 | 9.14 (0.58, 143.67) | 0.12 | 2.58 (2.29, 2.90) | <0.001 | 2.56 (2.27, 2.88) | <0.001 | 2.56 (2.28, 2.89) | <0.001 | 2.56 (2.28, 2.89) | <0.001 |
| **Education** |  |  |  |  |  |  |  |  |  |  |  |  |
| Primary education and below | 1 (Ref) |  | 1 (Ref) |  | 1 (Ref) |  | 1 (Ref) |  | 1 (Ref) |  | 1 (Ref) |  |
| College education | 1.26 (1.11, 1.42) | <0.001 | 1.26 (1.11, 1.42) | <0.001 | 30.78 (1.99, 476.00) | 0.01 | 1.26 (1.12, 1.42) | <0.001 | 1.26 (1.12, 1.43) | <0.001 | 1.26 (1.11, 1.42) | <0.001 |
| Secondary education | 1.15 (1.08, 1.24) | <0.001 | 1.16 (1.078, 1.24) | <0.001 | 1.94 (0.40, 9.51) | 0.41 | 1.16 (1.08, 1.24) | <0.001 | 1.16 (1.08, 1.24) | <0.001 | 1.16 (1.08, 1.249) | <0.001 |
| **Body-mass index group** | |  |  |  |  |  |  |  |  |  |  |  |
| Underweight | 1.39 (1.27, 1.52) | <0.001 | 1.39 (1.27, 1.52) | <0.001 | 1.39 (1.27, 1.52) | <0.001 | 20.99 (2.35, 187.32) | 0.01 | 1.39 (1.27, 1.52) | <0.001 | 1.39 (1.27, 1.52) | <0.001 |
| Healthy weight | 1 (Ref) |  | 1 (Ref) |  | 1 (Ref) |  | 1 (Ref) |  | 1 (Ref) |  | 1 (Ref) |  |
| Overweight | 0.95 (0.88, 1.02) | 0.12 | 0.95 (0.88, 1.02) | 0.13 | 0.95 (0.88, 1.02) | 0.13 | 0.83 (0.16, 4.20) | 0.82 | 0.94 (0.88, 1.02) | 0.12 | 0.95 (0.88, 1.02) | 0.13 |
| Obese | 0.85 (0.75, 0.96) | 0.01 | 0.85 (0.75, 0.96) | 0.01 | 0.85 (0.75, 0.96) | 0.01 | 0.12 (0.01, 1.85) | 0.13 | 0.85 (0.75, 0.96) | 0.01 | 0.85 (0.75, 0.96) | 0.01 |
| **Tobacco smoking** | |  |  |  |  |  |  |  |  |  |  |  |
| Never smoked | 1 (Ref) |  | 1 (Ref) |  | 1 (Ref) |  | 1 (Ref) |  | 1 (Ref) |  | 1 (Ref) |  |
| Ever smoked | 1.03 (0.95, 1.12) | 0.47 | 1.03 (0.95, 1.12) | 0.46 | 1.03 (0.95, 1.12) | 0.48 | 1.03 (0.95, 1.12) | 0.47 | 0.52 (0.11, 2.42) | 0.40 | 1.03 (0.95, 1.12) | 0.48 |
| **Urbanicity** |  |  |  |  |  |  |  |  |  |  |  |  |
| Rural townships | 1 (Ref) |  | 1 (Ref) |  | 1 (Ref) |  | 1 (Ref) |  | 1 (Ref) |  | 1 (Ref) |  |
| Urban areas | 1.06 (0.98, 1.15) | 0.13 | 1.06 (0.98, 1.15) | 0.14 | 1.05 (0.97, 1.14) | 0.19 | 1.06 (0.98, 1.15) | 0.13 | 1.06 (0.98, 1.15) | 0.14 | 96.75 (16.71, 560.21) | <0.001 |
| **GDP** | 1.16 (0.52, 2.59) | 0.73 | 1.27 (0.55, 2.92) | 0.57 | 1.21 (0.54, 2.72) | 0.64 | 1.16 (0.52, 2.59) | 0.72 | 1.1 (0.50, 2.45) | 0.81 | 1.51 (0.68, 3.38) | 0.32 |
| **Variable** | **Treated-Model 1** | | **Treated-Model 2** | | **Treated-Model 3** | | **Treated -Model 4** | | **Treated-Model 5** | | **Treated-Model 6** | |
|  | **RR (95% CI)** | **p-value** | **RR (95% CI)** | **p-value** | **RR (95% CI)** | **p-value** | **RR (95% CI)** | **p-value** | **RR (95% CI)** | **p-value** | **RR (95% CI)** | **p-value** |
| **Sex*GDP** | |  |  |  |  |  |  |  |  |  |  |  |
| Female*GDP | 1 (Ref) |  |  |  |  |  |  |  |  |  |  |  |
| Male*GDP | 0.93 (0.73, 1.18) | 0.55 |  |  |  |  |  |  |  |  |  |  |
| **Age, years*GDP** | |  |  |  |  |  |  |  |  |  |  |  |
| 54 and below*GDP |  |  | 1 (Ref) |  |  |  |  |  |  |  |  |  |
| 55-64*GDP |  |  | 0.86 (0.58, 1.27) | 0.44 |  |  |  |  |  |  |  |  |
| 65 and above*GDP |  |  | 0.83 (0.57, 1.19) | 0.31 |  |  |  |  |  |  |  |  |
| **Education*GDP** |  |  |  |  |  |  |  |  |  |  |  |  |
| Primary education and below*GDP |  |  |  |  | 1 (Ref) |  |  |  |  |  |  |  |
| College education*GDP |  |  |  |  | 0.95 (0.66, 1.38) | 0.79 |  |  |  |  |  |  |
| Secondary education*GDP |  |  |  |  | 1.00 (0.81, 1.24) | 0.99 |  |  |  |  |  |  |
| **Body-mass index group*GDP** | |  |  |  |  |  |  |  |  |  |  |  |
| Underweight*GDP | |  |  |  |  |  | 0.90 (0.67, 1.20) | 0.47 |  |  |  |  |
| Healthy weight*GDP |  |  |  |  |  |  | 1 (Ref) |  |  |  |  |  |
| Overweight*GDP |  |  |  |  |  |  | 0.92 (0.75, 1.14) | 0.47 |  |  |  |  |
| Obese*GDP |  |  |  |  |  |  | 1.05 (0.73, 1.52) | 0.80 |  |  |  |  |
| **Tobacco smoking*GDP** | |  |  |  |  |  |  |  |  |  |  |  |
| Never smoked*GDP | |  |  |  |  |  |  |  | 1 (Ref) |  |  |  |
| Ever smoked*GDP |  |  |  |  |  |  |  |  | 0.91 (0.74, 1.13) | 0.40 |  |  |
| **Urbanicity*GDP** | |  |  |  |  |  |  |  |  |  |  |  |
| Rural townships*GDP |  |  |  |  |  |  |  |  |  |  | 1 (Ref) | . |
| Urban areas*GDP |  |  |  |  |  |  |  |  |  |  | 0.78 (0.62, 1.00) | 0.05 |
| **Sex** |  |  |  |  |  |  |  |  |  |  |  |  |
| Female | 1 (Ref) |  | 1 (Ref) |  | 1 (Ref) |  | 1 (Ref) |  | 1 (Ref) |  | 1 (Ref) |  |
| Male | 2.94 (0.22, 39.24) | 0.41 | 1.32 (1.16, 1.52) | <0.001 | 1.33(1.16,1.52) | <0.001 | 1.33 (1.16, 1.52) | <0.001 | 1.32 (1.16, 1.52) | <0.001 | 1.32 (1.16, 1.52) | <0.001 |
| **Age, years** |  |  |  |  |  |  |  |  |  |  |  |  |
| 54 and below | 1 (Ref) |  | 1 (Ref) |  | 1 (Ref) |  | 1 (Ref) |  | 1 (Ref) |  | 1 (Ref) |  |
| 55-64 | 2.08 (1.73, 2.51) | <0.001 | 11.14 (0.15,836.23) | 0.27 | 2.08(1.73,2.51) | <0.001 | 2.08 (1.73, 2.51) | <0.001 | 2.08 (1.73, 2.51) | <0.001 | 2.08 (1.73, 2.51) | <0.001 |
| 65 and above | 2.47 (2.07, 2.94) | <0.001 | 19.80 (0.35, 1106.61) | 0.15 | 2.47(2.07, 2.94) | <0.001 | 2.47 (2.07, 2.94) | <0.001 | 2.47 (2.07, 2.94)) | <0.001 | 2.47 (2.07, 2.94) | <0.001 |
| **Education** |  |  |  |  |  |  |  |  |  |  |  |  |
| Primary education and below | 1 (Ref) |  | 1 (Ref) |  | 1 (Ref) |  | 1 (Ref) |  | 1 (Ref) |  | 1 (Ref) |  |
| College education | 1.45 (1.22, 1.72) | <0.001 | 1.45 (1.22, 1.72) | <0.001 | 2.56 (0.04,158.88) | 0.66 | 1.45 (1.22, 1.72) | <0.001 | 1.45 (1.22, 1.72) | <0.001 | 1.45 (1.22, 1.72) | <0.001 |
| Secondary education | 1.39 (1.25, 1.53) | <0.001 | 1.39 (1.26, 1.54) | <0.001 | 1.37 (0.13,13.96) | 0.79 | 1.39 (1.25, 1.54) | <0.001 | 1.39 (1.25, 1.53) | <0.001 | 1.39 (1.25, 1.54) | <0.001 |
| **Body-mass index group** | |  |  |  |  |  |  |  |  |  |  |  |
| Underweight | 1.18 (1.03, 1.35) | 0.02 | 1.18 (1.02, 1.35) | 0.02 | 1.18(1.03,1.35) | 0.02 | 3.73 (0.16, 87.78) | 0.41 | 1.18 (1.03, 1.35) | 0.02 | 1.18 (1.03, 1.35) | 0.02 |
| Healthy weight | 1 (Ref) |  | 1 (Ref) |  | 1 (Ref) |  | 1 (Ref) |  | 1 (Ref) |  | 1 (Ref) |  |
| Overweight | 0.91 (0.82, 1.01) | 0.08 | 0.91 (0.82, 1.01) | 0.08 | 0.91(0.82,1.01) | 0.08 | 2.18 (0.21, 22.75) | 0.51 | 0.91 (0.82, 1.01) | 0.08 | 0.91 (0.82, 1.01) | 0.08 |
| Obese | 0.81 (0.68, 0.96) | 0.02 | 0.81 (0.68, 0.96) | 0.02 | 0.81(0.68,0.96) | 0.02 | 0.47 (0.01, 27.41) | 0.72 | 0.81 (0.68, 0.96) | 0.02 | 0.81 (0.68, 0.96) | 0.02 |
| **Tobacco smoking** | |  |  |  |  |  |  |  |  |  |  |  |
| Never smoked | 1 (Ref) |  | 1 (Ref) |  | 1 (Ref) |  | 1 (Ref) |  | 1 (Ref) |  | 1 (Ref) |  |
| Ever smoked | 1.04 (0.92, 1.18) | 0.51 | 1.05 (0.93, 1.18) | 0.47 | 1.05(0.93,1.18) | 0.45 | 1.05 (0.93, 1.18) | 0.47 | 2.82 (0.28, 28.7) | 0.38 | 1.05 (0.93, 1.18) | 0.47 |
| **Urbanicity** |  |  |  |  |  |  |  |  |  |  |  |  |
| Rural townships | 1 (Ref) |  | 1 (Ref) |  | 1 (Ref) |  | 1 (Ref) |  | 1 (Ref) |  | 1 (Ref) |  |
| Urban areas | 1.24 (1.11, 1.39) | <0.001 | 1.24 (1.11, 1.39) | <0.001 | 1.24(1.11,1.39) | <0.001 | 1.24 (1.11, 1.39) | <0.001 | 1.24 (1.11, 1.39) | <0.001 | 17.11 (1.28, 228.7) | 0.03 |
| **GDP** | 1.74 (0.76, 3.97) | 0.19 | 1.94 (0.82, 4.64) | 0.13 | 1.65(0.73,3.71) | 0.23 | 1.69 (0.76, 3.78) | 0.20 | 1.75 (0.77, 3.97) | 0.18 | 1.93 (0.85, 4.35) | 0.12 |
| **Variable** | **Controlled 1-Model 1** | | **Controlled 1-Model 2** | | **Controlled 1-Model 3** | | **Controlled 1-Model 4** | | **Controlled 1-Model 5** | | **Controlled 1-Model 6** | |
|  | **RR (95% CI)** | **p-value** | **RR (95% CI)** | **p-value** | **RR (95% CI)** | **p-value** | **RR (95% CI)** | **p-value** | **RR (95% CI)** | **p-value** | **RR (95% CI)** | **p-value** |
| **Sex*GDP** | |  |  |  |  |  |  |  |  |  |  |  |
| Female*GDP | 1 (Ref) |  |  |  |  |  |  |  |  |  |  |  |
| Male*GDP | 0.85 (0.58, 1.23) | 0.38 |  |  |  |  |  |  |  |  |  |  |
| **Age, years*GDP** | |  |  |  |  |  |  |  |  |  |  |  |
| 54 and below*GDP |  |  | 1 (Ref) |  |  |  |  |  |  |  |  |  |
| 55-64*GDP |  |  | 0.90 (0.51, 1.58) | 0.72 |  |  |  |  |  |  |  |  |
| 65 and above*GDP |  |  | 1.03 (0.61, 1.76) | 0.91 |  |  |  |  |  |  |  |  |
| **Education*GDP** |  |  |  |  |  |  |  |  |  |  |  |  |
| Primary education and below*GDP |  |  |  |  | 1 (Ref) |  |  |  |  |  |  |  |
| College education*GDP |  |  |  |  | 0.80 (0.45, 1.41) | 0.44 |  |  |  |  |  |  |
| Secondary education*GDP |  |  |  |  | 1.09 (0.78, 1.52) | 0.60 |  |  |  |  |  |  |
| **Body-mass index group*GDP** | |  |  |  |  |  |  |  |  |  |  |  |
| Underweight*GDP | |  |  |  |  |  | 0.87 (0.55, 1.39) | 0.57 |  |  |  |  |
| Healthy weight*GDP |  |  |  |  |  |  | 1 (Ref) |  |  |  |  |  |
| Overweight*GDP |  |  |  |  |  |  | 0.80 (0.58, 1.09) | 0.16 |  |  |  |  |
| Obese*GDP |  |  |  |  |  |  | 1.15 (0.64, 2.09) | 0.64 |  |  |  |  |
| **Tobacco smoking*GDP** | |  |  |  |  |  |  |  |  |  |  |  |
| Never smoked*GDP | |  |  |  |  |  |  |  | 1 (Ref) |  |  |  |
| Ever smoked*GDP |  |  |  |  |  |  |  |  | 0.89 (0.64, 1.24) | 0.51 |  |  |
| **Urbanicity*GDP** | |  |  |  |  |  |  |  |  |  |  |  |
| Rural townships*GDP |  |  |  |  |  |  |  |  |  |  | 1 (Ref) |  |
| Urban areas*GDP |  |  |  |  |  |  |  |  |  |  | 0.86 (0.59, 1.25) | 0.44 |
| **Sex** |  |  |  |  |  |  |  |  |  |  |  |  |
| Female | 1 (Ref) |  | 1 (Ref) |  | 1 (Ref) |  | 1 (Ref) |  | 1 (Ref) |  | 1 (Ref) |  |
| Male | 8.44 (0.13, 537.99) | 0.32 | 1.31 (1.10, 1.57) | 0.00 | 1.31 (1.10, 1.57) | 0.00 | 1.31 (1.10, 1.57) | 0.00 | 1.31 (1.09, 1.57) | 0.00 | 1.31 (1.10, 1.57) | 0.00 |
| **Age, years** |  |  |  |  |  |  |  |  |  |  |  |  |
| 54 and below | 1 (Ref) |  | 1 (Ref) |  | 1 (Ref) |  | 1 (Ref) |  | 1 (Ref) |  | 1 (Ref) |  |
| 55-64 | 2.07 (1.62, 2.65) | <0.001 | 6.52 (0.01, 3264.64) | 0.56 | 2.08 (1.63, 2.66) | <0.001 | 2.07 (1.62, 2.65) | <0.001 | 2.07 (1.62, 2.64) | <0.001 | 2.07 (1.62, 2.65) | <0.001 |
| 65 and above | 2.11 (1.67, 2.66) | <0.001 | 1.50 (0.00, 537.49) | 0.89 | 2.11 (1.67, 2.67) | <0.001 | 2.11 (1.67, 2.66) | <0.001 | 2.11 (1.67, 2.66) | <0.001 | 2.10 (1.67, 2.66) | <0.001 |
| **Education** |  |  |  |  |  |  |  |  |  |  |  |  |
| Primary education and below | 1 (Ref) |  | 1 (Ref) |  | 1 (Ref) |  | 1 (Ref) |  | 1 (Ref) |  | 1 (Ref) |  |
| College education | 1.39 (1.10, 1.75) | 0.01 | 1.39 (1.11, 1.76) | 0.01 | 17.91 (0.03, 10768.69) | 0.38 | 1.39 (1.11, 1.76) | 0.01 | 1.39 (1.1, 1.75) | 0.01 | 1.39 (1.11, 1.76) | 0.01 |
| Secondary education | 1.57 (1.35, 1.81) | <0.001 | 1.57 (1.35, 1.81) | <0.001 | 0.59 (0.02, 22.82) | 0.78 | 1.57 (1.36, 1.82) | <0.001 | 1.57 (1.35, 1.82) | <0.001 | 1.57 (1.36, 1.82) | <0.001 |
| **Body-mass index group** | |  |  |  |  |  |  |  |  |  |  |  |
| Underweight | 1.02 (0.82, 1.27) | 0.86 | 1.02 (0.82, 1.27) | 0.85 | 1.02 (0.82, 1.26) | 0.87 | 4.51 (0.03, 772.18) | 0.57 | 1.02 (0.82, 1.27) | 0.86 | 1.02 (0.82, 1.27) | 0.86 |
| Healthy weight | 1 (Ref) |  | 1 (Ref) |  | 1 (Ref) |  | 1 (Ref) |  | 1 (Ref) |  | 1 (Ref) |  |
| Overweight | 0.92 (0.81, 1.06) | 0.26 | 0.92 (0.81, 1.06) | 0.25 | 0.92 (0.81, 1.06) | 0.25 | 11.72 (0.35, 394.78) | 0.17 | 0.92 (0.81, 1.06) | 0.26 | 0.92 (0.81, 1.06) | 0.26 |
| Obese | 0.76 (0.6, 0.96) | 0.02 | 0.76 (0.60, 0.96) | 0.02 | 0.76 (0.6, 0.96) | 0.02 | 0.15 (0, 119.66) | 0.58 | 0.76 (0.6, 0.96) | 0.02 | 0.76 (0.60, 0.96) | 0.02 |
| **Tobacco smoking** | |  |  |  |  |  |  |  |  |  |  |  |
| Never smoked | 1 (Ref) |  | 1 (Ref) |  | 1 (Ref) |  | 1 (Ref) |  | 1 (Ref) |  | 1 (Ref) |  |
| Ever smoked | 1.04 (0.88, 1.22) | 0.68 | 1.04 (0.88, 1.23) | 0.63 | 1.04 (0.88, 1.23) | 0.64 | 1.04 (0.88, 1.23) | 0.63 | 3.59 (0.09, 137.73) | 0.49 | 1.04 (0.88, 1.23) | 0.63 |
| **Urbanicity** |  |  |  |  |  |  |  |  |  |  |  |  |
| Rural townships | 1 (Ref) |  | 1 (Ref) |  | 1 (Ref) |  | 1 (Ref) |  | 1 (Ref) |  | 1 (Ref) |  |
| Urban areas | 1.43 (1.21, 1.69) | <0.001 | 1.43 (1.21, 1.69) | <0.001 | 1.42 (1.2, 1.68) | <0.001 | 1.42 (1.2, 1.69) | <0.001 | 1.43 (1.21, 1.69) | <0.001 | 7.32 (0.12, 447.63) | 0.34 |
| **GDP** | 3.85 (1.54, 9.59) | 0.00 | 3.38 (1.27, 9.05) | 0.02 | 3.26 (1.35, 7.85) | 0.01 | 3.61 (1.52, 8.58) | 0.00 | 3.63 (1.49, 8.88) | 0.01 | 3.68 (1.52, 8.95) | 0.00 |
| **Variable** | **Controlled 2-Model 1** | | **Controlled 2-Model 2** | | **Controlled 2-Model 3** | | **Controlled 2-Model 4** | | **Controlled 2-Model 5** | | **Controlled 2-Model 6** | |
|  | **RR (95% CI)** | **p-value** | **RR (95% CI)** | **p-value** | **RR (95% CI)** | **p-value** | **RR (95% CI)** | **p-value** | **RR (95% CI)** | **p-value** | **RR (95% CI)** | **p-value** |
| **Sex*GDP** | |  |  |  |  |  |  |  |  |  |  |  |
| Female*GDP | 1 (Ref) |  |  |  |  |  |  |  |  |  |  |  |
| Male*GDP | 0.80 (0.51, 1.24) | 0.32 |  |  |  |  |  |  |  |  |  |  |
| **Age, years*GDP** | |  |  |  |  |  |  |  |  |  |  |  |
| 54 and below*GDP |  |  | 1 (Ref) |  |  |  |  |  |  |  |  |  |
| 55-64*GDP |  |  | 0.74 (0.39, 1.41) | 0.37 |  |  |  |  |  |  |  |  |
| 65 and above*GDP |  |  | 0.91 (0.50, 1.67) | 0.76 |  |  |  |  |  |  |  |  |
| **Education*GDP** |  |  |  |  |  |  |  |  |  |  |  |  |
| Primary education and below*GDP |  |  |  |  | 1 (Ref) |  |  |  |  |  |  |  |
| College education*GDP |  |  |  |  | 0.78 (0.41, 1.51) | 0.47 |  |  |  |  |  |  |
| Secondary education*GDP |  |  |  |  | 1.30 (0.89, 1.90) | 0.18 |  |  |  |  |  |  |
| **Body-mass index group*GDP** | |  |  |  |  |  |  |  |  |  |  |  |
| Underweight*GDP | |  |  |  |  |  | 0.80 (0.47, 1.35) | 0.40 |  |  |  |  |
| Healthy weight*GDP |  |  |  |  |  |  | 1 (Ref) |  |  |  |  |  |
| Overweight*GDP |  |  |  |  |  |  | 0.67 (0.47, 0.96) | 0.03 |  |  |  |  |
| Obese*GDP |  |  |  |  |  |  | 1.03 (0.52, 2.03) | 0.93 |  |  |  |  |
| **Tobacco smoking*GDP** | |  |  |  |  |  |  |  |  |  |  |  |
| Never smoked*GDP | |  |  |  |  |  |  |  | 1 (Ref) |  |  |  |
| Ever smoked*GDP |  |  |  |  |  |  |  |  | 0.89 (0.61, 1.31) | 0.56 |  |  |
| **Urbanicity*GDP** | |  |  |  |  |  |  |  |  |  |  |  |
| Rural townships*GDP |  |  |  |  |  |  |  |  |  |  | 1 (Ref) |  |
| Urban areas*GDP |  |  |  |  |  |  |  |  |  |  | 0.75 (0.49, 1.14) | 0.17 |
| **Sex** |  |  |  |  |  |  |  |  |  |  |  |  |
| Female | 1 (Ref) |  | 1 (Ref) |  | 1 (Ref) |  | 1 (Ref) |  | 1 (Ref) |  | 1 (Ref) |  |
| Male | 16.4 (0.12, 2288.07) | 0.27 | 1.32 (1.08, 1.60) | 0.01 | 1.32 (1.08, 1.60) | 0.01 | 1.32 (1.08, 1.60) | 0.00 | 1.32 (1.08, 1.60) | 0.01 | 1.32 (1.08, 1.60) | 0.01 |
| **Age, years** |  |  |  |  |  |  |  |  |  |  |  |  |
| 54 and below | 1 (Ref) |  | 1 (Ref) |  | 1 (Ref) |  | 1 (Ref) |  | 1 (Ref) |  | 1 (Ref) |  |
| 55-64 | 1.90 (1.46, 2.47) | <0.001 | 50.89 (0.04, 64046.77) | 0.28 | 1.91 (1.46, 2.48) | <0.001 | 1.90 (1.46, 2.47) | <0.001 | 1.90 (1.46, 2.47) | <0.001 | 1.90 (1.46, 2.47) | <0.001 |
| 65 and above | 1.97 (1.54, 2.53) | <0.001 | 5.57 (0.01, 4745.15) | 0.62 | 1.98 (1.54, 2.54) | <0.001 | 1.98 (1.54, 2.53) | <0.001 | 1.97 (1.54, 2.53) | <0.001 | 1.97 (1.54, 2.52) | <0.001 |
| **Education** |  |  |  |  |  |  |  |  |  |  |  |  |
| Primary education and below | 1 (Ref) |  | 1 (Ref) |  | 1 (Ref) |  | 1 (Ref) |  | 1 (Ref) |  | 1 (Ref) |  |
| College education | 1.33 (1.03, 1.71) | 0.03 | 1.33 (1.04, 1.71) | 0.03 | 21.95 (0.01, 35084.47) | 0.41 | 1.33 (1.03, 1.71) | 0.03 | 1.33 (1.03, 1.71) | 0.03 | 1.33 (1.04, 1.71) | 0.03 |
| Secondary education | 1.60 (1.36, 1.88) | <0.001 | 1.60 (1.36, 1.88) | <0.001 | 0.09 (0, 6.13) | 0.26 | 1.60 (1.37, 1.88) | <0.001 | 1.60 (1.36, 1.88) | <0.001 | 1.60 (1.37, 1.88) | <0.001 |
| **Body-mass index group** | |  |  |  |  |  |  |  |  |  |  |  |
| Underweight | 1.01 (0.79, 1.28) | 0.97 | 1.01 (0.79, 1.28) | 0.95 | 1.00 (0.79, 1.27) | 0.98 | 12.13 (0.04, 4055.34) | 0.40 | 1.01 (0.79, 1.28) | 0.96 | 1.01 (0.79, 1.28) | 0.97 |
| Healthy weight | 1 (Ref) |  | 1 (Ref) |  | 1 (Ref) |  | 1 (Ref) |  | 1 (Ref) |  | 1 (Ref) |  |
| Overweight | 0.94 (0.81, 1.09) | 0.42 | 0.94 (0.81, 1.09) | 0.41 | 0.94 (0.81, 1.09) | 0.41 | 80.82 (1.49, 4386.43) | 0.03 | 0.94 (0.81, 1.09) | 0.41 | 0.94 (0.81, 1.09) | 0.42 |
| Obese | 0.78 (0.61, 1.01) | 0.06 | 0.78 (0.61, 1.01) | 0.06 | 0.78 (0.61, 1.01) | 0.06 | 0.55 (0.00, 1142.24) | 0.89 | 0.78 (0.61, 1.01) | 0.06 | 0.78 (0.61, 1.01) | 0.05 |
| **Tobacco smoking** | |  |  |  |  |  |  |  |  |  |  |  |
| Never smoked | 1 (Ref) |  | 1 (Ref) |  | 1 (Ref) |  | 1 (Ref) |  | 1 (Ref) |  | 1 (Ref) |  |
| Ever smoked | 1.07 (0.89, 1.28) | 0.49 | 1.07 (0.90, 1.28) | 0.45 | 1.07 (0.89, 1.28) | 0.46 | 1.07 (0.90, 1.29) | 0.44 | 3.82 (0.05, 272.53) | 0.54 | 1.07 (0.9, 1.28) | 0.44 |
| **Urbanicity** |  |  |  |  |  |  |  |  |  |  |  |  |
| Rural townships | 1 (Ref) |  | 1 (Ref) |  | 1 (Ref) |  | 1 (Ref) |  | 1 (Ref) |  | 1 (Ref) |  |
| Urban areas | 1.49 (1.24, 1.80) | <0.001 | 1.50 (1.24, 1.81) | <0.001 | 1.48 (1.23, 1.79) | <0.001 | 1.49 (1.23, 1.80) | <0.001 | 1.49 (1.24, 1.80) | <0.001 | 37.06 (0.37, 3735.53) | 0.13 |
| **GDP** | 5.27 (1.78, 15.59) | 0.00 | 5.04 (1.59, 15.99) | 0.01 | 3.87 (1.38, 10.9) | 0.01 | 5.06 (1.82, 14.09) | 0.00 | 4.75 (1.65, 13.7) | 0.00 | 5.25 (1.83, 15.01) | 0.00 |

Table S7. Multicollinearity test by calculating variance inflation factor (VIF). Note: Controlled 1 = Controlled (mild or no exacerbations), Controlled 2 = Controlled (no exacerbations).

|  | | **VIF value** | | | | |
| --- | --- | --- | --- | --- | --- | --- |
| **Variable** | | **Tested** | **Diagnosed** | **Treated** | **Controlled 1** | **Controlled SM)** |
| **Sex** | |  |  |  |  |  |
| Female | | 1(ref) | 1(ref) | 1(ref) | 1(ref) | 1(ref) |
| Male | | 1.79 | 1.79 | 1.79 | 1.79 | 1.79 |
| **Age, years** | |  |  |  |  |  |
| 54 and below | | 1(ref) | 1(ref) | 1(ref) | 1(ref) | 1(ref) |
| 55-64 | | 1.88 | 1.88 | 1.88 | 1.88 | 1.88 |
| 65 and above | | 2.06 | 2.06 | 2.06 | 2.06 | 2.06 |
| **Education** | |  |  |  |  |  |
| Primary education and below | | 1(ref) | 1(ref) | 1(ref) | 1(ref) | 1(ref) |
| College education | | 1.24 | 1.24 | 1.24 | 1.24 | 1.24 |
| Secondary education | | 1.3 | 1.3 | 1.3 | 1.3 | 1.3 |
| **Body-mass index group** | |  |  |  |  |  |
| Underweight | | 1.08 | 1.08 | 1.08 | 1.08 | 1.08 |
| Healthy weight | | 1(ref) | 1(ref) | 1(ref) | 1(ref) | 1(ref) |
| Overweight | | 1.14 | 1.14 | 1.14 | 1.14 | 1.14 |
| Obese | | 1.08 | 1.08 | 1.08 | 1.08 | 1.08 |
| **Tobacco smoking** | |  |  |  |  |  |
| Never smoked | | 1(ref) | 1(ref) | 1(ref) | 1(ref) | 1(ref) |
| Ever smoked | | 1.87 | 1.87 | 1.87 | 1.87 | 1.87 |
| **Urbanicity** |  |  |  |  |  |  |
| Rural townships | | 1(ref) | 1(ref) | 1(ref) | 1(ref) | 1(ref) |
| Urban areas | | 1.58 | 1.58 | 1.58 | 1.58 | 1.58 |
| **Region** |  |  |  |  |  |  |
| Baiyin | | 4.39 | 4.39 | 4.39 | 4.39 | 4.39 |
| Beijing | | 1.5 | 1.5 | 1.5 | 1.5 | 1.5 |
| Cangzhou | | 1.91 | 1.91 | 1.91 | 1.91 | 1.91 |
| Chifeng | | 4.31 | 4.31 | 4.31 | 4.31 | 4.31 |
| Chongqing | | 2.12 | 2.12 | 2.12 | 2.12 | 2.12 |
| Daqing | | 2.72 | 2.72 | 2.72 | 2.72 | 2.72 |
| Datong | | 6.2 | 6.2 | 6.2 | 6.2 | 6.2 |
| Deyang | | 1.21 | 1.21 | 1.21 | 1.21 | 1.21 |
| Huaihua | | 8.5 | 8.5 | 8.5 | 8.5 | 8.5 |
| Huangshan | | 2.47 | 2.47 | 2.47 | 2.47 | 2.47 |
| Hulunbeier | | 3.02 | 3.02 | 3.02 | 3.02 | 3.02 |
| Huzhou | | 1.83 | 1.83 | 1.83 | 1.83 | 1.83 |
| Luoyang | | 1.23 | 1.23 | 1.23 | 1.23 | 1.23 |
| Tianjin | | 3.54 | 3.54 | 3.54 | 3.54 | 3.54 |
| Weifang | | 3.44 | 3.44 | 3.44 | 3.44 | 3.44 |
| Wuhan | | 1.56 | 1.56 | 1.56 | 1.56 | 1.56 |
| Xiangtan | | 4.79 | 4.79 | 4.79 | 4.79 | 4.79 |
| Yinchuan | | 6.17 | 6.17 | 6.17 | 6.17 | 6.17 |
| Zhoukou | | 3.1 | 3.1 | 3.1 | 3.1 | 3.1 |
| Zunyi | | 10.87 | 10.87 | 10.87 | 10.87 | 10.87 |

Table S8. VIFs when excluding observations from Zunyi. Note: Controlled 1 = Controlled (mild or no exacerbations), Controlled 2 = Controlled (no exacerbations).

|  | | **VIF value** | | | | |
| --- | --- | --- | --- | --- | --- | --- |
| **Variable** | | **Tested** | **Diagnosed** | **Treated** | **Controlled 1** | **Controlled SM)** |
| **Sex** | |  |  |  |  |  |
| Female | | 1(ref) | 1(ref) | 1(ref) | 1(ref) | 1(ref) |
| Male | | 1.76 | 1.76 | 1.76 | 1.76 | 1.76 |
| **Age, years** | |  |  |  |  |  |
| 54 and below | | 1(ref) | 1(ref) | 1(ref) | 1(ref) | 1(ref) |
| 55-64 | | 2.02 | 2.02 | 2.02 | 2.02 | 2.02 |
| 65 and above | | 2.20 | 2.20 | 2.20 | 2.20 | 2.20 |
| **Education** | |  |  |  |  |  |
| Primary education and below | | 1(ref) | 1(ref) | 1(ref) | 1(ref) | 1(ref) |
| College education | | 1.26 | 1.26 | 1.26 | 1.26 | 1.26 |
| Secondary education | | 1.31 | 1.31 | 1.31 | 1.31 | 1.31 |
| **Body-mass index group** | |  |  |  |  |  |
| Underweight | | 1.09 | 1.09 | 1.09 | 1.09 | 1.09 |
| Healthy weight | | 1(ref) | 1(ref) | 1(ref) | 1(ref) | 1(ref) |
| Overweight | | 1.15 | 1.15 | 1.15 | 1.15 | 1.15 |
| Obese | | 1.08 | 1.08 | 1.08 | 1.08 | 1.08 |
| **Tobacco smoking** | |  |  |  |  |  |
| Never smoked | | 1(ref) | 1(ref) | 1(ref) | 1(ref) | 1(ref) |
| Ever smoked | | 1.84 | 1.84 | 1.84 | 1.84 | 1.84 |
| **Urbanicity** |  |  |  |  |  |  |
| Rural townships | | 1(ref) | 1(ref) | 1(ref) | 1(ref) | 1(ref) |
| Urban areas | | 1.61 | 1.61 | 1.61 | 1.61 | 1.61 |
| **Region** |  |  |  |  |  |  |
| Baiyin | | 4.33 | 4.33 | 4.33 | 4.33 | 4.33 |
| Beijing | | 1.50 | 1.50 | 1.50 | 1.50 | 1.50 |
| Cangzhou | | 1.91 | 1.91 | 1.91 | 1.91 | 1.91 |
| Chifeng | | 4.26 | 4.26 | 4.26 | 4.26 | 4.26 |
| Chongqing | | 2.11 | 2.11 | 2.11 | 2.11 | 2.11 |
| Daqing | | 2.74 | 2.74 | 2.74 | 2.74 | 2.74 |
| Datong | | 6.06 | 6.06 | 6.06 | 6.06 | 6.06 |
| Deyang | | 1.21 | 1.21 | 1.21 | 1.21 | 1.21 |
| Huaihua | | 8.15 | 8.15 | 8.15 | 8.15 | 8.15 |
| Huangshan | | 2.46 | 2.46 | 2.46 | 2.46 | 2.46 |
| Hulunbeier | | 3.00 | 3.00 | 3.00 | 3.00 | 3.00 |
| Huzhou | | 1.83 | 1.83 | 1.83 | 1.83 | 1.83 |
| Luoyang | | 1.23 | 1.23 | 1.23 | 1.23 | 1.23 |
| Tianjin | | 3.53 | 3.53 | 3.53 | 3.53 | 3.53 |
| Weifang | | 3.41 | 3.41 | 3.41 | 3.41 | 3.41 |
| Wuhan | | 1.56 | 1.56 | 1.56 | 1.56 | 1.56 |
| Xiangtan | | 4.72 | 4.72 | 4.72 | 4.72 | 4.72 |
| Yinchuan | | 6.05 | 6.05 | 6.05 | 6.05 | 6.05 |
| Zhoukou | | 3.07 | 3.07 | 3.07 | 3.07 | 3.07 |

**Figure S2. Overall COPD care cascade outcomes in China prior to intervention under the ‘Happy Breathing’ Programme.** Note: Data are percentages with 95% CIs. Treated 2=Pharmaceutical and non-pharmaceutical treatment. Non-pharmaceutical treatment includes vaccination, smoking cessation, long-term oxygen therapy, and domiciliary non-invasive ventilation. Controlled 1 = Controlled (mild or no exacerbations), Controlled 2 = Controlled (no exacerbations).


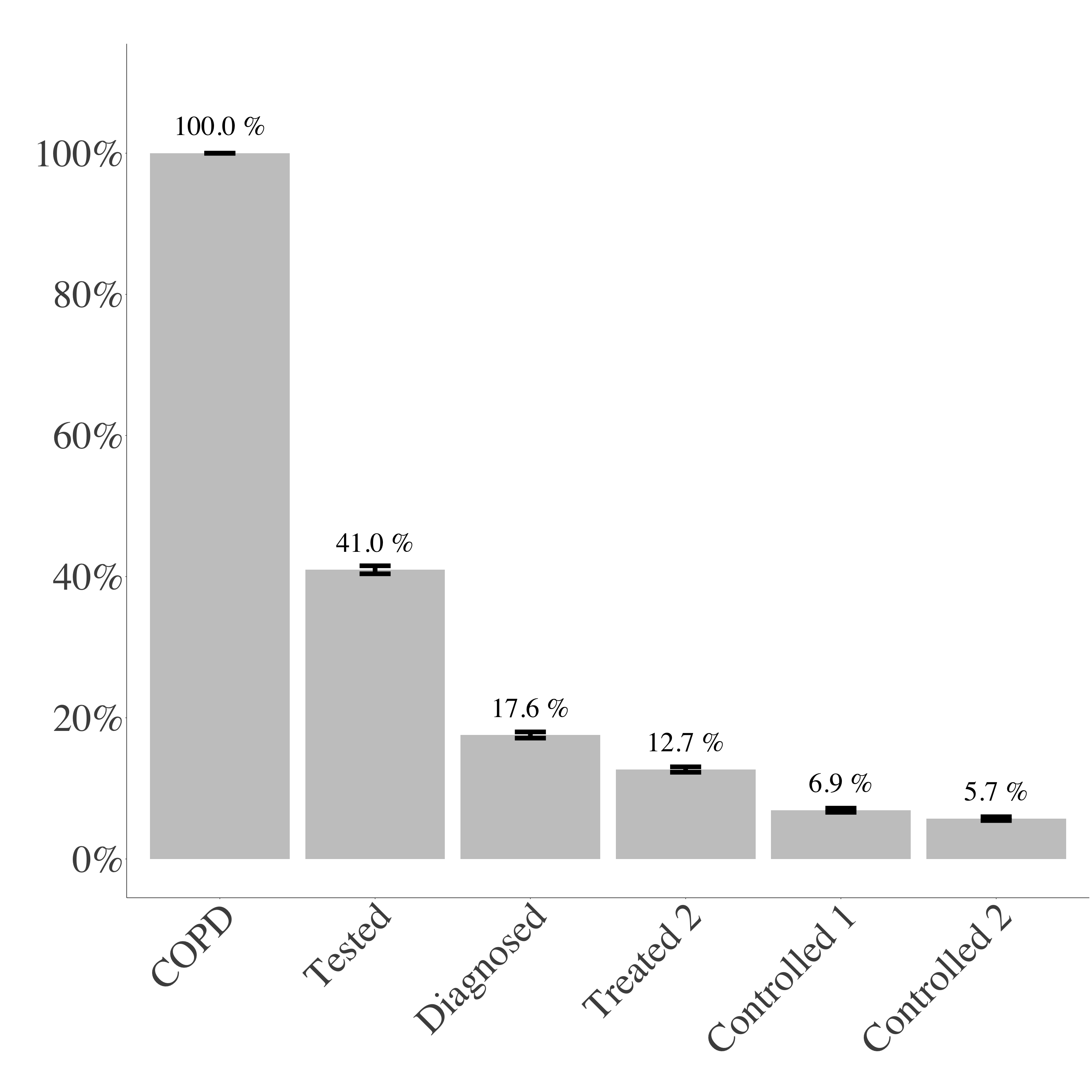


Table S9. Beta regressions of each cascade stage onto log-transformed per-capita GDP from 2014 to 2019. Note: COPD=chronic obstructive pulmonary disease, Controlled 1 = Controlled (mild or no exacerbations), Controlled 2 = Controlled (no exacerbations). “Per-capita GDP” ranges between 2014-2019. Regions with sample size under 100 were not included in this analysis.

|  | **Tested** | **Diagnosed** | **Treated** | **Controlled 1** | **Controlled 2** |
| --- | --- | --- | --- | --- | --- |
| Log per-capita GDP | 0.68 | 0.68 | 0.61 | 1.14 | 1.51 |
| Std. Error | 0.01 | 0.01 | 0.01 | 0.01 | 0.01 |
| p-value | <0.001 | <0.001 | <0.001 | <0.001 | <0.001 |

Figure S3. COPD care cascade outcomes per-capita by GDP Note: “Per-capita GDP” ranges between 2014-2019. Regions with sample size under 100 were not included in this analysis. The grey boundary shows the point-wise 95% prediction interval, and the vertical bars are 95% CIs around point estimates. Regional labels are not shown in the controlled plot to avoid visual overcrowding. AK=Ankang. BJ=Beijing. BY=Baiyin. CF=Chifeng. CQ=Chongqing. CZ=Cangzhou. DQ=Daqing. DT=Datong. DY=Deyang. GDP=Gross Domestic Product. HH=Huaihua. HLBE=Hulunbeier. HS=Huangshan. HZ=Huzhou. LY=Luoyang. TJ=Tianjin. WF=Weifang. WH=Wuhan. XT=Xiangtan. YC=Yinchuan. ZK=Zhoukou. ZY=Zunyi. Controlled 1 = Controlled (mild or no exacerbations), Controlled 2 = Controlled (no exacerbations).


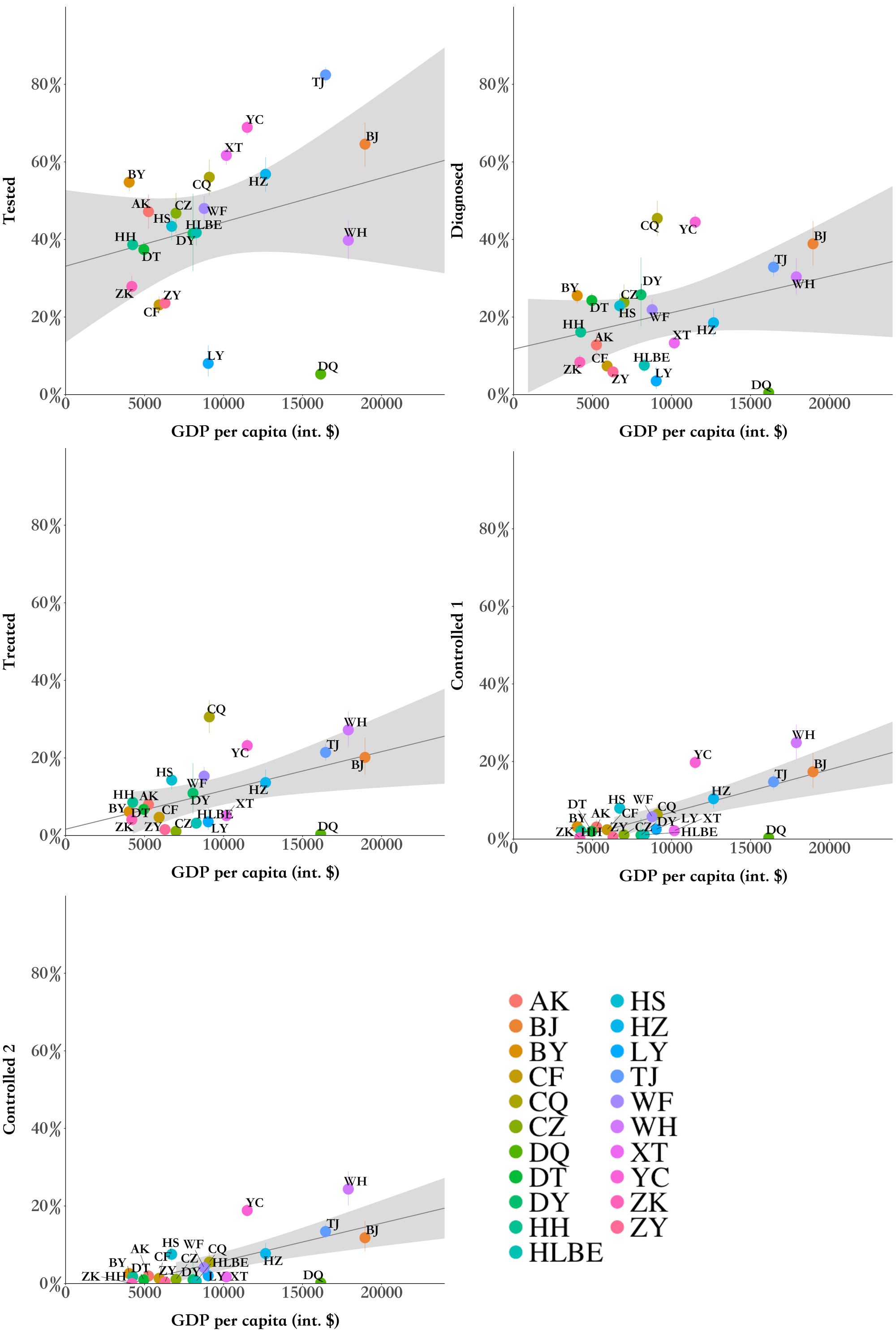


Figure S4.The percentage of people with COPD aged 54 years and younger who achieved each stage in the COPD care cascade by regional per-capita GDP in the Happy Breathing Programme in China. Note: “Per-capita GDP” ranges between 2014-2019. Regions with sample size under 100 were not included in this analysis. The grey boundary shows the point-wise 95% prediction interval, and the vertical bars are 95% CIs around point estimates. Region labels are not shown in the controlled plot to avoid visual overcrowding. AK=Ankang. BJ=Beijing. BY=Baiyin. CF=Chifeng. CQ=Chongqing. CZ=Cangzhou. DQ=Daqing. DT=Datong. DY=Deyang. GDP=Gross Domestic Product. HH=Huaihua. HLBE=Hulunbeier. HS=Huangshan. HZ=Huzhou. LY=Luoyang. TJ=Tianjin. WF=Weifang. WH=Wuhan. XT=Xiangtan. YC=Yinchuan. ZK=Zhoukou. ZY=Zunyi. Controlled 1 = Controlled (mild or no exacerbations), Controlled 2 = Controlled (no exacerbations).


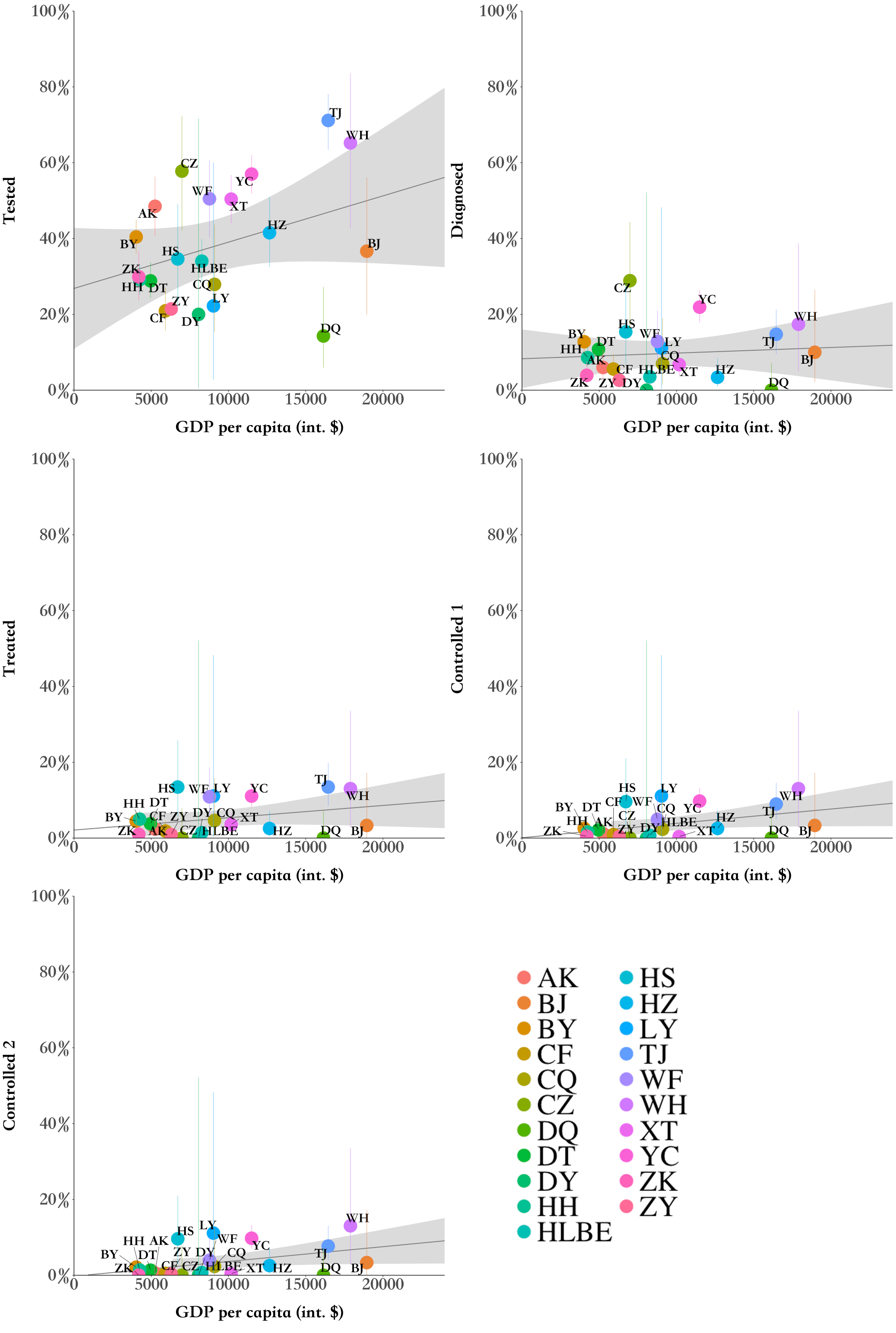


Figure S5. The percentage of people with COPD aged 55-64 years old who achieved each stage in the COPD care cascade by regional per-capita GDP in the Happy Breathing Programme in China. Note: “Per-capita GDP” ranges between 2014-2019. Regions with sample size under 100 were not included in this analysis. The grey boundary shows the point-wise 95% prediction interval, and the vertical bars are 95% CIs around point estimates. Region labels are not shown in the controlled plot to avoid visual overcrowding. AK=Ankang. BJ=Beijing. BY=Baiyin. CF=Chifeng. CQ=Chongqing. CZ=Cangzhou. DQ=Daqing. DT=Datong. DY=Deyang. GDP=Gross Domestic Product. HH=Huaihua. HLBE=Hulunbeier. HS=Huangshan. HZ=Huzhou. LY=Luoyang. TJ=Tianjin. WF=Weifang. WH=Wuhan. XT=Xiangtan. YC=Yinchuan. ZK=Zhoukou. ZY=Zunyi. Controlled 1 = Controlled (mild or no exacerbations), Controlled 2 = Controlled (no exacerbations).

**
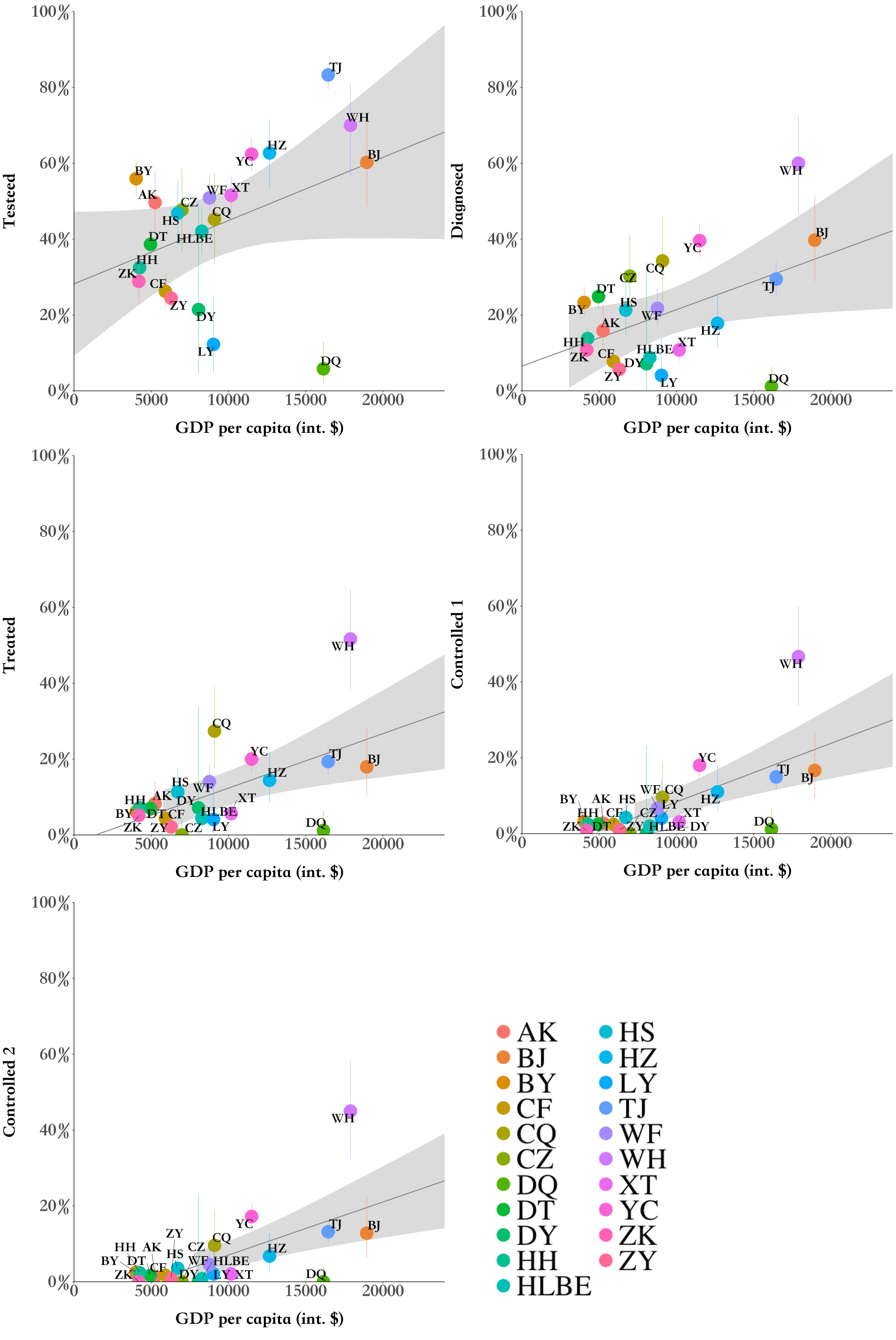
**

Figure S6. The percentage of people with COPD aged 65 years and older who achieved each stage in the COPD care cascade by regional per-capita GDP in the Happy Breathing Programme in China. Note: “GDP per capita” is the average GDP per capita in 2014-2019. Regions with sample size under 100 were not included in this analysis. The grey boundary shows the point-wise 95% prediction interval, and the vertical bars are 95% CIs around point estimates. Region labels are not shown in the controlled plot to avoid visual overcrowding. AK=Ankang. BJ=Beijing. BY=Baiyin. CF=Chifeng. CQ=Chongqing. CZ=Cangzhou. DQ=Daqing. DT=Datong. DY=Deyang. GDP=Gross Domestic Product. HH=Huaihua. HLBE=Hulunbeier. HS=Huangshan. HZ=Huzhou. LY=Luoyang. TJ=Tianjin. WF=Weifang. WH=Wuhan. XT=Xiangtan. YC=Yinchuan. ZK=Zhoukou. ZY=Zunyi. Controlled 1 = Controlled (mild or no exacerbations), Controlled 2 = Controlled (no exacerbations).


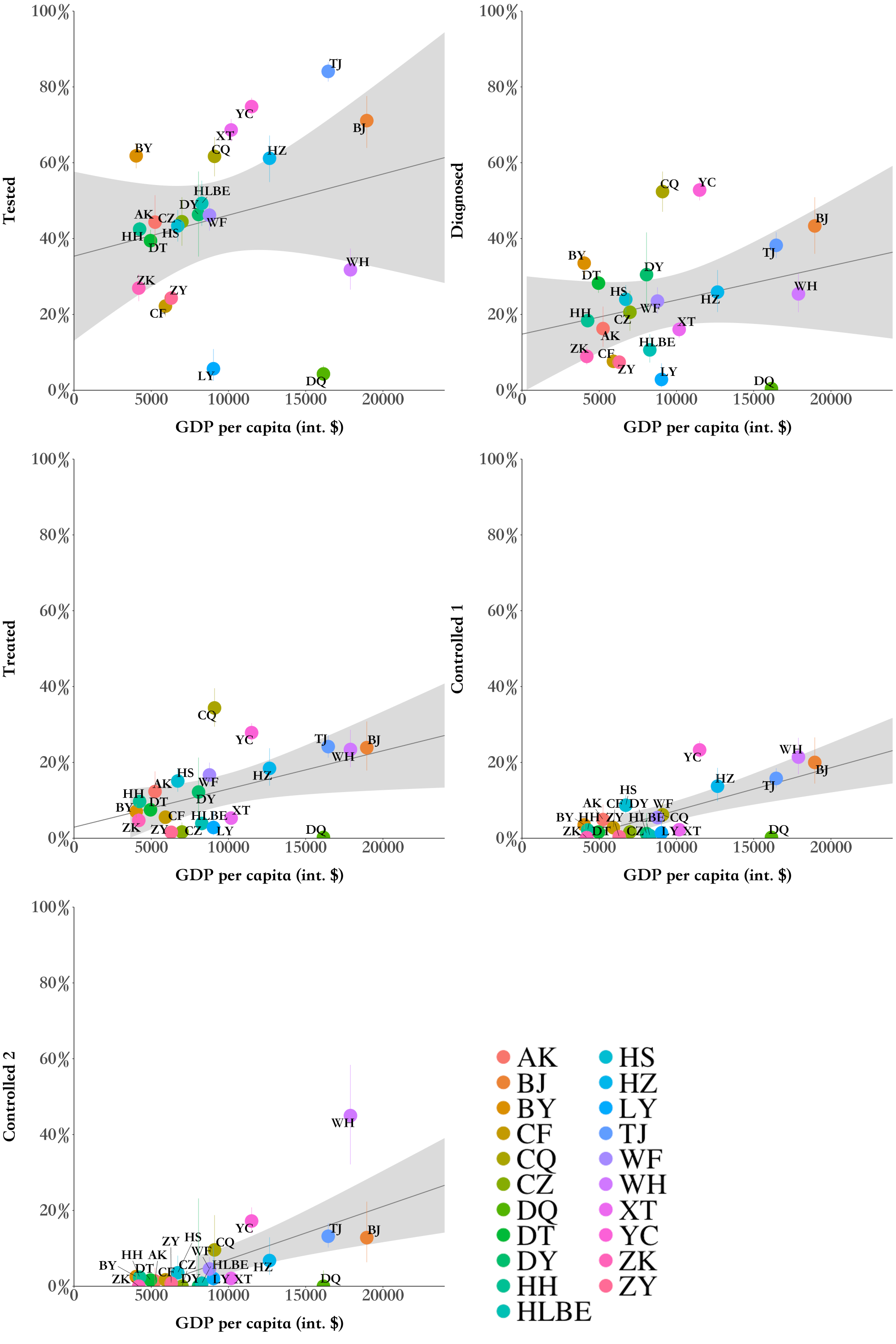


Figure S7. The percentage of people with COPD who achieved each stage in the COPD care cascade by regional PM2.5 in the Happy Breathing Programme in China. Note: “PM2.5” is the average annual PM2.5 in 2014-2019. Regions with sample size under 100 were not included in this analysis. The grey boundary shows the point-wise 95% prediction interval, and the vertical bars are 95% CIs around point estimates. Region labels are not shown in the controlled plot to avoid visual overcrowding. AK=Ankang. BJ=Beijing. BY=Baiyin. CF=Chifeng. CQ=Chongqing. CZ=Cangzhou. DQ=Daqing. DT=Datong. DY=Deyang. GDP=Gross Domestic Product. HH=Huaihua. HLBE=Hulunbeier. HS=Huangshan. HZ=Huzhou. LY=Luoyang. TJ=Tianjin. WF=Weifang. WH=Wuhan. XT=Xiangtan. YC=Yinchuan. ZK=Zhoukou. ZY=Zunyi. Controlled 1 = Controlled (mild or no exacerbations), Controlled 2 = Controlled (no exacerbations).


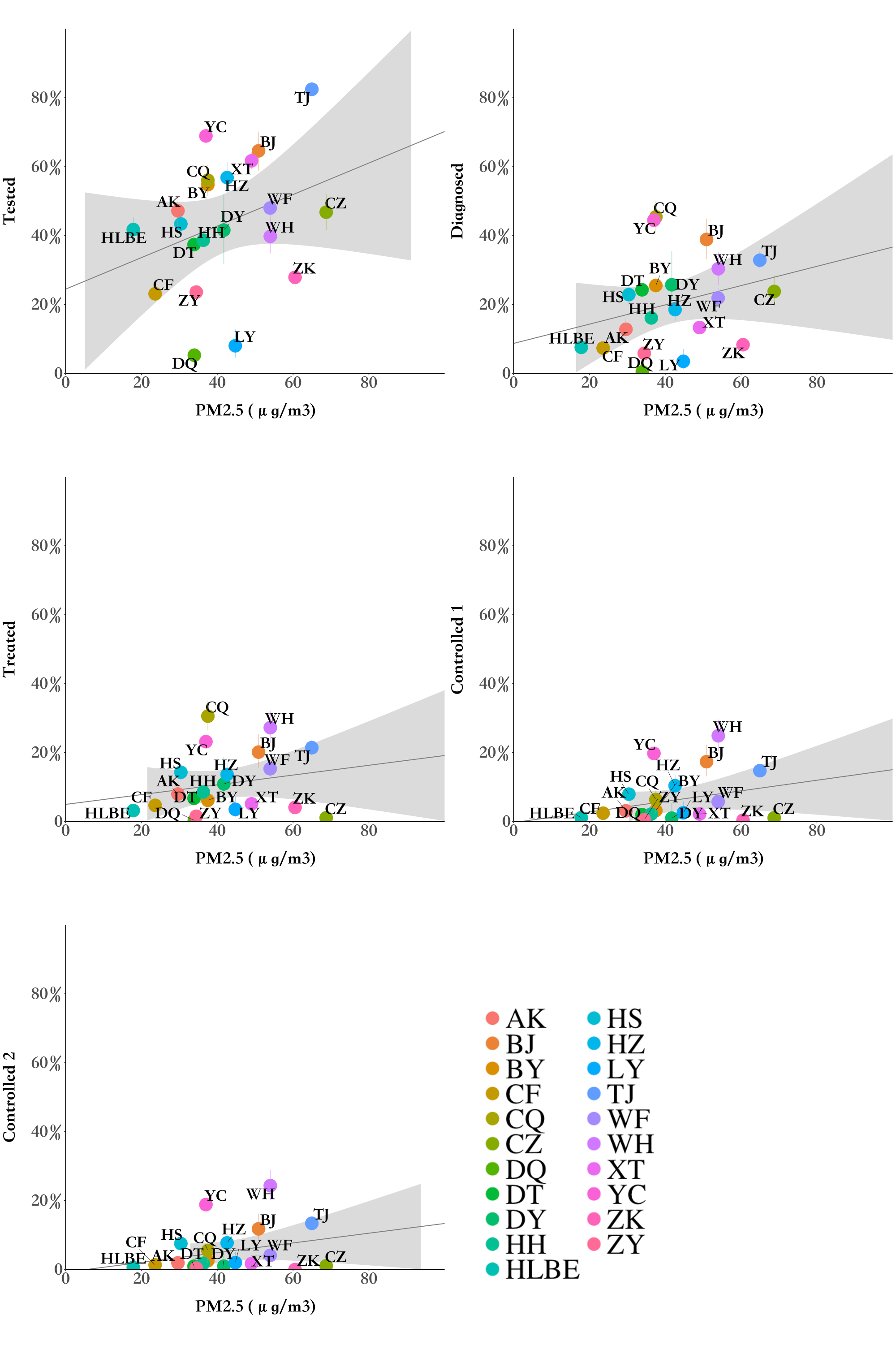


Table S10. Multivariable regression when excluding observations from Zunyi. Note: Controlled 1 = Controlled (mild or no exacerbations), Controlled 2 = Controlled (no exacerbations).

| **Variable** | **Tested** | | **Diagnosed** | | **Treated** | | **Controlled 1** | | **Controlled 2** | |
| --- | --- | --- | --- | --- | --- | --- | --- | --- | --- | --- |
|  | RR (95% CI) | p-value | RR (95% CI) | p-value | RR (95% CI) | p-value | RR (95% CI) | p-value | RR (95% CI) | p-value |
| **Sex** | | | |  |  |  |  |  |  |  |
| Female | 1 (ref) | - | 1 (ref) | - | 1 (ref) | - | 1 (ref) | - | 1 (ref) | - |
| Male | 1.16 (1.08, 1.24) | <0·001 | 1.17 (1.05, 1.30) | 0.01 | 1.32 (1.11, 1.57) | 0·00 | 1.30 (1.10, 1.55) | 0·00 | 1.30 (1.11, 1.53) | 0·00 |
| **Age, years** |  |  |  |  |  |  |  |  |  |  |
| 54 and below | 1 (ref) | - | 1 (ref) | - | 1 (ref) | - | 1 (ref) | - | 1 (ref) | - |
| 55-64 | 1.21 (1.10, 1.33) | <0·001 | 2.05 (1.82, 2.30) | <0·001 | 2.06 (1.75, 2.42) | <0·001 | 2.04 (1.71, 2.43) | <0·001 | 1.87 (1.56, 2.24) | <0·001 |
| 65 and above | 1.32 (1.18, 1.48) | <0·001 | 2.4 (2.13, 2.84) | <0·001 | 2.48 (2.10, 2.92) | <0·001 | 2.12 (1.64, 2.74) | <0·001 | 2.00 (1.52, 2.63) | <0·001 |
| **Education** |  |  |  |  |  |  |  |  |  |  |
| Primary education and below | 1 (ref) | - | 1 (ref) | - | 1 (ref) | - | 1 (ref) | - | 1 (ref) | - |
| Secondary education | 1.04 (0.93, 1.17) | 0.45 | 0.98 (1.01, 1.26) | 0.11 | 1.33 (1.13, 1.56) | 0·00 | 1.53 (1.31, 1.79) | <0·001 | 1.60 (1.36, 1.89) | <0·001 |
| College education | 1.18 (0.98, 1.41) | 0.08 | 1.22 (0.94, 1.59) | 0.13 | 1.43 (1.19, 1.71) | <0·001 | 1.37 (1.11, 1.69) | 0·00 | 1.34 (1.05, 1.71) | 0.02 |
| **Body-mass index group** |  |  |  |  |  |  |  |  |  |  |
| Underweight | 1.09 (1.02, 1.16) | 0·00 | 1.35 (1.20, 1.52) | <0·001 | 1.17 (1.00, 1.26) | 0.06 | 1.01 (0.77, 1.33) | 0.94 | 0.98 (0.75, 1.29) | 0.88 |
| Healthy weight | 1 (ref) | - | 1 (ref) | - | 1 (ref) | - | 1 (ref) | - | 1 (ref) | - |
| Overweight | 0.95 (0.90, 1.00) | 0.05 | 0.95 (0.87, 1.01) | 0.12 | 0.92 (0.84, 1.01) | 0.10 | 0.93 (0.80, 1.09) | 0.37 | 0.94 (0.79, 1.12) | 0.52 |
| Obese | 0.92 (0.82, 1.04) | 0.20 | 0.83 (0.71, 0.97) | 0.02 | 0.79 (0.67, 0.95) | 0.01 | 0.76 (0.62, 0.94) | 0.01 | 0.78 (0.65, 0.93) | 0.01 |
| **Tobacco smoking** |  |  |  |  |  |  |  |  |  |  |
| Never smoked | 1 (ref) | - | 1 (ref) | - | 1 (ref) | - | 1 (ref) | - | 1 (ref) | - |
| Ever smoked | 0.97 (0.91, 1.03) | 0.37 | 1.13 (1.03, 1.23) | 0.57 | 1.04 (0.87, 1.26) | 0.66 | 1.02 (0.83, 1.26) | 0.85 | 1.05 (0.84, 1.32) | 0.63 |
| **Urbanicity** |  |  |  |  |  |  |  |  |  |  |
| Rural townships | 1 (ref) | - | 1 (ref) | - | 1 (ref) | - | 1 (ref) | - | 1 (ref) | - |
| Urban areas | 1.12 (0.99, 1.27) | 0.07 | 1.04 (0.84, 1.28) | 0.73 | 1.22 (0.99, 1.52) | 0.07 | 1.41 (1.18, 1.68) | <0·001 | 1.49 (1.20, 1.86) | <0.001 |

Figure S8. COPD care cascade outcomes across regions by age group in the Happy Breathing Programme in China. Note: Data are percentages with 95% CIs. Controlled 1 = Controlled (mild or no exacerbations), Controlled 2 = Controlled (no exacerbations).


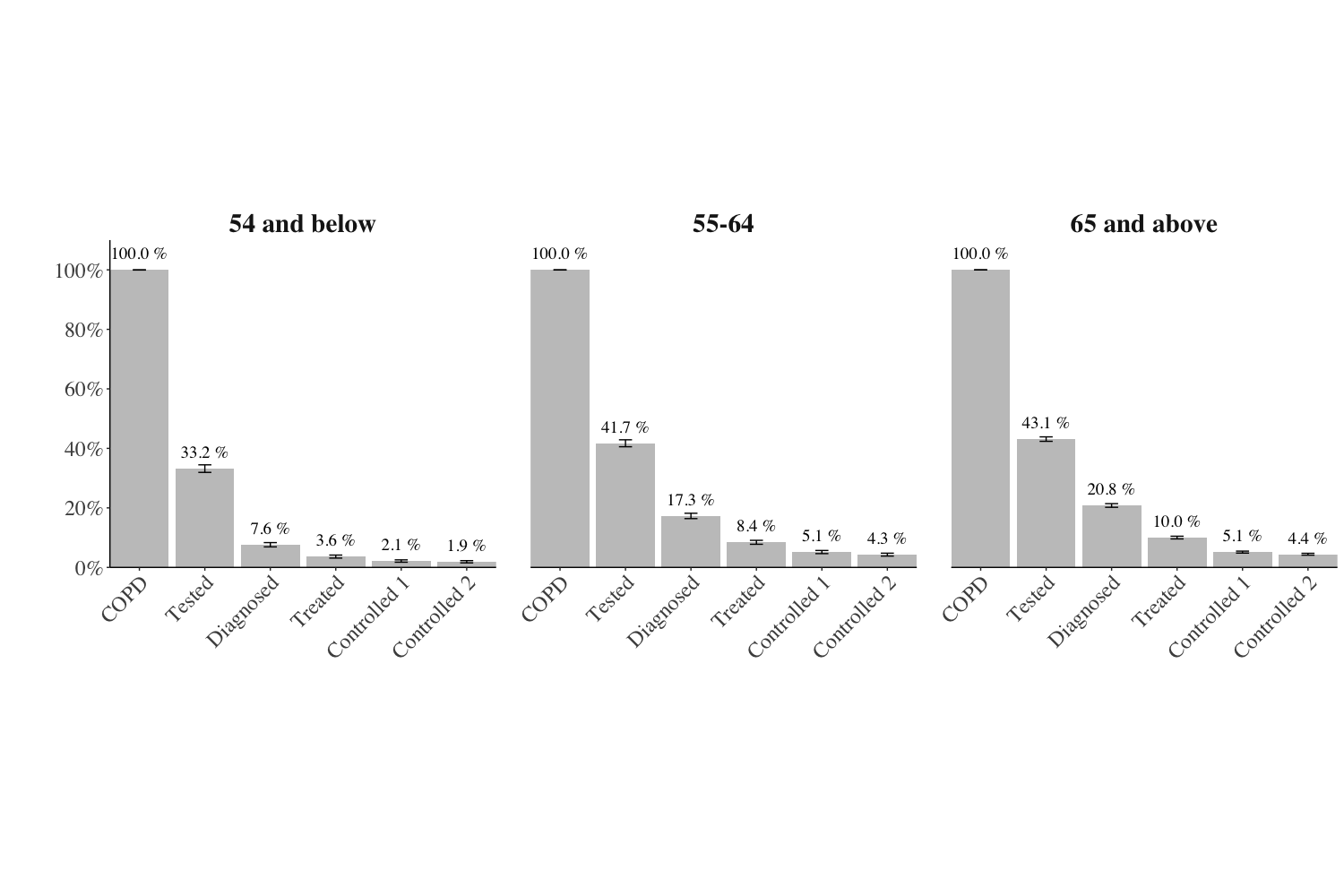


Figure S9. COPD care cascade outcomes in each region by age group in the ‘Happy Breathing’ Programme in China. Note: Controlled 1 = Controlled (mild or no exacerbations), Controlled 2 = Controlled (no exacerbations). This figure only shows the COPD care cascade in regions with sample size over 100.

**
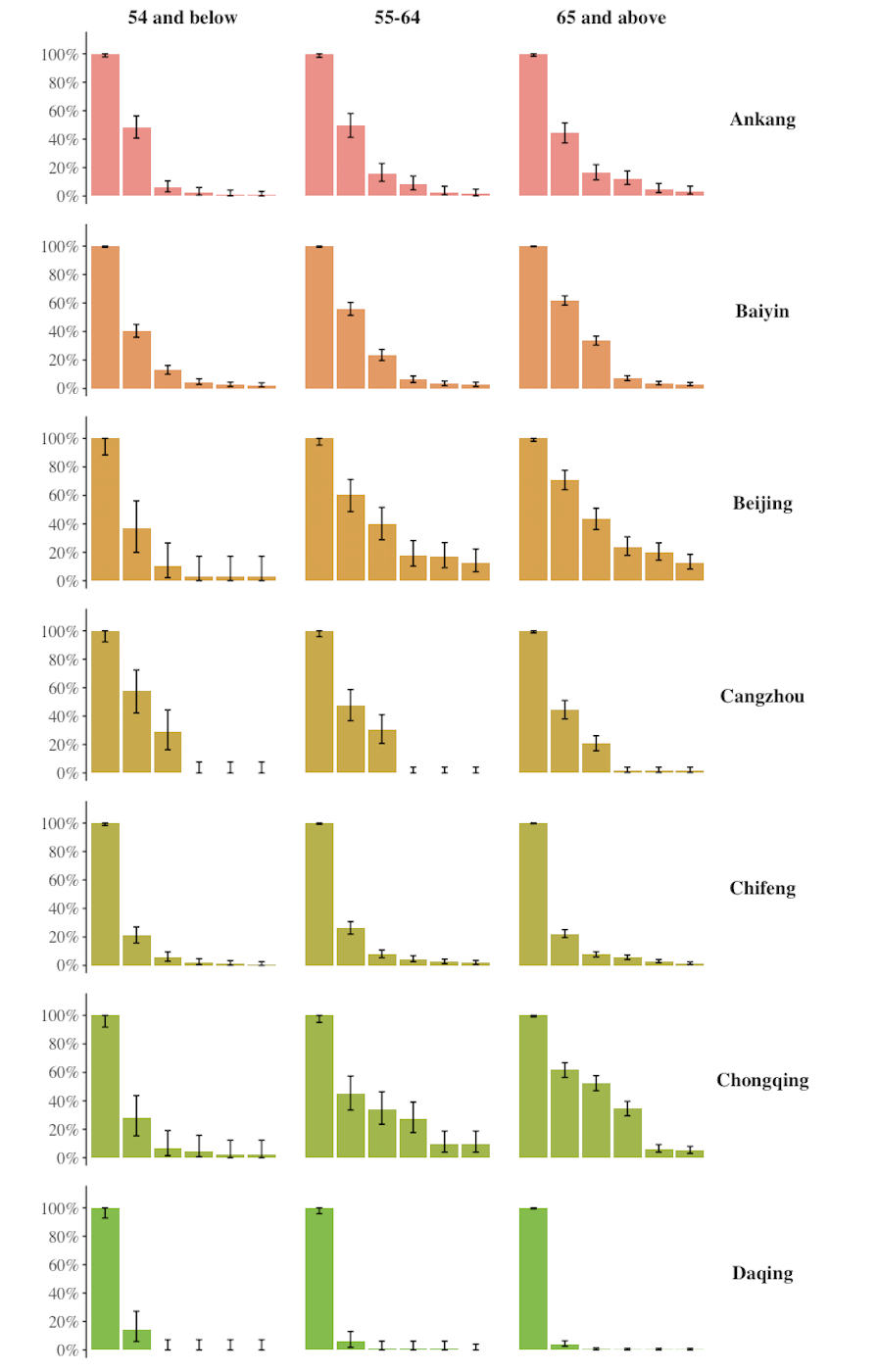
**


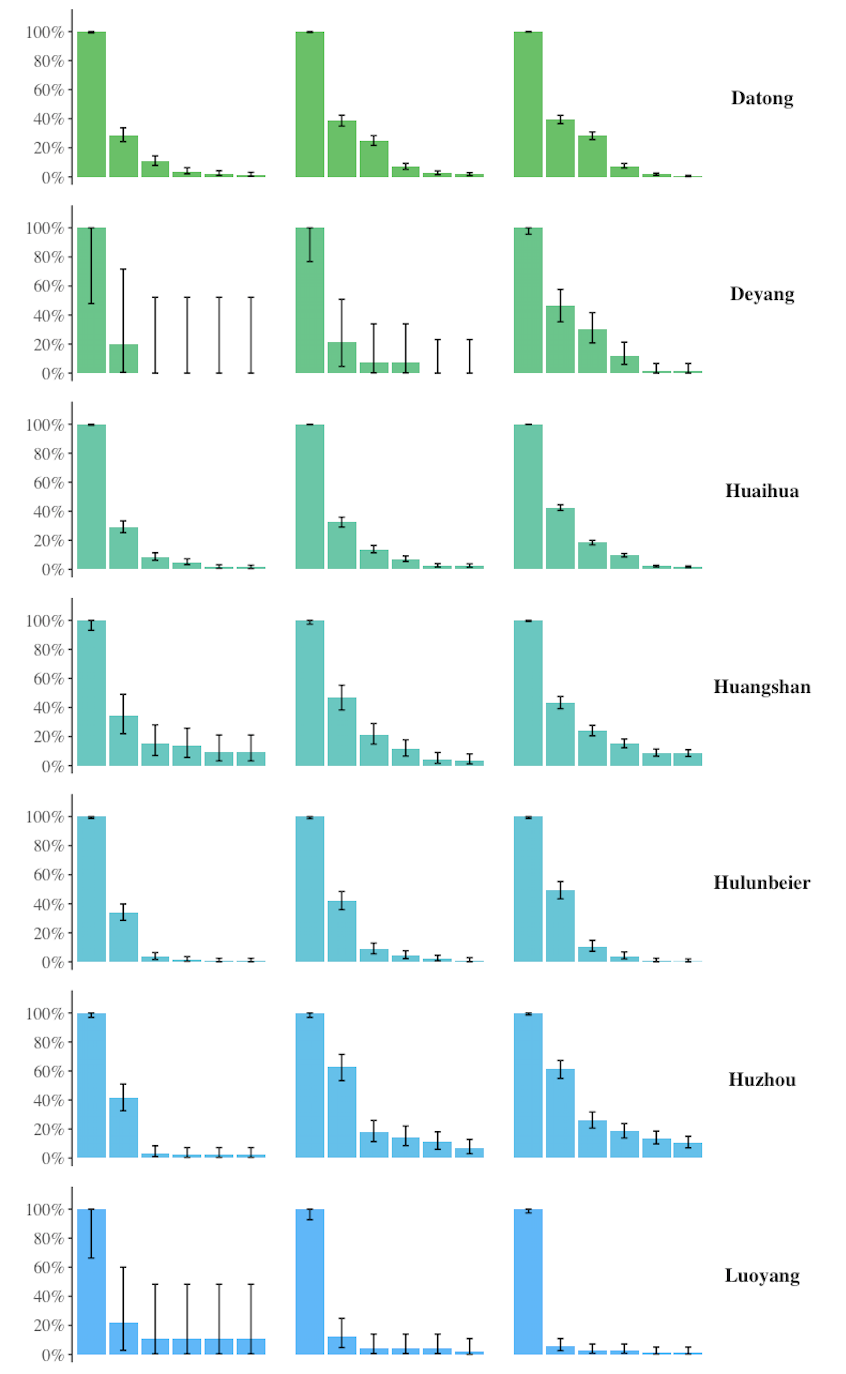


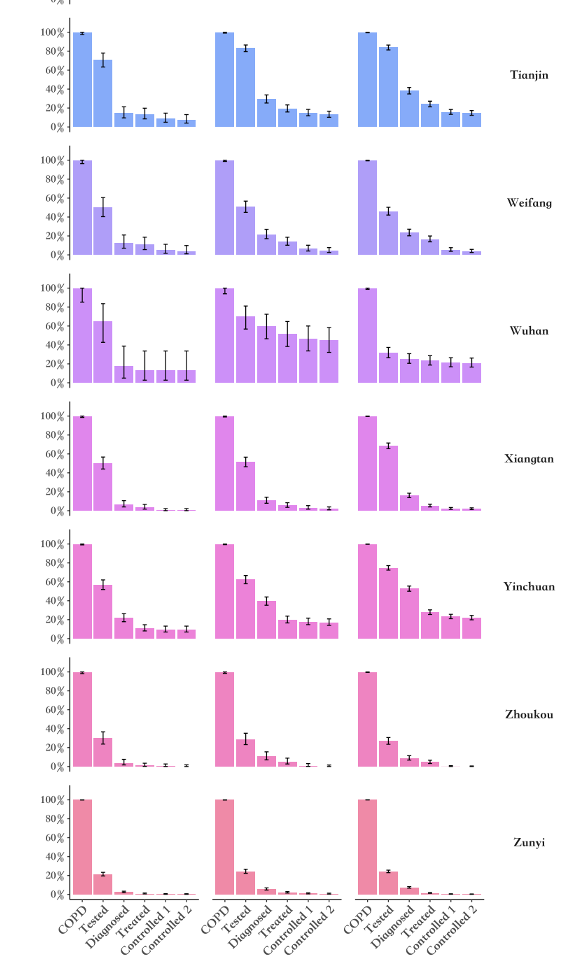

Supplement: Supplementary Appendix [file mmc1.docx]
